# Regulation of mitochondrial proteostasis by the proton gradient

**DOI:** 10.15252/embj.2021110476

**Published:** 2022-08-01

**Authors:** Maria Patron, Daryna Tarasenko, Hendrik Nolte, Lara Kroczek, Mausumi Ghosh, Yohsuke Ohba, Yvonne Lasarzewski, Zeinab Alsadat Ahmadi, Alfredo Cabrera‐Orefice, Akinori Eyiama, Tim Kellermann, Elena I Rugarli, Ulrich Brandt, Michael Meinecke, Thomas Langer

**Affiliations:** ^1^ Max Planck Institute for Biology of Ageing Cologne Germany; ^2^ Department of Cellular Biochemistry University Medical Center Göttingen Göttingen Germany; ^3^ Heidelberg University Biochemistry Center (BZH) Heidelberg Germany; ^4^ Radboud Institute for Molecular Life Sciences Radboud University Medical Center Nijmegen The Netherlands; ^5^ Cologne Excellence Cluster on Cellular Stress Responses in Aging‐Associated Diseases (CECAD) University of Cologne Cologne Germany; ^6^ Institute for Genetics University of Cologne Cologne Germany

**Keywords:** AFG3L2, mitochondrial calcium, proton gradient, respiratory chain, TMBIM5, Membranes & Trafficking, Metabolism, Post-translational Modifications & Proteolysis

## Abstract

Mitochondria adapt to different energetic demands reshaping their proteome. Mitochondrial proteases are emerging as key regulators of these adaptive processes. Here, we use a multiproteomic approach to demonstrate the regulation of the m‐AAA protease AFG3L2 by the mitochondrial proton gradient, coupling mitochondrial protein turnover to the energetic status of mitochondria. We identify TMBIM5 (previously also known as GHITM or MICS1) as a Ca^2+^/H^+^ exchanger in the mitochondrial inner membrane, which binds to and inhibits the m‐AAA protease. TMBIM5 ensures cell survival and respiration, allowing Ca^2+^ efflux from mitochondria and limiting mitochondrial hyperpolarization. Persistent hyperpolarization, however, triggers degradation of TMBIM5 and activation of the m‐AAA protease. The m‐AAA protease broadly remodels the mitochondrial proteome and mediates the proteolytic breakdown of respiratory complex I to confine ROS production and oxidative damage in hyperpolarized mitochondria. TMBIM5 thus integrates mitochondrial Ca^2+^ signaling and the energetic status of mitochondria with protein turnover rates to reshape the mitochondrial proteome and adjust the cellular metabolism.

## Graphical abstract

TMBIM5‐driven Ca^2+^/H^+^ exchange couples mitochondrial protein turnover to energetic status and metabolism.

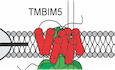

## Introduction

Mitochondria are the major site of cellular ATP production in aerobic organisms and provide numerous metabolites to ensure cell survival, proliferation, and differentiation. Mitochondrial integrity depends on multiple intra‐organellar proteases, which preserve proteostasis and are emerging as key regulators of mitochondrial plasticity (Deshwal *et al*, 2020; Song *et al*, 2021). This is exemplified by *m*‐AAA proteases, conserved and ubiquitously expressed ATP‐dependent metallopeptidases in the inner membrane (IM) of mitochondria (Levytskyy *et al*, 2017; Patron *et al*, 2018). m‐AAA proteases form homo‐ or hetero‐oligomeric, hexameric complexes, which are built up of homologous AFG3L2‐ or AFG3L2 and SPG7 (paraplegin) subunits, respectively. Mutations in these subunits are associated with pleiotropic neurodegenerative disorders, ranging from dominant spinocerebellar ataxia (SCA28), recessive hereditary spastic paraplegia (HSP type 7) to dominant optic atrophy (Casari *et al*, 1998; Di Bella *et al*, 2010; Pierson *et al*, 2011; Pfeffer *et al*, 2014; Charif *et al*, 2020).

The role of the m‐AAA protease for protein quality control was originally recognized in yeast (Arlt *et al*, 1996; Leonhard *et al*, 1999) and later shown to be conserved in mammalian cells, where nonassembled and damaged OXPHOS subunits are degraded by the m‐AAA protease (Hornig‐Do *et al*, 2012; Zurita Rendon *et al*, 2014). The loss of m‐AAA protease subunits SPG7 or AFG3L2 causes respiratory deficiencies, reduced respiratory complex I activity, and increased sensitivity to oxidative stress (Atorino *et al*, 2003; Ehses *et al*, 2009; Almajan *et al*, 2012). Aberrant protein accumulation in the IM reduces the mitochondrial membrane potential (ΔΨ_m_) and causes mitochondrial fragmentation (Ehses *et al*, 2009; Richter *et al*, 2015). The functional analysis of m‐AAA protease deficient cells revealed diverse roles of the m‐AAA protease going far beyond its role for protein quality control and the removal of damaged proteins. Processing of MRPL32, a subunit of mitochondrial ribosomes, by the m‐AAA protease allows ribosome assembly and mitochondrial translation (Nolden *et al*, 2005; Almajan *et al*, 2012). Moreover, the m‐AAA protease ensures the assembly of the mitochondrial calcium uniporter (MCU) complex in the IM by degrading excess EMRE subunits (König *et al*, 2016; Tsai *et al*, 2017). This prevents the formation of MCU complexes that lack the regulatory subunits MICU1 and MICU2, counteracting uncontrolled Ca^2+^ influx into mitochondria, mitochondrial Ca^2+^ overload, and cell death. The m‐AAA protease thus maintains mitochondrial Ca^2+^ homeostasis and ensures cell survival (Patron *et al*, 2018), which may also be of relevance for neurodegeneration (Maltecca *et al*, 2015).

Although studies in various mouse models and cultured cells revealed critical functions of m‐AAA proteases within mitochondria, it remained enigmatic how the m‐AAA protease activity is regulated. Here, we identify TMBIM5 (Transmembrane BAX Inhibitor‐1 Motif 5; previously also known as GHITM or MICS1; Li *et al*, 2001; Oka *et al*, 2008) as a novel interactor and inhibitor of the m‐AAA protease. We demonstrate that TMBIM5 acts as a Ca^2+^/H^+^ exchanger in the IM, which limits mitochondrial hyperpolarization and ROS production and couples the activity of the m‐AAA protease to the energetic demands of the cell. Persistent hyperpolarization causes degradation of TMBIM5 and leads to complex I degradation and broad reshaping of the mitochondrial proteome by the m‐AAA protease.

## Results

### Identification of TMBIM5 as a novel interactor of the m‐AAA protease

**Figure 1:**
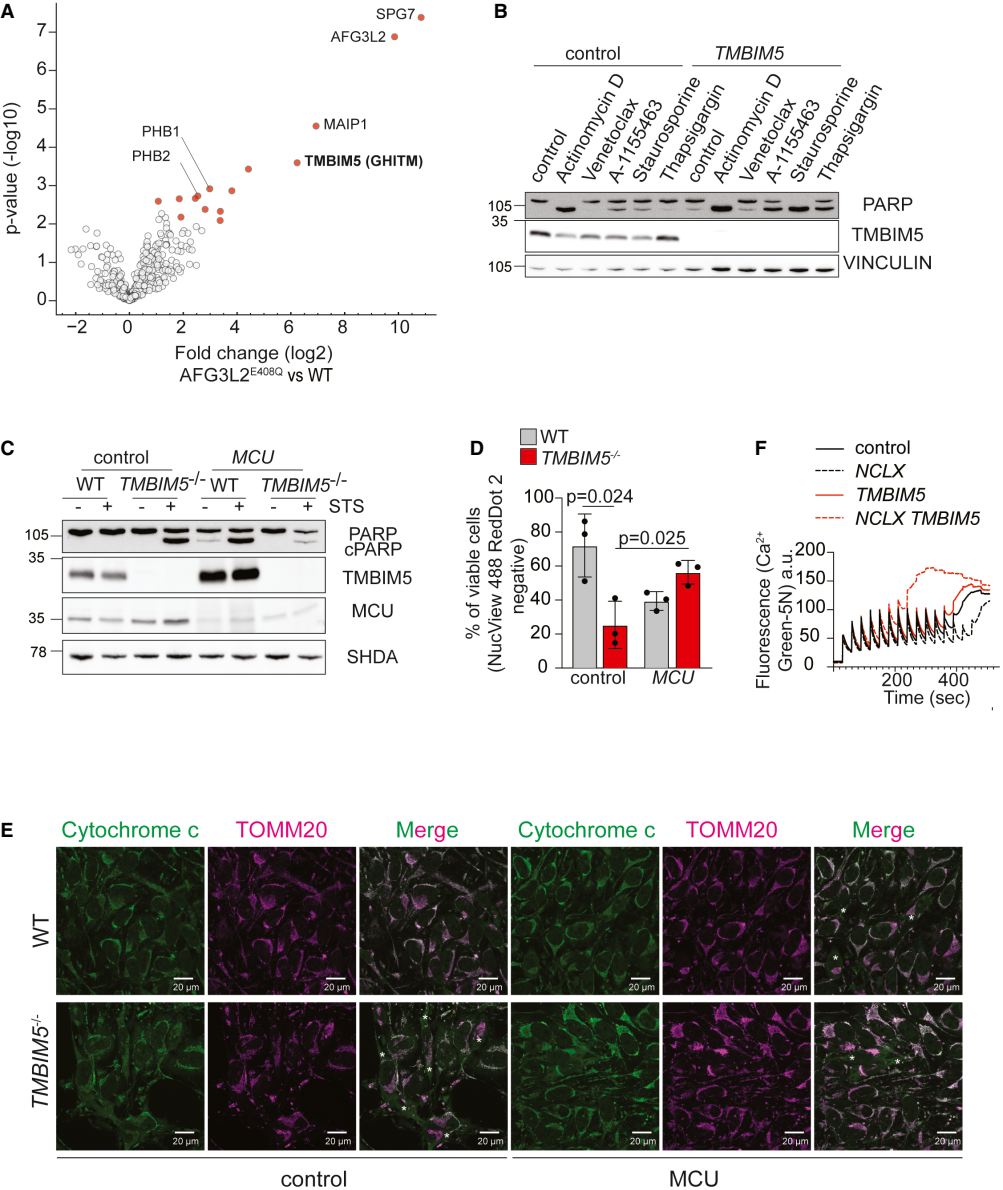
Mitochondrial Ca^2+^ overload triggers apoptotic death of *TMBIM5* ^−/−^ cells AVolcano plot representation of AFG3L2^E408Q^‐FLAG interaction partners. Mitochondria isolated from Flp‐In HEK293T‐REx cells expressing AFG3L2^E408Q^‐FLAG were subjected to immunoprecipitation. Co‐purifying proteins were identified by quantitative mass spectrometry (MS) (*n* = 5 independent experiments) followed by an unpaired two‐sided *t*‐test and permutation‐based FDR estimation to correct for multiple testing (FDR < 0.05). Significantly enriched MitoCarta 3.0 proteins (Rath *et al*, 2021) are colored in red, gene names indicate previously verified AFG3L2 interactors and TMBIM5 (GHITM, MICS1). See also Dataset EV1.BRepresentative immunoblot of HeLa wildtype (WT) cells transfected with scrambled siRNA (control) or siRNA targeting *TMBIM5* and treated with the indicated drugs for 16 h. Control (0.1% DMSO), actinomycin D (1 μM), venetoclax (1 μM), A‐1155463 (1 μM), staurosporine (1 μM), thapsigargin (2 μM). *n* = 3 independent experiments.CRepresentative immunoblot of HeLa WT and *TMBIM5* ^−/−^ cells transfected with scrambled siRNA (control) or siRNA targeting *MCU* for 72 h. Where indicated samples were treated with staurosporine (STS; 1 μM) for 16 h. Quantification is shown in Fig EV1G (*n* = 3 independent experiments).DViability of WT and *TMBIM5* ^−/−^ cells (depleted of MCU when indicated) after incubation with staurosporine (STS; 0.1 μM) for 16 h. Cell viability was assessed cytofluorimetrically monitoring NucView488 and RedDot2 staining and expressed as a percentage of all cells. *n* = 3 independent experiments; two‐tailed *t*‐test; mean ± SD. A *P*‐value of < 0.05 was considered statistically significant.EWT and *TMBIM5* ^−/−^ HeLa cells were transfected with the indicated scrambled siRNA (control) and siRNA targeting *MCU* for 48 h and incubated with staurosporine (0.1 μM) for 16 h in the presence of Z‐VAD‐FMK (50 μM) and epoxomicin (1 μM) to prevent apoptosis. Cells were subjected to immunofluorescence analysis with antibodies directed against cytochrome *c* (green) and TOMM20 (magenta). *Cells with cytosolic cytochrome *c*. Scale bar 20 µm. Quantification is shown in Fig EV1H (*n* = 3 independent experiments; number of cells > 150 each experiment).FCalcium retention capacity (CRC) was assessed in isolated mitochondria of HEK293T WT cells transfected with the indicated siRNA. The experiment was performed in a sucrose‐based medium containing respiratory substrates and the membrane‐impermeable Ca^2+^ sensor Ca^2+^ Green‐5N. Ca^2+^ Green‐5N fluorescence was monitored following repeated addition of Ca^2+^ pulses (10 μM). Data show representative traces (2 independent experiments run in triplicate for each condition). Data information: See also Fig EV1 and Dataset EV1. Source data are available online for this figure.

**Figure EV1:**
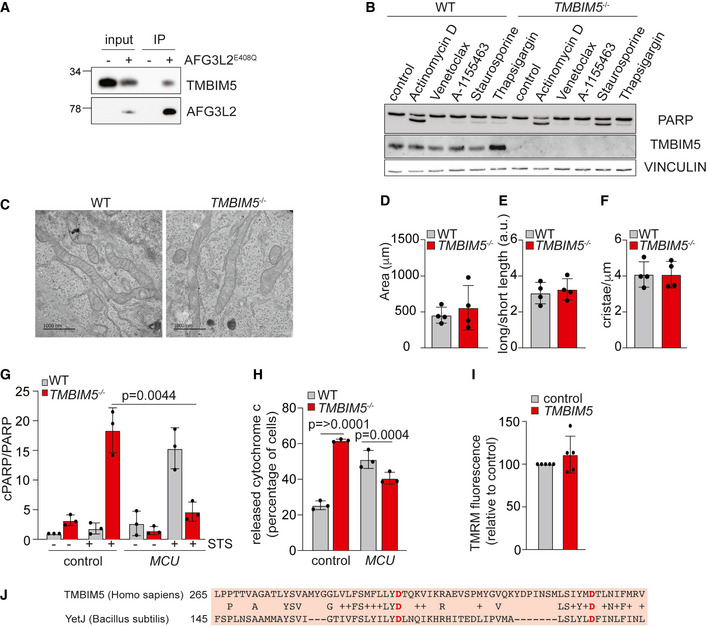
Mitochondrial Ca^2+^ overload triggers apoptotic death of *TMBIM5* ^−/−^ cells (Related to Fig 1) ARepresentative immunoblot of immunoprecipitates using anti‐FLAG M2 beads of mitochondrial lysates, which were isolated from Flp‐In HEK293T‐REx cells and from Flp‐In HEK293T‐REx cells expressing AFG3L2^E408Q FLAG^.BRepresentative immunoblot of wildtype (WT) and *TMBIM5* ^−/−^ HeLa cells treated with the indicated drugs for 16 h. Control (0.1% DMSO), actinomycin D (1 μM), venetoclax (1 μM), A‐1155463 (1 μM), staurosporine (1 μM), thapsigargin (2 μM). *n* = 3 independent experiments.CRepresentative transmission electron microscopy images of WT and *TMBIM5* ^−/−^ HeLa cells. Mitochondrial morphology is shown. Scale bar 1 μm. (*n* = 4 independent biological replicates, 300 mitochondria of at least six cells, mean ± SD).DQuantification of the electron microscopy images of WT and *TMBIM5* ^−/−^ HeLa cells shown in Fig EV1C. Mitochondrial parameters (area, length, and cristae) were calculated with FiJi. Values are mean ± SD.EQuantification of the electron microscopy images of WT and *TMBIM5* ^−/−^ HeLa cells shown in Fig EV1C. The ratio between the long length and the short length of mitochondria is shown. Values are mean ± SD.FQuantification of electron microscopy images of WT and *TMBIM5* ^−/−^ HeLa cells shown in Fig EV1C. Number of cristae per mitochondrial length (μm) is shown. Values are mean ± SD.GQuantification of Fig 1C. The ratio between cleaved PARP (cPARP; 89 kDa) and PARP (116 kDa). WT and *TMBIM5* ^−/−^ HeLa cells transfected with scrambled siRNA (control) or siRNA targeting *MCU* for 72 h. When indicated, samples were treated with staurosporine (STS; 1 μM) for 16 h. *n* = 3 independent experiments. Data are shown as mean ± SD.HQuantification of WT and *TMBIM5* ^−/−^ HeLa cells with cytoplasmic cytochrome *c*. After transfection with scrambled siRNA (control) or siRNA targeting *MCU* for 72 h cells were incubated for 16 h with staurosporine (0.1 μM) in the presence of Z‐VAD‐FMK (50 μM) and epoxomicin (1 μM) to prevent apoptosis. Cells were analyzed by immunofluorescence microscopy using antibodies directed against cytochrome *c* (green) and TOMM20 (magenta). *n* = 3 independent experiments; number of cells > 150 in each experiment. Mean ± SD.IMitochondrial membrane potential was monitored by TMRM staining in WT HeLa cells transfected with scrambled siRNA (control) or siRNA targeting *TMBIM5* for 72 h. Fluorescent intensity was calculated relative to the value upon CCCP addition (15 μM). Values are expressed as mean relative to control. Measurements of five different preparations were performed in triplicate. Mean ± SD.JSequence alignment of TMBIM5 (Q9H3K2, *Homo sapiens*) and YetJ (O31539, *Bacillus subtilis*). Conserved amino acids between the two species are written in the middle line. Amino acids forming the di‐aspartyl dyad are indicated. Source data are available online for this figure.

In an attempt to identify interacting proteins and substrates of human AFG3L2, we immunoprecipitated AFG3L2 from mitochondria of Flp‐In HEK293 T‐REx cells expressing proteolytically inactive AFG3L2^E408^‐FLAG. Mitochondrial lysates from HEK293 T‐REx cells were used for control. The analysis of proteins co‐purifying with AFG3L2^E408^‐FLAG by mass spectrometry identified paraplegin (SPG7), a proteolytic subunit of the *m*‐AAA protease complex, and known *m*‐AAA protease binding proteins, including the prohibitin membrane scaffold complex (PHB1, PHB2) and MAIP1 (Fig 1A; Dataset EV1). Besides these expected interactors, TMBIM5 (also known as GHITM or MICS1) was highly enriched in AFG3L2 precipitates (Fig 1A; Dataset EV1), which was further validated by immunoblot analysis (Fig EV1A). These experiments identified TMBIM5 as a novel constituent of *m*‐AAA protease‐containing complexes.

### Mitochondrial Ca^2+^ overload triggers apoptotic death of TMBIM5 ^−/−^ cells

TMBIM5 belongs to an evolutionary conserved family of six membrane proteins (TMBIM1‐6), which contain the transmembrane BAX inhibitor motif, originally described as the founding member of this family BI‐1 (or TMBIM6). TMBIM5 was originally identified as a deregulated protein upon growth factor withdrawal in mice (Li *et al*, 2001) and was recently found in CRISPR/Cas9 screens to be essential in human cells cultivated on a physiological medium (Rossiter *et al*, 2021). In contrast to other TMBIM proteins, TMBIM5 is localized to the IM, where it protects against apoptosis (Oka *et al*, 2008; Seitaj *et al*, 2020). In agreement with these findings, we observed an increased susceptibility of HeLa cells depleted of TMBIM5 to different apoptotic stimuli, when monitoring PARP cleavage (Fig 1B). Similarly, *TMBIM5* ^−/−^ HeLa cells, which were generated by CRISPR/Cas9‐mediated genome editing, were more sensitive to apoptotic cell death induced by staurosporine (Fig EV1B), as has been observed in TMBIM5‐deficient HAP1 cells (Seitaj *et al*, 2020). It was suggested that TMBIM5 preserves cristae morphogenesis and interacts with cytochrome *c*, limiting its release into the cytoplasm in apoptotic cells. However, *TMBIM5* ^−/−^ HeLa cells harbored a normal, reticulated mitochondrial network with unaltered cristae (Fig EV1C–F), suggesting that other mechanisms lead to the increased apoptotic vulnerability of *TMBIM5* ^−/−^ cells.

Members of the TMBIM family function as pH‐dependent Ca^2+^ leak channels and regulate both Ca^2+^ homeostasis and cell death (Kim *et al*, 2008; Rojas‐Rivera & Hetz, 2015; Liu, 2017). To investigate the possibility that altered mitochondrial Ca^2+^ homeostasis causes the increased vulnerability of *TMBIM5* ^−/−^ cells, we monitored PARP cleavage as a readout for cell death in these cells after silencing the mitochondrial calcium uniporter, MCU. Depletion of MCU from *TMBIM5* ^−/−^ cells reduced PARP cleavage upon treatment with staurosporine (Figs 1C and EV1G). Moreover, MCU depletion significantly reduced the number of apoptotic or necrotic *TMBIM5* ^−/−^ cells harboring cleaved caspase 3/7 (Fig 1D) and the release of cytochrome *c* from TMBIM5‐deficient mitochondria to the cytosol (Figs 1E and EV1H). Therefore, limiting MCU‐dependent mitochondrial Ca^2+^ uptake protects *TMBIM5* ^−/−^ cells against apoptosis, suggesting that cell death is induced by mitochondrial Ca^2+^ overload.

To corroborate these findings, we determined the Ca^2+^ retention capacity (CRC) of mitochondria depleted of TMBIM5, which were isolated from HEK293T cells. To this end, we monitored Ca^2+^ influx after repeated Ca^2+^ pulses using the Ca^2+^‐dependent fluorescent indicator Calcium‐Green 5N (Fig 1F). The loss of TMBIM5 alone did not significantly affect the CRC but resulted in lowered CRC and earlier opening of the membrane permeability transition pore (MPTP) upon additional depletion of the mitochondrial Na^+^/Ca^2+^ exchanger NCLX (Fig 1F). Thus, HEK293T cells depleted of TMBIM5 are more susceptible to mitochondrial Ca^2+^ overload. Ca^2+^ export by NCLX can at least partially compensate for the loss of TMBIM5, indicating that loss of TMBIM5 reduces Ca^2+^ leakage from mitochondria rather than increasing Ca^2+^ influx.

### TMBIM5 acts as a Ca^2+^/H^+^ exchanger in the inner membrane

**Figure 2:**
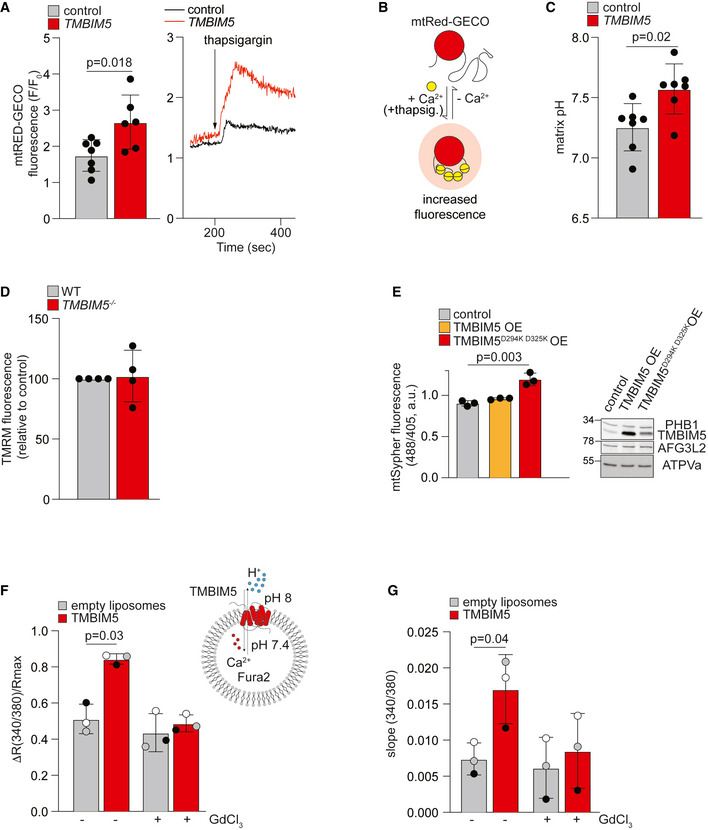
TMBIM5 acts as a Ca^2+^/H^+^ exchanger AFluorescence (F) F/F_0_ ratio quantifications, where F_0_ is the average of the first 10 s and F is the fluorescence peak measured after thapsigargin (2 μM) administration (left panel). The right shows mtRED‐GECO ratio traces of HeLa cells transfected with scrambled siRNA (control) and siRNA targeting *TMBIM5*. Each measurement was performed in at least 10 cells from six different preparations. Mean ± SD; two‐tailed *t*‐test. A *P*‐value of < 0.05 was considered statistically significant.BSchematic representation of experiments shown in (A).CMatrix pH was measured with Sypher3smito in wildtype (WT) HeLa cells transfected with scrambled siRNA (control) or siRNA targeting *TMBIM5* for 72 h. The probe was calibrated in KRB buffer by the stepwise addition/removal of permeant weak acid. Each measurement was performed in at least 80 cells from seven different preparations. Mean ± SD; two‐tailed *t*‐test. A *P*‐value of < 0.05 was considered statistically significant.DMitochondrial membrane potential was monitored with TMRM in WT and *TMBIM5* ^−/−^ HeLa cells. Fluorescent intensity was calculated as a ratio to the value upon CCCP addition (15 μM). Values are expressed as mean ± SD relative to control. Each measurement was performed in triplicate from four different preparations.EMatrix pH was measured with Sypher3smito in WT HeLa cells transiently expressing TMBIM5 or TMBIM5^D294K D325K^ for 24 h. The ratio of absorbance at 488 nm and 405 nm is shown in arbitrary units (a.u.). Each measurement was performed in at least 20 cells from three different preparations. Representative immunoblot of WT HeLa cells transfected with TMBIM5 or TMBIM5^D294K D325K^ for 24 h (right panel). Mean ± SD; two‐tailed *t*‐test. A *P*‐value of < 0.05 was considered statistically significant.FLiposome assay. Recombinant TMBIM5 was reconstituted in liposomes containing 100 μM Fura‐2. CaCl_2_ was added later at the concentration of 100 μM. R_max_ was obtained at the end of the experiment after the addition of ionomycin (10 μM). Data are expressed as mean of R/R_max_, where R is the peak after CaCl_2_ addition. Where indicated GdCl_3_ (100 μM) was added 10 min before the experiment. *n* = 3 independent experiments, shown as black, white, and gray dots. Mean ± SD; two‐tailed *t*‐test. A *P*‐value of < 0.05 was considered statistically significant.GLiposome assay (as in E). Slope was calculated in the 100 s after 100 μM CaCl_2_ addition as expressed as mean ± SD. *n* = 3 independent experiments, shown as black, white, and gray dots. Two‐tailed *t*‐test. A *P*‐value of < 0.05 was considered statistically significant. Data information: See also Fig EV2. Source data are available online for this figure.

To further investigate the role of TMBIM5 as a potential mitochondrial ion channel, we measured mitochondrial Ca^2+^ uptake after stimulating HeLa cells with thapsigargin, an inhibitor of the sarco‐endoplasmic reticulum calcium ATPase (SERCA). Thapsigargin treatment induces Ca^2+^ leakage from the ER and promotes mitochondrial Ca^2+^ uptake at ER‐mitochondria contact sites. We expressed an RFP‐based Ca^2+^ indicator that was specifically targeted to the mitochondrial matrix (mtRed‐GECO; Wu *et al*, 2013) in HeLa cells depleted of TMBIM5 and monitored fluorescence intensity after thapsigargin treatment (Fig 2A and B). TMBIM5 depleted cells accumulated Ca^2+^ within mitochondria when compared to control cells (Fig 2A), which is consistent with our hypothesis of reduced mitochondrial Ca^2+^ efflux in the absence of TMBIM5.

The founding member of the TMBIM family, TMBIM6 (or BI‐1) was suggested to be a pH‐sensitive Ca^2+^ leak channel in the ER membrane, which can be activated by cytosolic protonation of a di‐aspartyl moiety (Bultynck *et al*, 2014; Chang *et al*, 2014; Guo *et al*, 2019; Li *et al*, 2020). A Ca^2+^/H^+^ antiporter activity of TMBIM6 has been discussed controversially, mainly because a Ca^2+^/H^+^ exchanger can function only in the presence of a stable H^+^ gradient that is not present between the ER lumen and the cytosol. Since the di‐aspartyl pH sensor motif of TMBIM6 is conserved in TMBIM5 and since a H^+^ gradient is constantly present across the IM, we examined whether TMBIM5 can act as a Ca^2+^/H^+^ exchanger. We expressed a mitochondrially targeted variant of the synthetic pH sensor SypHer (SypHer3s‐dmito; Ermakova *et al*, 2018) in cells depleted of TMBIM5 and monitored the pH in the mitochondrial matrix (Fig 2C). Strikingly, loss of TMBIM5 increased the matrix pH from pH 7.25 to pH 7.6 (Fig 2C), indicating that the TMBIM5‐mediated Ca^2+^ release from mitochondria is indeed accompanied by H^+^ transport into the mitochondrial matrix. TMRM staining showed normal ΔΨ_m_ in *TMBIM5* ^−/−^ cells when compared to wildtype cells, excluding that the decreased matrix H^+^ content reflects differences in ΔΨ_m_ (Figs 2D and EV1I) (Chang *et al*, 2014; Guo *et al*, 2019). As stable overexpression of TMBIM5 causes the loss of ΔΨ_m_ (Oka *et al*, 2008), we expressed TMBIM5 only transiently in HeLa cells. While we did not observe an altered matrix pH upon expression of TMBIM5, we observed an increased matrix pH when compared to wildtype cells upon expression of TMBIM5^D294K D325K^, harboring mutations in the di‐aspartyl motif (Figs 2E and EV1J). Thus, expression of TMBIM5^D294K D325K^ mimics the phenotype of *TMBIM5* ^−/−^ cells, highlighting the importance of the di‐aspartyl motif for TMBIM5 function.

**Figure EV2:**
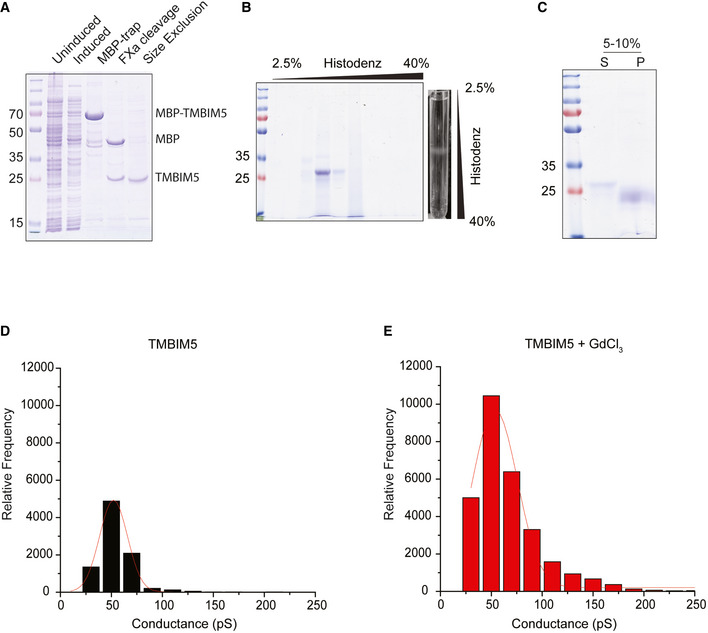
Reconstitution of TMBIM5 into liposomes (Related to Figs 2 and 3) ARepresentative Coomassie‐stained SDS‐polyacrylamide gel (12.5%) demonstrating enrichment of recombinant TMBIM5 protein upon purification.BFlotation assay demonstrating co‐migration of recombinant TMBIM5 protein with the liposomes after ultracentrifugation in a discontinuous histodenz gradient. A representative Coomassie‐stained SDS‐polyacrylamide gel (12.5%) is shown. The ultracentrifugation tube containing TMBIM5‐liposomes in discontinuous histodenz gradient after ultracentrifugation but before fractionation is shown on the right.CSodium bicarbonate extraction. Supernatant (S) and pellet (P) fractions of TMBIM5‐proteoliposomes after treatment with 0.1 M sodium bicarbonate (pH 11.5) analyzed by SDS‐polyacrylamide gel (12.5%) electrophoresis. Pellet (P), and supernatant (S) are indicated in the figure. The presence of TMBIM5 in the pellet fraction indicates its integration into the lipid bilayer.DTMBIM5 conductance state histogram in CaCl_2_ buffer. *n* = 3 independent experiments.ETMBIM5 conductance state histogram in CaCl_2_ buffer containing GdCl_2_. *n* = 3 independent experiments.

To unambiguously demonstrate the channel activity of TMBIM5, we purified TMBIM5 expressed in *Escherichia coli* and reconstituted the protein in liposomes harboring the membrane‐impermeable Ca^2+^ sensor Fura‐2 (Figs 2F and EV2A–C). After the addition of Ca^2+^ to liposomes, we observed increased Ca^2+^ influx into liposomes containing TMBIM5 when compared to control liposomes in the presence of a pH gradient (Fig 2F and G). Gadolinium chloride (GdCl_3_), a potent inhibitor of voltage‐gated Ca^2+^ channels, efficiently inhibited the TMBIM5‐dependent Ca^2+^ influx (Fig 2F and G). Thus, recombinant TMBIM5 is able to facilitate specifically the influx of Ca^2+^ into liposomes.

**Figure 3:**
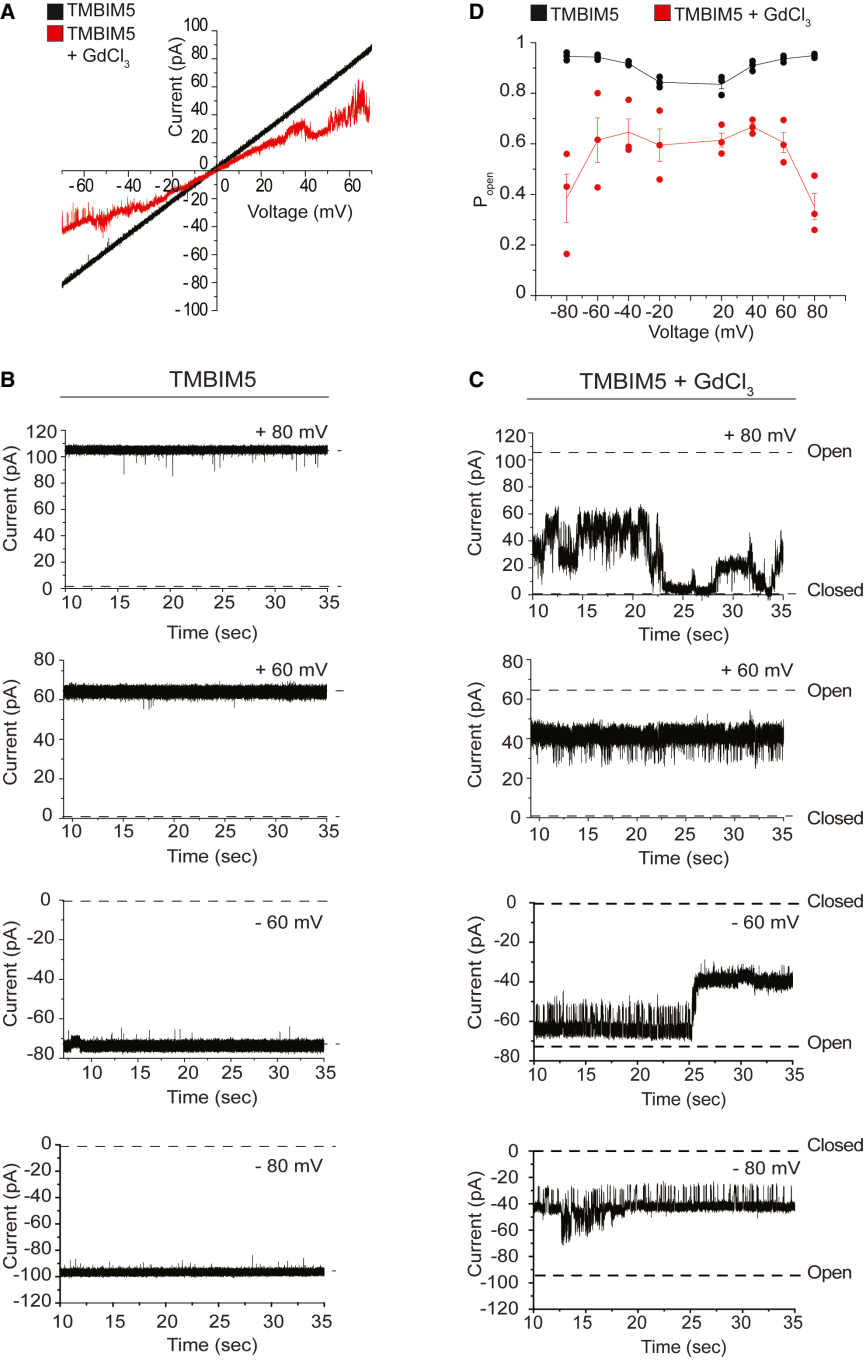
Reconstitution of TMBIM5 activity in lipid bilayers ACurrent–voltage relationship of multiple TMBIM5 channels in the absence (black) and presence (red) of GdCl_3_.BRepresentative current traces of multiple TMBIM5 channels inserted into planar lipid bilayers at indicated holding potentials.CRepresentative current traces of the same TMBIM5 channels inserted into planar lipid bilayers in the presence of GdCl_3_ at indicated holding potentials. Fast gating events and channel closure at elevated membrane potentials can be observed in the presence of GdCl_3_.DVoltage‐dependent open probability (*P*_open_) of TMBIM5 in the absence and presence of GdCl_3_. *n* = 3 independent channel incorporations. Mean ± SD. Data information: See also Fig EV2.

We then asked whether we can directly show the channel activity of TMBIM5. We fused TMBIM5 containing proteoliposomes with planar lipid membranes to perform high‐resolution electrophysiology measurements (Fig 3). Fusion events were observed and led to the stable insertion of channel proteins into planar lipid bilayers (Fig 3A). From a conductance state analysis based on single gating events, a main conductance state of ~ 50 pS (Fig EV2D and E) was found at 250 mM CaCl_2_. Considering a cylindrical pore with a restriction zone of ~ 1 nm in length as the structural basis and assuming a five‐fold higher solution resistance within the pore than in the bulk medium as described before (Smart *et al*, 1997), this corresponds to a channel diameter of ~ 0.5 nm. Occasionally, we observed larger gating events, suggesting broader dynamics in gating and pore size. The calculated pore size is in good agreement with the channel diameter at the restriction zone observed in crystal structures of a bacterial homolog of TMBIM5 (Chang *et al*, 2014). Interestingly, even when titrating down the protein to lipid ratio in proteoliposomes to be fused with planar lipid membrane, we were only able to measure multiple copies of the channel (Fig 3B), suggesting that TMBIM5 forms oligomers after reconstitution. As the channel activity was stable over a long period of time, we could monitor pore activity upon the addition of GdCl_3_, which inhibits Ca^2+^ uptake into liposomes. GdCl_3_ led to a significantly decreased conductivity and a tendency for gating towards the closed state at elevated applied voltages (Fig 3B and C). At holding potentials of +80 mV the channel displayed a 70% decreased open probability (Fig 3D), showing that calcium conductivity can be blocked by GdCl_3_.

Together, we conclude that TMBIM5 can act as a Ca^2+^/H^+^ exchanger in the IM, coupling mitochondrial export of Ca^2+^ with H^+^ import into mitochondria. Loss of TMBIM5 leads to matrix alkalinization, mitochondrial hyperpolarization, and the accumulation of Ca^2+^ within mitochondria, rendering cells sensitive to mitochondrial Ca^2+^ overload and cell death.

### TMBIM5 controls oxidative phosphorylation

**Figure 4:**
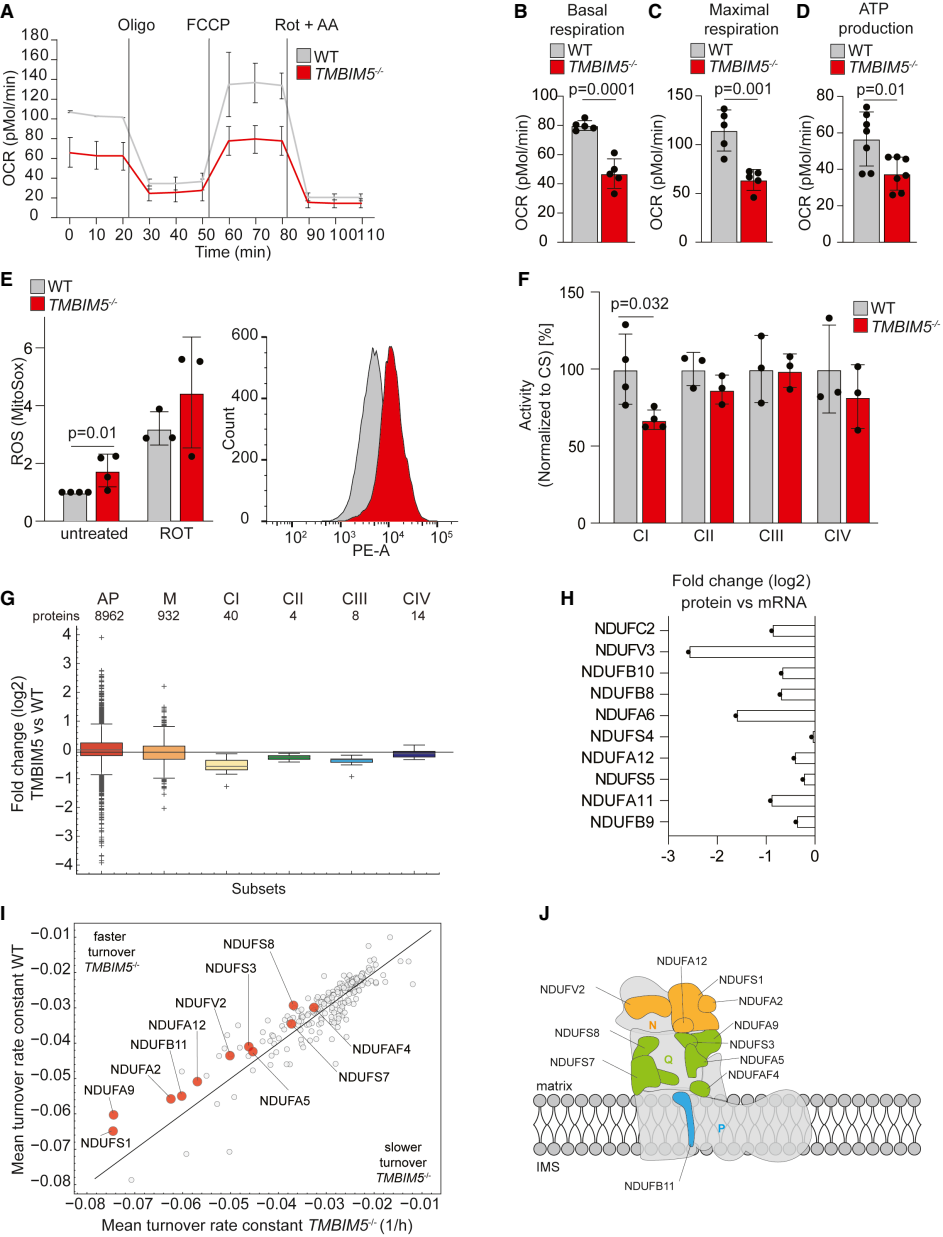
TMBIM5 controls oxidative phosphorylation AOxygen consumption rate (OCR) of wildtype (WT) and *TMBIM5* ^−/−^ HeLa cells in glucose‐containing media. Traces are mean ± SD of seven independent biological experiments each one run at least in triplicate. Labeled lines denote injections of oligomycin (Oligo, 2 μM), FCCP (0.5 μM), rotenone, and antimycin A (Rot + AA; both 0.5 μM).BBasal respiration calculated from OCR experiment of WT and *TMBIM5* ^−/−^ HeLa cells in (A). Data are shown as mean ± SD; two‐tailed *t*‐test. A *P*‐value of < 0.05 was considered statistically significant.CMaximal respiration calculated from OCR experiment of WT and *TMBIM5* ^−/−^ HeLa cells in (A). Data are shown as mean ± SD; two‐tailed *t*‐test. A *P*‐value of < 0.05 was considered statistically significant.DATP production calculated from OCR experiments of WT and *TMBIM5* ^−/−^ HeLa cells in (A). Data are shown as mean ± SD; two‐tailed *t*‐test. A *P*‐value of < 0.05 was considered statistically significant.EMitochondrial ROS production. WT and *TMBIM5* ^−/−^ HeLa cells were stained with MitoSox Red for 30 min and analyzed using fluorescence‐activated cell sorting (FACS). A FACS blot is shown in the right panel. Rotenone (0.5 μM) was added during MitoSox Red staining. Data are expressed as median (PE‐A) ± SD (*n* = 3–4 independent experiments). Two‐tailed *t*‐test. A *P*‐value of < 0.05 was considered statistically significant.FEnzymatic activities of different electron transport chain complexes (indicated as complex I = CI, complex II = CII, complex III = CIII, and complex IV = CIV) measured in freeze‐thawed mitochondria from HEK293T WT and *TMBIM5* ^−/−^ cells. Data are normalized to citrate synthase (CS) activities and expressed as mean ± SD percentage of control (*n* = 3–4 independent mitochondrial preparations, each one measured in 3–6 technical replicates). Two‐tailed *t*‐test. A *P*‐value of < 0.05 was considered statistically significant.GWhisker‐box plot showing log_2_ fold change distributions of quantified proteins by LC–MS/MS in HeLa *TMBIM5* ^−/−^ and wildtype (WT) cells (n = 5 independent experiments), which were identified in specific OXPHOS complexes I‐IV. AP = all cell proteome (8,962 proteins), M = MitoCarta 3.0 (932 proteins), CI = complex I (40 proteins), CII = complex II (4 proteins), CIII = complex III (8 proteins), CIV = complex IV (14 proteins). Boxes borders indicate the 25% and 75% quantiles and outliers are indicated by a + sign (greater distance than 1.5 * inter quantile range).HBar graph displaying log2 fold changes between transcript (mRNA) and protein changes for subunits of the NADH dehydrogenase complex I.IScatter plot comparing the turnover rate constants in WT and *TMBIM5* ^−/−^ HeLa cells. Stable isotope incorporation upon SILAC was monitored for six biological replicates at seven time points (0, 2, 4, 6, 8, 12, 24 h). The incorporation rate was calculated and a first‐order kinetic model was fitted to each peptide, which allows for the estimation of the turnover rate constant k [1/h]. For a protein group, the mean peptide turnover constant was calculated. Subset of MitoCarta 3.0 assigned proteins are shown. Significantly different rate constants were identified using a two‐sided *t*‐test (*P*‐value < 0.05). Complex I subunits are highlighted in red. See also Dataset EV5.JCartoon of respiratory complex I highlighting the subunits affected by the deletion of *TMBIM5*. Orange subunits are part of N module (NDUFA2, NDUFA12, NDUFV2, NDUFS1). Green subunits are part of the Q module (NDUFA5, NDUFA9, NDUFS3, NDUFS7, NDUFS8, and the assembly factor NDUFAF4). The light blue subunit is part of the P module (NDUFB11). Data information: Datasets [Link], [Link], [Link], [Link].

**Figure EV3:**
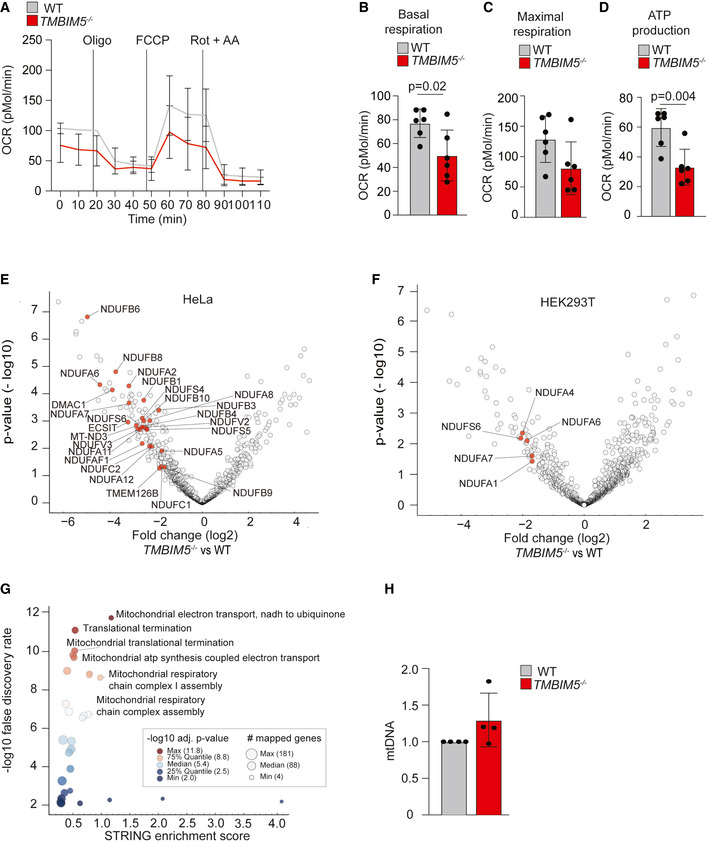
TMBIM5 controls oxidative phosphorylation (Related to Fig 4) AOxygen consumption rate (OCR) of wildtype (WT) and *TMBIM5* ^−/−^ HEK293T cells in glucose media. Traces are mean ± SD of six independent experiments, each one run at least in triplicate. Labeled lines denotes injections of oligomycin (Oligo, 2 μM), FCCP (0.5 μM), rotenone and antimycin A (Rot + AA; both 0.5 μM).BBasal respiration calculated from OCR experiment of WT and *TMBIM5* ^−/−^ HEK293T cells in (A). Mean ± SD; two‐tailed *t*‐test. A *P*‐value of < 0.05 was considered statistically significant.CMaximal respiration calculated from OCR experiment of WT and *TMBIM5* ^−/−^ HEK293T cells in (A). Mean ± SD; two‐tailed *t*‐test. A *P*‐value of < 0.05 was considered statistically significant.DATP production calculated from OCR experiments of WT and *TMBIM5* ^−/−^ HEK293T cells in (A). Mean ± SD; two‐tailed *t*‐test. A *P*‐value of < 0.05 was considered statistically significant.EVolcano plot of mitochondrial protein changes in *TMBIM5* ^−/−^ HeLa cells when compared to WT HeLa cells. Significantly enriched mitochondrial complex I proteins at an FDR cutoff of 0.05 are colored in red. *n* = 6 independent experiments. *P*‐values were calculated from a two‐sided *t*‐test followed by a permutation‐based FDR controlling to 0.05. See also Dataset EV2.FVolcano plot of mitochondrial protein changes in *TMBIM5* ^−/−^ HEK293T cells when compared to WT HEK293T cells. Significantly enriched mitochondrial complex I proteins at an FDR cutoff of 0.05 are colored in red. *n* = 5 independent experiments. *P*‐values were calculated from a two‐sided *t*‐test followed by a permutation‐based FDR controlling to 0.05. See also Dataset EV3.GScatter plot comparing the STRING enrichment score versus the −log10 transformed false discovery rate (color encoded). The number of genes mapped to the enriched GOBP term is encoded by size.HmtDNA levels from HeLa cells assessed by qPCR amplification of mitochondrial CYTB (*n* = 4 independent experiments, mean ± SD).

We determined in further experiments how TMBIM5 affects mitochondrial bioenergetics. *TMBIM5* ^−/−^ cells showed decreased basal and maximal oxygen consumption rates and a reduced spare respiratory capacity (Figs 4A–C and EV3A–C), as has been observed in TMBIM5‐deficient HAP1 cells (Seitaj *et al*, 2020). The production of ATP was also diminished in these cells (Figs 4D and EV3D). Reduced respiration was accompanied by an increased ROS production in *TMBIM5* ^−/−^ cells (Fig 4E). When analyzing specific enzymatic activities of individual respiratory chain complexes in isolated mitochondria, we observed a reduced complex I activity, while the activity of the other respiratory complexes was not significantly affected (Fig 4F). These experiments suggest that a reduced specific activity of complex I limits mitochondrial respiration in *TMBIM5* ^−/−^ mitochondria.

To corroborate these findings, we analyzed the mitochondrial proteome in *TMBIM5* ^−/−^ cells by mass spectrometry (Fig EV3E and F, Datasets EV2 and EV3). Deletion of *TMBIM5* was accompanied by broad alterations in the mitochondrial proteome (Fig EV3E and F, Datasets EV2 and EV3). STRING Gene Ontology enrichment analysis revealed that respiratory chain complexes, in particular complex I, were predominantly affected (Fig EV3G). We therefore compared the steady‐state levels of all detected mitochondrial proteins and specifically those of subunits of the different respiratory complexes in wildtype and *TMBIM5* ^−/−^ mitochondria (Fig 4G). Loss of TMBIM5 did not grossly alter the mitochondrial mass (Fig 4G) or mtDNA levels (Fig EV3H). However, subunits of respiratory complex I were significantly decreased in the absence of TMBIM5, in line with its reduced specific activity (Fig 4F). Subunits of other respiratory chain complexes were present at wildtype levels in *TMBIM5* ^−/−^ mitochondria (Fig 4G). We therefore conclude that the loss of TMBIM5 has profound effects on the mitochondrial proteome and leads to reduced levels of respiratory complex I.

**Figure EV4:**
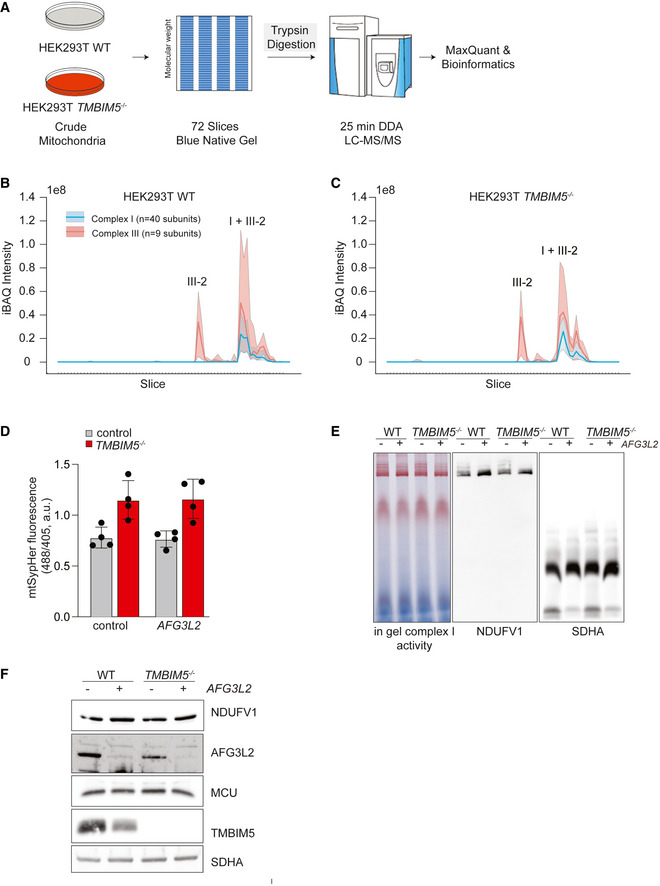
Loss of TMBIM5 promotes proteolysis by AFG3L2 (Related to Fig 5) AWorkflow of the complexome profiling experiment. Mitochondria were isolated from wildtype (WT) and *TMBIM5* ^−/−^ HEK293T cells and protein complexes were fractionated using Blue‐native PAGE (BN–PAGE). Each lane was cut into 72 slices of equal size and digested using trypsin followed by LC–MS/MS analysis.BProfile plot showing the iBAQ intensity of MitoCarta 3.0 complex I and complex III subunits across the measured gel slices (fractions) of a BN–PAGE upon fractionation of a mitochondrial fraction of HEK293T cells. Line represents the median of all individual proteins. The area indicates the interquantile range.CSimilar to (B), but for mitochondria from HEK293T *TMBIM5* ^−/−^ cells.DMonitoring mitochondrial matrix pH using the synthetic pH sensor mtSypHer expressed in WT and *TMBIM5* ^−/−^ cells depleted of AFG3L2 when indicated.Four independent experiments; mean ± SD.EBN–PAGE analysis of mitochondria isolated from WT and *TMBIM5* ^−/−^ HEK293T cells. Left panel, in‐gel activity assay for respiratory complex I after fractionation by BN–PAGE of WT and *TMBIM5* ^−/−^ mitochondria depleted of AFG3L2 as indicated. Middle and right panel, immunoblot analysis of the BN–PAGE using antibodies directed against NDUFV1 and SDHA.FSDS–PAGE analysis of the mitochondrial fraction analyzed in D. Source data are available online for this figure.

To assess whether TMBIM5 affects the assembly of complex I, we performed complexome profiling after fractionating wildtype and *TMBIM5* ^−/−^ mitochondria by BN–PAGE (Fig EV4A) (Heide *et al*, 2012). We identified individual proteins by LC–MS/MS and quantified signaling profiles of 40 subunits of complex I and nine subunits of complex III (defined by MitoCarta 3.0). The loss of TMBIM5 did not impair the assembly of complex I subunits into respiratory chain supercomplexes formed by complex I and III (Fig EV4B and C; Dataset EV4), demonstrating that TMBIM5 is not required for complex I assembly.

The decreased steady‐state level of complex I subunits in *TMBIM5* cells does not reflect the reduced expression of these proteins (Fig 4H; Appendix Fig S1A), suggesting that TMBIM5 affects their stability. We therefore monitored the protein turnover upon stable isotope labeling with amino acids in cell culture (SILAC) (Ong *et al*, 2002) of wildtype and *TMBIM5* ^−/−^ cells, which proliferated at similar rates (Appendix Fig S1B and C). After labeling cells with ^13^C‐ and ^15^N‐labeled arginine and lysine, cells were further incubated in the presence of light isotopologues and the cellular proteome was determined at seven timepoints within 48 h in six biological replicates by mass spectrometry using a first‐order model kinetic (Appendix Fig S1B). We identified significantly altered protein turnover rates for 987 proteins, including 348 mitochondrial proteins (Appendix Fig S1D). A broad effect on nonmitochondrial proteins can be expected Since the loss of TMBIM5 severely impairs mitochondrial function. Eighty‐nine mitochondrial proteins were significantly (*P* < 0.01 by two‐sided *t*‐test) faster turned over in *TMBIM5* ^−/−^ cells relative to wildtype cells (Dataset EV5). The vast majority of these proteins are localized at the mitochondrial IM or matrix (131 out of 140). They include components of mitochondrial ribosomes and of the mitochondrial gene expression apparatus, and various subunits of respiratory complex I, which were among the most significantly destabilized proteins (Fig 4I and J; Dataset EV5).

We conclude from these experiments that TMBIM5 broadly affects mitochondrial protein stability. Loss of TMBIM5 increases the turnover of a large number of mitochondrial proteins, including complex I subunits, leading to decreased complex I activity.

### TMBIM5 deletion promotes proteolysis by AFG3L2

Different turnover rates have been observed for the 44 subunits of complex I (Szczepanowska & Trifunovic, 2021). Subunits of the matrix‐exposed N module are generally more rapidly degraded than membrane‐bound core subunits and exchanged by newly synthesized subunits, likely to repair oxidative damage (Pryde *et al*, 2016; Szczepanowska *et al*, 2020). The degradation of N module subunits (NDUFS1, NDUFV1, and NDUFV2) is mediated by matrix‐localized LON and CLPP proteases. The loss of TMBIM5 led to the proteolytic breakdown of 4 out of 5 identified subunits of the N module (NDUFS1, NDUFA2, NDUFA12, and NDUFV2), but we also observed increased proteolysis of 6 out of 10 identified subunits of the Q module (NDUFA5, NDUFA9, NDUFS3, NDUFS7, NDUFS8, and the assembly factor NDUFAF4) and 1 out of 22 identified subunits of the P module in the IM (NDUFB11) (Fig 4I and J).

**Figure 5:**
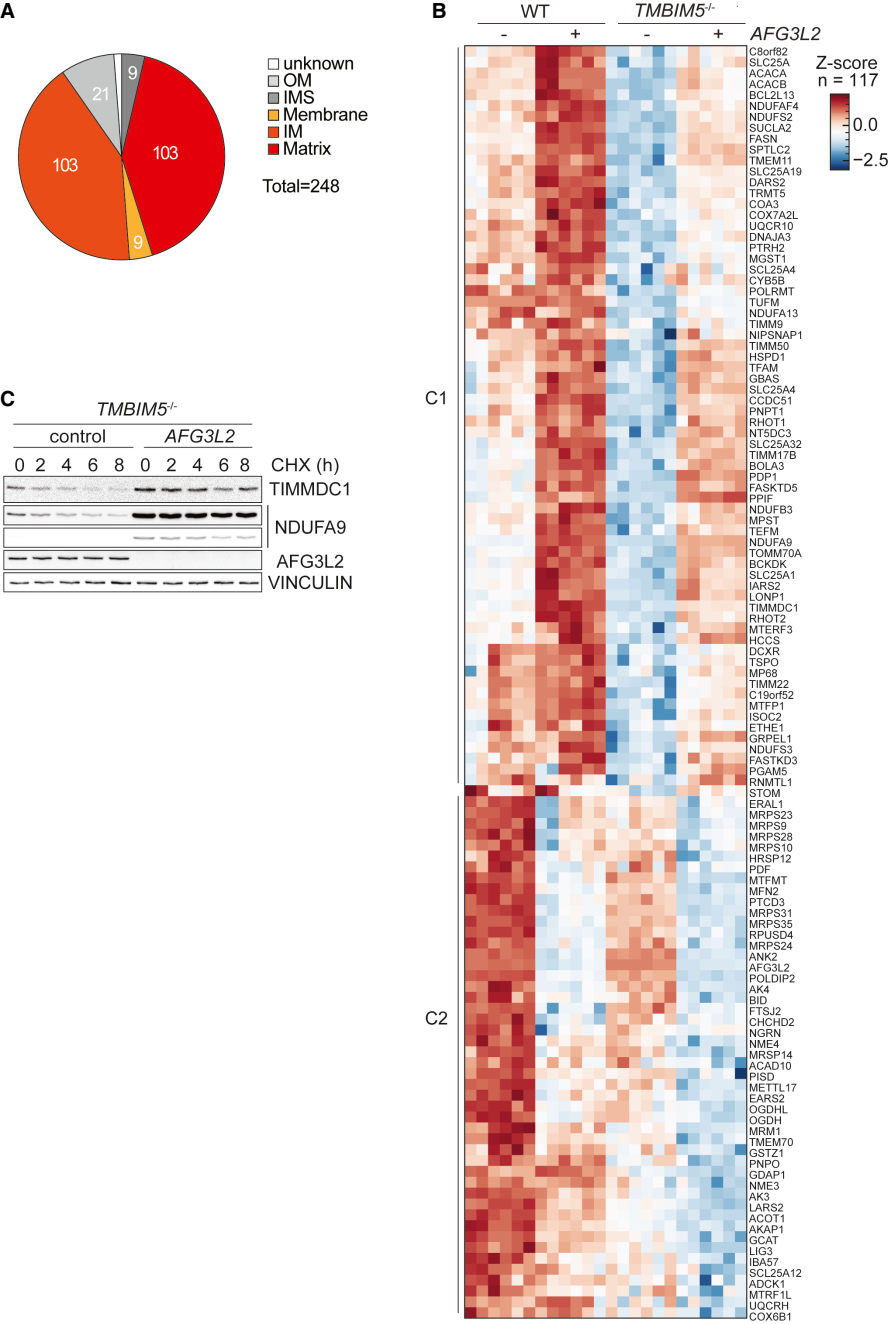
Loss of TMBIM5 promotes proteolysis by AFG3L2 APie chart showing the localization of proteins accumulating at decreased levels in *TMBIM5* ^−/−^ HeLa cells. Localization according to MitoCarta 3.0. OM, outer membrane; IMS, intermembrane space; IM, inner membrane.BAlterations in mitochondrial proteome in WT and *TMBIM5* ^−/−^ HeLa cells upon depletion of AFG3L2. WT and *TMBIM5* ^−/−^ HeLa cells were transfected with scrambled siRNA (control) or siRNA targeting *AFG3L2* for 48 h. Proteins, which were downregulated in the absence of TMBIM5 and whose steady‐state levels were altered upon depletion of AFG3L2, were identified using a two‐way ANOVA. An interaction *P*‐value of 0.01 was used as a cutoff. Log2‐transformed LFQ intensities of MitoCarta 3.0 proteins were *Z*‐Score normalized and visualized in hierarchical cluster analysis (Euclidean distance, complete method). The dendrogram is omitted. Proteins accumulating upon depletion of AFG3L2 (cluster 1, C1) and proteins whose steady‐state levels are decreased upon depletion of AFG3L2 (cluster 2, C2) are shown. See also Dataset EV6.CRepresentative immunoblot of *TMBIM5* ^−/−^ HeLa cells transfected with scrambled siRNA (control) or siRNA targeting *AFG3L2* for 48 h. Samples were treated with the protein synthesis inhibitor cycloheximide (CHX; 10 μg/ml) and collected at the indicated time points (*n* = 3 independent experiments). Data information: See also Dataset EV6. Source data are available online for this figure.

Since we have identified TMBIM5 as a constituent of AFG3L2‐containing complexes (Fig 1A), we examined a possible involvement of AFG3L2 in the degradation of complex I subunits in TMBIM5‐deficient mitochondria. An unbiased analysis of the proteolysis of OXPHOS subunits is hampered by the known function of AFG3L2 for mitochondrial ribosome biogenesis (Almajan *et al*, 2012). Rather than deleting *AFG3L2*, we therefore acutely depleted AFG3L2 from wildtype and *TMBIM5* ^−/−^ mitochondria to limit effects on mitochondrial protein synthesis and determined the mitochondrial proteome by mass spectrometry. Among the 248 proteins whose steady‐state levels were decreased in mitochondria lacking TMBIM5 (Fig EV3E), 215 proteins are localized to the IM or matrix space and therefore may include substrates of AFG3L2 (Fig 5A). Consistently, our unbiased proteomic approach revealed that steady‐state levels of 117 of the 215 proteins were significantly altered upon depletion of AFG3L2 in *TMBIM5* ^−/−^ cells (Fig 5B; Dataset EV6). Two protein classes can be distinguished: proteins that accumulate in the absence of AFG3L2 (cluster 1; 69 proteins) and proteins whose steady‐state levels further decrease upon downregulation of AFG3L2 (cluster 2; 48 proteins). Whereas cluster 2 proteins are likely only indirectly affected by AFG3L2, cluster 1 proteins represent candidate substrates of AFG3L2. Cluster 1 proteins also accumulate upon depletion of AFG3L2 in wildtype cells indicating AFG3L2‐mediated turnover, which appears to be accelerated upon loss of TMBIM5 and mitochondrial hyperpolarization (Fig 5B). Notably, out of 89 proteins that were more rapidly degraded in *TMBIM5* ^−/−^ cells (Fig 4I), 32 proteins, including NDUFA9 and NDUFS3, accumulated upon depletion of AFG3L2, indicating that AFG3L2 mediates their proteolytic breakdown. Depletion of AFG3L2 did not affect matrix alkalinization in *TMBIM5* ^−/−^ mitochondria excluding indirect effects (Fig EV4D).

Immunoblot analysis of cellular fractions after inhibition of cytosolic protein synthesis confirmed the AFG3L2‐dependent degradation of NDUFA9 and of the complex I assembly factor TIMMDC1 that was previously described as an AFG3L2 substrate (Wang *et al*, 2021) (Fig 5C). BN–PAGE analysis combined with complex I activity staining revealed the formation of assembled and active complexes I in *TMBIM5* ^−/−^ mitochondria and a moderate increase in complex formation upon depletion of AFG3L2 (Fig EV4E and F).

We conclude from these experiments that the loss of TMBIM5 causes broad reshaping of the mitochondrial proteome and promotes the AFG3L2‐mediated degradation of IM and matrix proteins, including subunits of respiratory complex I.

### Hyperpolarization induces TMBIM5 degradation

**Figure 6:**
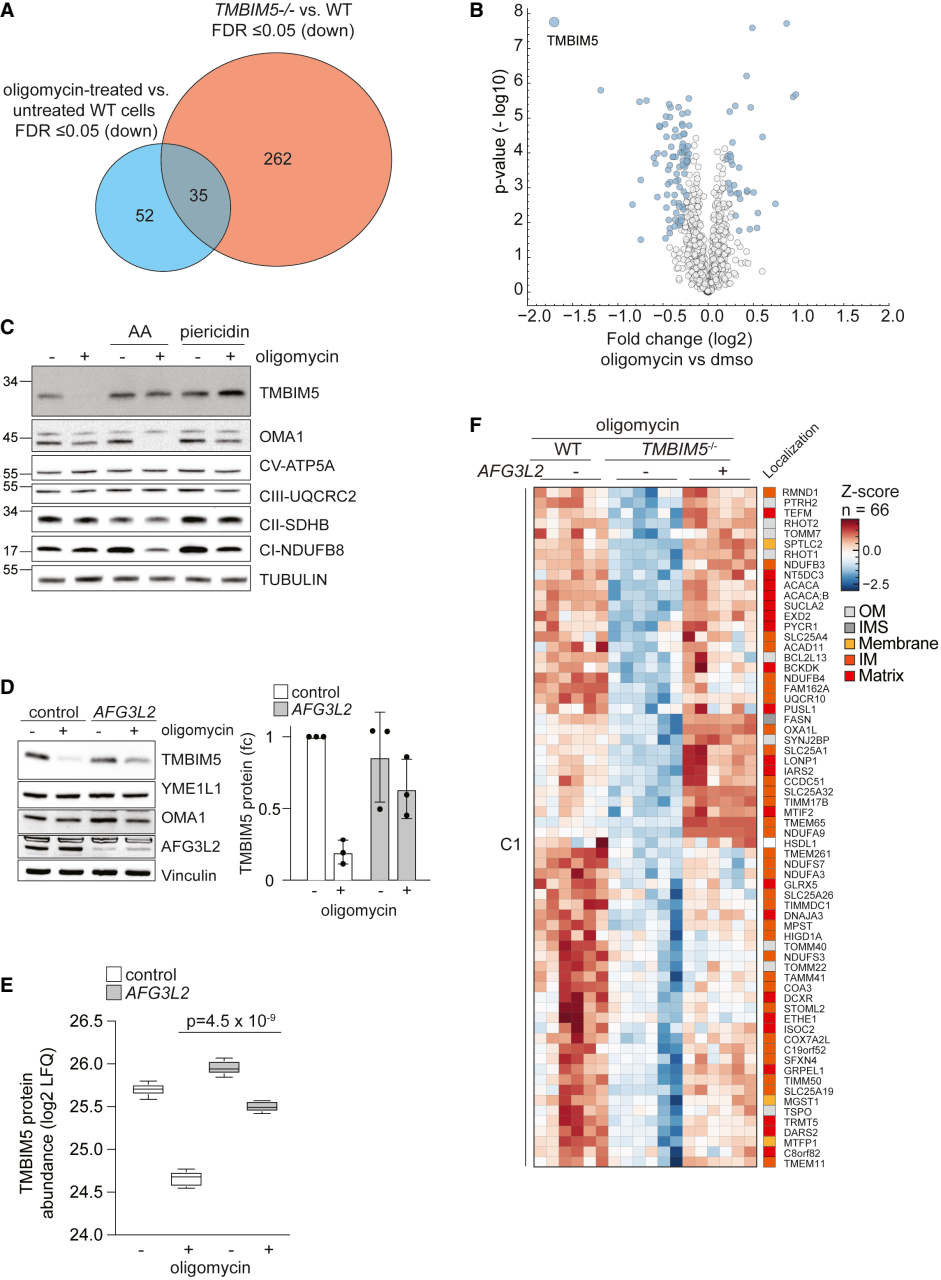
Hyperpolarization induces degradation of TMBIM5 allowing AFG3L2‐mediated remodeling of the mitochondrial proteome AVenn diagram of significantly downregulated proteins (FDR ≤ 0.05) in oligomycin‐treated wildtype (WT) HeLa cells (0.01% DMSO vs oligomycin 10 μM for 16 h; blue) and in *TMBIM5* ^−/−^ HeLa cells (WT vs *TMBIM5* ^−/−^ HeLa cells; red). See also Dataset EV7.BVolcano plot of mitochondrial protein (MitoCarta 3.0) changes in WT HeLa cells treated with oligomycin (10 μM for 16 h) when compared to untreated cells. Before MitoCarta 3.0‐filtering, significantly altered proteins were identified using a two‐sided *t*‐test followed by permutation‐based FDR estimation to 0.05 and are colored in blue (*n* = 5 independent experiments). See also Dataset EV7.CRepresentative immunoblot of WT HeLa cells treated with the indicated drugs for 16 h. Antimycin A (AA; 10 μM), piericidin A (10 μM) and oligomycin (10 μM). Quantification is shown in Fig EV5B (*n* = 7 independent experiments).DRepresentative immunoblot of WT HeLa cells transfected with scrambled siRNA (control) and siRNA targeting *AFG3L2* for 48 h, treated when indicated with oligomycin (10 μM) for 16 h. Quantification of TMBIM5 levels is shown on the right panel. TMBIM5 levels in untreated WT cells were set to 1 (*n* = 3 independent experiments; mean ± SD).ETMBIM5 abundance determined by mass spectrometry in WT HeLa cells treated with oligomycin (10 μM; 16 h) and depleted of AFG3L2 when indicated. *n* = 5 independent experiments. *P*‐value was calculated using a two‐sided unpaired *t*‐test. The central band of each box is the median value, and the box defines the 25^th^ (lower) and 75^th^ (higher) quantile. The whiskers represent the minimum and maximum value in the data excluding outliers (> 1.5 * inter quantile range distance to median—no outliers found here).FZoom in to selected clusters of hierarchical clustering of *Z*‐Score normalized log2 LFQ intensities. Proteins that were significantly (FDR < 0.05) accumulated between HeLa WT and *TMBIM5* ^−/−^ cells after oligomycin treatment (10 μM) for 16 h were clustered using Euclidean distance and the complete method on protein features. The figure shows identified cluster C4 and C5 with gene names and MitoCarta 3.0 localization annotations. The row dendrogram is omitted. See also Dataset EV6. Data information: See also Datasets EV6 and EV7. Source data are available online for this figure.

**Figure EV5:**
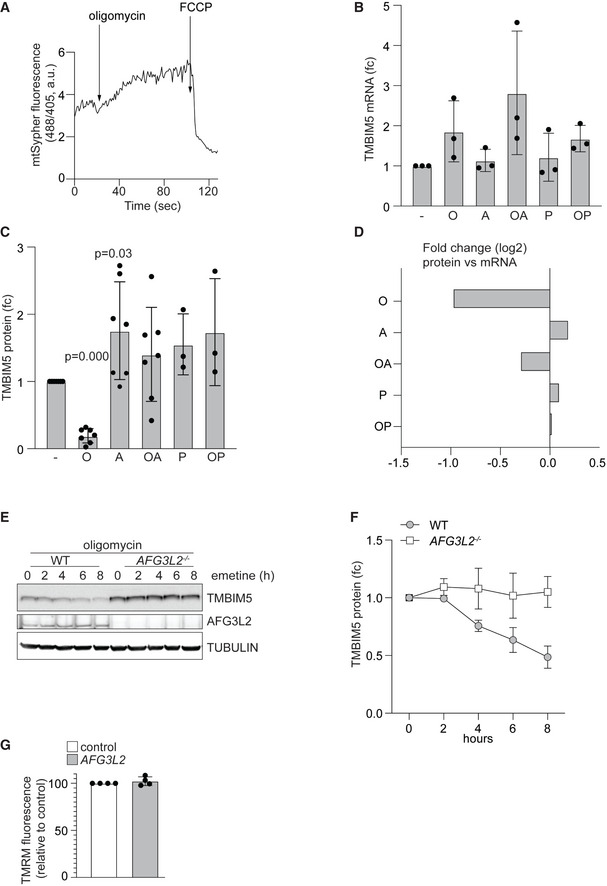
Hyperpolarization induces degradation of TMBIM5 allowing AFG3L2‐mediated remodeling of the mitochondrial proteome (Related to Fig 6) ARepresentative mtSypHer traces of HeLa cells treated with oligomycin (10 μM) or FCCP (10 μM) at the indicated timepoints. Ratios of the absorbance at 488 nm and 405 nm are shown in arbitrary units (a.u.).BQuantification of *TMBIM5* mRNA monitored by RT–qPCR of HeLa cells treated as in Fig 6C. *n* = 3 independent experiments; mean ± SD.CQuantification of Fig 6C. WT HeLa cells treated with the indicated drugs for 16 h. antimycin A (A; 10 μM), piericidin (P; 10 μM), and oligomycin (O; 10 μM). *n* = 7 independent experiments; mean ± SD.DBar graph displaying log2 fold changes between transcript (mRNA) (Fig EV5C) and protein (Fig EV5B) changes for TMBIM5 in cells treated as indicated.ERepresentative immunoblot of WT HeLa cells transfected of wildtype (WT) and *AFG3L2* ^*−/−*^ cells. Samples were treated with the protein synthesis inhibitor emetine (10 μg/ml) and oligomycin (10 μM) and analyzed at the indicated time points (*n* = 3 independent experiments).FQuantification of experiments shown in (E). Data are shown as mean ± SD.GMitochondrial membrane potential was monitored with TMRM in WT HeLa cells transiently transfected with scrambled siRNA (control) and siRNA targeting *AFG3L2* for 48 h. Fluorescent intensity was calculated relative to the value in the presence of CCCP (15 μM). Values are expressed as mean ± SD relative to control. Each measurement was performed in triplicate from four different preparations. Source data are available online for this figure.

It is conceivable that mitochondrial hyperpolarization in the absence of TMBIM5 triggers proteolysis. To examine this possibility, we induced mitochondrial hyperpolarization by inhibiting the F_1_F_O_ ATP synthase with oligomycin in wildtype cells and monitored effects on the mitochondrial proteome by mass spectrometry (Figs 6A and B, and EV5A; Dataset EV7). Surprisingly, only 87 mitochondrial proteins were significantly downregulated upon oligomycin treatment, i.e., significantly less proteins than in *TMBIM5* ^−/−^ cells (Fig 6A; Dataset EV7). Only 35 of the proteins were affected by both oligomycin treatment and deletion of *TMBIM5* (Fig 6A; Dataset EV7). Thus, mitochondrial hyperpolarization is not sufficient to explain the broad effect of TMBIM5 on the mitochondrial proteome.

We noticed that among all detected mitochondrial proteins TMBIM5 was the strongest decreased protein upon mitochondrial hyperpolarization (Fig 6B; Dataset EV7), although TMBIM5 transcript levels were not significantly altered under these conditions (Fig EV5B). We reasoned that pH‐dependent conformational changes in the presence of oligomycin may destabilize TMBIM5, as it has been suggested for other TMBIM family members (Saraiva *et al*, 2013). We therefore incubated oligomycin‐treated cells additionally with other OXPHOS inhibitors, which limit H^+^ transport and thus prevent matrix alkalinization and hyperpolarization of the IM (Fig 6C). Inhibition of respiratory complex I with piericidin A or of respiratory complex III with antimycin A indeed stabilized TMBIM5 in cells treated with oligomycin but did not affect TMBIM5 levels in untreated cells (Figs 6C and EV5B–D). These results reveal that hyperpolarization triggers proteolysis of TMBIM5, which is abolished upon depolarization of mitochondria.

Degradation of TMBIM5 in hyperpolarized mitochondria is at least in part mediated by AFG3L2. Downregulation of AFG3L2 slowed degradation and stabilized TMBIM5 in the presence of oligomycin (Fig 6D). Notably, AFG3L2 depletion did not depolarize mitochondria under these conditions, excluding indirect effects on TMBIM5 stability (Fig EV5G). Moreover, TIMBIM5 stably accumulated in *AFG3L2* ^−/−^ cells which were incubated in the presence of the translational inhibitor emetine (Fig EV5E and F). Similarly, our proteomic analysis revealed the accumulation of TMBIM5 in oligomycin‐treated cells upon downregulation of AFG3L2 (Fig 6E). Although not excluding the involvement of other peptidases, these experiments demonstrate that AFG3L2 contributes to the degradation of TMBIM5 upon persistent hyperpolarization of mitochondria.

### Hyperpolarization and TMBIM5 degradation allow remodeling of the mitochondrial proteome by AFG3L2

These experiments identify TMBIM5 both as a constituent of AFG3L2‐containing complexes and as substrate of AFG3L2, which mediates TMBIM5 turnover upon mitochondrial hyperpolarization. This is reminiscent of *E. coli* YccA, a distant homolog of the TMBIM family, which shares 12.5% sequence identity with TMBIM5. YccA is degraded by FtsH, the bacterial homolog of AFG3L2, while its binding to FtsH inhibits proteolysis of other FtsH substrates (Kihara *et al*, 1998; van Stelten *et al*, 2009). Assuming functional conservation, we hypothesized that TMBIM5 binding inhibits AFG3L2 activity, while its degradation in hyperpolarized mitochondria allows broad reshaping of the mitochondrial proteome by AFG3L2 mediated proteolysis. The degradation of TMBIM5, acting as a competitive inhibitor, may restrict AFG3L2 activity upon acute hyperpolarization and may explain the limited effect of oligomycin on the mitochondrial proteome. However, progressive TMBIM5 degradation upon persistent hyperpolarization is expected to increasingly release AFG3L2 activity towards other substrates and drive the proteolytic breakdown of mitochondrial proteins.

To examine this hypothesis, we analyzed how depletion of AFG3L2 affected mitochondrial proteins in oligomycin‐treated *TMBIM5* ^−/−^ cells (Fig 6F). As expected, we observed broad and similar changes in the mitochondrial proteome in *TMBIM5* ^−/−^ cells, regardless of oligomycin treatment of the cells. We identified 66 proteins, whose steady‐state levels were significantly decreased in hyperpolarized *TMBIM5* ^−/−^ cells when compared to wildtype cells, but which accumulated upon depletion of AFG3L2 (Fig 6F; Dataset EV6). Fifty‐six of these proteins, including eight subunits and assembly factors of respiratory complex I (NDUFA3, NDUFA9, NDUFS3, NDUFS7, NDUFB3, NDUFB4, TMEM261, and TIMMDC1), are localized in the IM or matrix of mitochondria and therefore likely represent AFG3L2 substrates in hyperpolarized mitochondria. These data demonstrate increased proteolysis by AFG3L2 in hyperpolarized mitochondria lacking TMBIM5, substantiating the regulation of AFG3L2 by TMBIM5 and the proton gradient.

## Discussion

**Figure 7:**
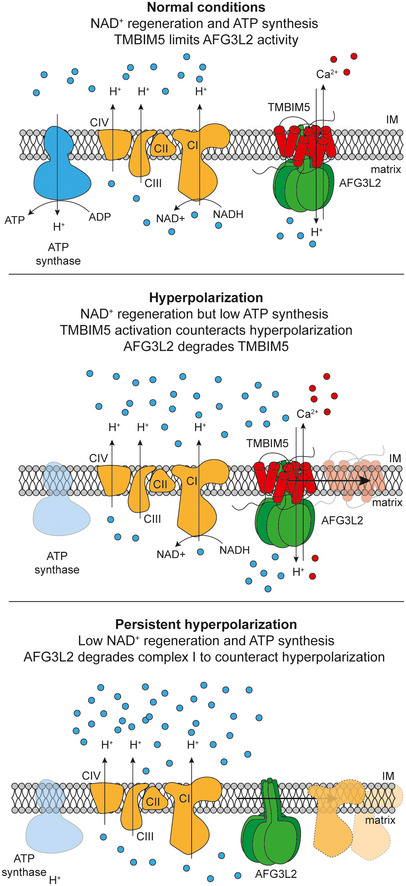
Model for the regulation of AFG3L2‐mediated proteolysis by TMBIM5 under different metabolic conditions Upper panel, TMBIM5 inhibits AFG3L2 and, acting as a Ca^2+^/H^+^ exchanger, limits the formation of the mitochondrial H^+^ gradient. Middle panel, TMBIM5 counteracts acute hyperpolarization and limits ROS production, for instance, if the demand for mitochondrial ATP is low. Moreover, TMBIM5 inhibits AFG3L2‐mediated proteolysis allowing electron flow through complex I and NAD^+^ regeneration. Lower panel, persistent mitochondrial hyperpolarization induces the proteolytic breakdown of TMBIM5 and releases AFG3L2 activity, which reshapes the mitochondrial proteome and degrades respiratory complex I subunits to prevent excessive ROS production.

We have identified TMBIM5 as a mitochondrial Ca^2+^/H^+^ exchanger and constituent of AFG3L2‐containing complexes, which inhibits the m‐AAA protease AFG3L2 and therefore coordinates mitochondrial protein turnover with the energetic status of mitochondria (Fig 7). TMBIM5 allows Ca^2+^ efflux from mitochondria counteracting Ca^2+^ overload and apoptosis. Moreover, TMBIM5 activity limits the formation of the H^+^ gradient across the IM and inhibits proteolysis by the m‐AAA protease. Persistent mitochondrial hyperpolarization induces the degradation of TMBIM5 and activation of the m‐AAA protease associated with broad remodeling of the mitochondrial proteome. The resulting degradation of complex I subunits limits ROS production in hyperpolarized mitochondria and contributes to the rebalancing of the H^+^ gradient.

We used cellular and *in vitro* reconstitution experiments to demonstrate that TMBIM5 mediates Ca^2+^ efflux across the IM, which is accompanied by H^+^ transport into the mitochondrial matrix. Thus, TMBIM5 appears to exert Ca^2+^/H^+^ exchange activity, while other members of the TMBIM family act as pH‐dependent Ca^2+^ channels in membranes lacking a stable H^+^ gradient (Bultynck *et al*, 2014; Guo *et al*, 2019; Li *et al*, 2020). Similar to the Na^+^/Ca^2+^ exchanger NCLX (Palty *et al*, 2010; Katoshevski *et al*, 2021), TMBIM5 allows the efflux of Ca^2+^ from mitochondria, raising the question why different Ca^2+^ efflux routes have evolved. Although TMBIM5 is ubiquitously expressed in mice, tissue‐specific demands may exist. Moreover, both TMBIM5 and NCLX affect OXPHOS activity but exchange Ca^2+^ against different cations. Na^+^ influx through NCLX directly modulates complex II and III activity by affecting membrane fluidity (Hernansanz‐Agustin *et al*, 2020). We demonstrate here that TMBIM5 preserves respiration regulating the formation of the H^+^ gradient across the IM. Accordingly, loss of TMBIM5 leads to mitochondrial hyperpolarization and impairs OXPHOS activities. We propose that the coupling of cycles of Ca^2+^ influx via MCU and Ca^2+^ efflux via TMBIM5 allows for tuning the H^+^ gradient across the IM independent of the F_1_F_O_‐ATP synthase. This pathway may be of relevance when the demand for NAD^+^ exceeds that for mitochondrial ATP, as observed during aerobic glycolysis (Luengo *et al*, 2021). Cell growth does not depend on mitochondrial ATP in glycolytic cells, but NAD^+^ regeneration at respiratory complex I maintains the cellular redox homeostasis and the activity of numerous NAD^+^‐dependent enzymes (Covarrubias *et al*, 2021). Under conditions of limited mitochondrial ATP production, dissipation of the H^+^ gradient by TMBIM5 counteracts hyperpolarization (Fig 7). TMBIM5 thereby preserves H^+^ transport by respiratory complexes and facilitates NAD^+^ regeneration at complex I, while at the same time limiting ROS production. The Ca^2+^/H^+^ exchange activity of TMBIM5 therefore allows to utilize the proton gradient for NAD^+^ regeneration independent of the F_1_F_O_‐ATP synthase.

This metabolic role of TMBIM5 is supported by its second activity as an inhibitor of AFG3L2. Inhibition of the m‐AAA protease prevents the premature degradation of unassembled or damaged respiratory chain subunits and therefore maintains respiration. We identify TMBIM5 as a substrate of AFG3L2, whose proteolysis inhibits the degradation of other AFG3L2 substrates upon acute hyperpolarization. Only persistent hyperpolarization leads to the complete loss of TMBIM5 and the release of m‐AAA protease activity (Fig 7). It is conceivable that hyperpolarization induces conformational changes in TMBIM5, reminiscent of other TMBIM family members. Physiological pH changes were shown to trigger TMBIM4 oligomerization regulating its anti‐apoptotic function (Saraiva *et al*, 2013). In the absence of TMBIM5, the m‐AAA protease broadly reshapes the mitochondrial proteome. Our unbiased proteomic approaches highlight the central role of the m‐AAA protease in mitochondrial proteostasis. Degradation of subunits of complex I, which are among the most affected proteins in the absence of TMBIM5, limits respiration and ROS production. Previous studies have already revealed rapid degradation of N module subunits of complex I upon mitochondrial damage and established the involvement of the matrix‐localized LON and CLPP peptidases (Pryde *et al*, 2016; Szczepanowska *et al*, 2020). Our experiments are consistent with and complement these findings and demonstrate that various mitochondrial quality control peptidases are involved in the turnover of complex I subunits. It is an attractive possibility that the topology of the subunits within complex I and the stability of sub‐complexes determine the involvement of a protease in their proteolytic breakdown. Notably, increased AFG3L2‐mediated proteolysis does not solely explain the broad alterations in the mitochondrial proteome in the absence of TMBIM5. It is conceivable that increased AFG3L2‐mediated proteolysis upon loss of TMBIM5 indirectly triggers proteolysis by other mitochondrial peptidases.

Similar to the regulation of the m‐AAA protease by TMBIM5, *E. coli* YccA distantly related to TMBIM5 inhibits the bacterial homolog of the m‐AAA protease FtsH (Kihara *et al*, 1998; van Stelten *et al*, 2009). Although an ion transport activity of YccA has not been demonstrated, YccA shares a limited sequence identity with the pH‐sensitive domain of TMBIM proteins, suggesting conserved regulation of FtsH‐like proteases from bacteria to human. Notably, a homolog of mitochondrial TMBIM5 is not present in the yeast *S. cerevisiae*, which also lacks MCU as the major Ca^2+^ uptake route into mitochondria and respiratory complex I, pointing to a co‐evolution of the Ca^2+^ dependent regulation of proteolytic activity with these complexes. Regardless, our results identify the m‐AAA protease as a central hub shaping Ca^2+^ cycling into mitochondria. The m‐AAA protease limits Ca^2+^ efflux from hyperpolarized mitochondria by degrading TMBIM5 and, at the same time, prevents mitochondrial Ca^2+^ overload by the proteolytic breakdown of excess EMRE subunits, which ensures the formation of gated MCU‐EMRE channels only (Konig *et al*, 2016; Tsai *et al*, 2017). It will be of interest to define the role of these regulatory circuits in neurodegenerative disorders associated with mutations in m‐AAA protease subunits. Considering the prominent role of mitochondrial Ca^2+^ homeostasis for mitochondrial metabolism, cell signaling, and cell death pathways (Garbincius & Elrod, 2021), TMBIM5 acting as a Ca^2+^/H^+^ exchanger and inhibitor of the m‐AAA protease is likely of central importance to both in healthy and pathophysiological conditions.

## Material and Methods

### Reagents and tools

**Table:** 

Reagent or resource	Source	Identifier
**Antibodies**		
PARP	ThermoFisher	PA‐951
TMBIM5	Proteintech	16296‐1‐AP
VINCULIN	Cell Signalling	4650 or 13901
MCU	Sigma	HPA016480
Cytochrome *c* (IF)	ThermoFisher	33‐8200
TOMM20 (IF)	SIGMA	WH0009804M1
PHB1	NeoMarkers	RB‐292
AFG3L2	SIGMA	HPA004480
ATPVa	Abcam	Ab14748
TIMMDC1	Abcam	Ab171978
NDUFA9	Invitrogen	459100
OMA1	Santa Cruz	SC‐515788
Total oxphos rodent	Abcam	Ab110413
YME1L1	Proteintech	11510‐1‐AP
Tubulin	Sigma	T6074
**Chemicals, peptides, and recombinant proteins**		
Oligomycin	Sigma	Cat# O4876; CAS# 1404‐19‐9
Antimycin A	Sigma	Cat# A8674; CAS# 1397‐94‐0
Nigericin	Sigma	Cat# N7143; CAS# 28643‐80‐3
Piercidin	Chemcruz	Cat# SC‐202287; CAS# 2738‐64‐9
Cycloheximide	Sigma	Cat# C4859; CAS# 66‐81‐9
Rotenone	Sigma	Cat# R8875; CAS# 83‐79‐4
CCCP	Sigma	Cat# C2759; CAS# 55‐60‐2
Ionomycin	Sigma	Cat# I3909
Thapsigargin	Sigma	Cat# T9033; CAS# 67526‐95‐B
Staurosporine	Sigma	Cat# S6942
Actinomycin D	Sigma	Cat# A9415; CAS# 50‐76‐0
Venetoclax	Selleckchem	Cat# S8048; CAS# 1257044‐40‐8
A‐1155463	Selleckchem	Cat# S7800; CAS# 1235034‐55‐5
Ca2+ Green‐5N	Life technologies	Cat# C3737
Fura2	Invitrogen	Cat# F1221
TMRM	Invitrogen	Cat# T668
MitoSox	Invitrogen	Cat# M36008
Z‐VAD‐FMK	Bachem	Cat# 4026865; CAS# 220644‐02‐0
Hygromycin B	Roth	Cat# CP13.4
Tetracycline	Roth	Cat# 0237.1
**Critical commercial assays**		
MBPTrap HP columns	Cytiva	Cat# 28918780
NucView488 RedDot2 assay	Biotum	Cat# 30029
LysC endopeptidase	Sigma	Cat# T6567‐1MG
	Wako	Cat# 129‐02541
**Deposited data**		
Proteomics Data	This paper	PRIDE: PENDING
**Experimental models: Cell lines**		
HeLa TMBIM5^−/−^	This paper	N/A
Hek293T	Abcam	Cat# Ab255593
Hek293T TMBIM5^−/−^ (GHITM)	Abcam	Ab266479
Hek293TREX FLP	Invitrogen	Cat# R78007
Hek293TREX FLP AFG3L2 E408Q FLAG	This paper	N/A
**Oligonucleotides**		
siRNA targeting sequence: TMBIM5	Merck	SASI_HS01_00196210
siRNA targeting sequence: MCU	Merck	SASI_HS01_00234629_AS
siRNA targeting sequence: AFG3L2	Merck	SASI_HS01_0023‐1520
siRNA targeting sequence: NCLX	Merck	SASI_HS01_00236391_AS
Primers for sequencing HeLa TMBIM5‐/‐: FW	GCAGGTACCGGATTACAGGCATGAGCCACCGCAC	
Primers for sequencing HeLa TMBIM5‐/‐: RV	CGTGCGGCCGCGCTACAATTTTCTTTGAGCATCATG	
**Recombinant DNA**		
Plasmid: pcDNA5‐TO AFG3L2 E408Q‐FLAG	This paper	
Plasmid: px330cDNA5‐TO		Addgene #42230
Plasmid: mtRED‐GECO Plasmid: Sypher3mito		
Plasmid: pcDNA5‐TO TMBIM5 D294K D325K	This paper	
Plasmid: SypHer3s‐dmito	Ermakova *et al* (2018)	Addgene #108119
Plasmid: mtRed‐GECO	Wu *et al* (2013)	Addgene #46021
**Software and algorithms**		
ImageJ	Schneider *et al* (2012)	https://imagej.nih.gov/ij/
InstantClue	Nolte *et al* (2018)	http://www.instantclue.uni‐koeln.de/
GraphPad Prism		www.graphpad.com
MaxQuant	Cox and Mann (2008)	https://maxquant.org
Perseus	Tyanova *et al* (2016)	https://maxquant.org/perseus/
Spectronaut 15.5.211111.50606	Bruderer *et al* (2015)	www.biognosys.com
**Other**		
nLC 1200 Liquid Chromatography	Thermo Fisher Scientific	Cat# LC140
Buffer A: 0.1% (v/v) formic acid	Thermo Fisher Scientific	Cat# LS118‐1
Buffer B: 0.1% (v/v) formic acid in 80% acetonitrile	Thermo Fisher Scientific	Cat# LS122
PoroShell 120 EC‐C18 2.7‐μm beads	Agilent Technologies	Cat# 899999‐77
Exploris 480 Mass Spectrometer	Thermo Fisher Scientific	Cat# BRE725532

### Methods and protocols

#### Cell culture

If not otherwise indicated HeLa (ATCC, CCL‐2), HEK 293T (ATCC, CRL‐3216), and Flp‐In T‐REx 293 (Thermo Fisher) were maintained in DMEM‐GlutaMAX at 4.5 g × ml^−1^ D‐glucose (Gibco) supplied with sodium pyruvate, nonessential amino acids (both Gibco) and 10% fetal bovine serum (Sigma). Cells were seeded with equal densities before an experiment and regularly checked for mycoplasma contamination.

#### Method details

##### Reagents

Oligomycin (Cat# O4876), Antimycin A (Cat# A8674), nigericin (Cat# N7143), cycloheximide (Cat# C4859), rotenone (Cat# R8875), ionomycin (Cat# 13909), thapsigargin (Cat# T9033), staurosporine (Cat# S6942), Actinomycin D (Cat# A9415) were purchased from Sigma Aldrich. Piercidin (Cat# SC‐202287) was purchased from Chemcruz. Venetoclax (Cat# S8048) and A‐1155463 (S7800) were purchased from Selleckchem. Fura2 (Cat# F1200), TMRM (Cat# T668) and MitoSox (Cat# M36008) were purchased from Invitrogen. Calcium‐Green‐5N (Cat# C3737) was purchased from life technologies.

##### Antibodies

The following antibodies were used for immunoblotting: PARP (ThermoFisher, Cat# PA‐951; 1:1,000), TMBIM5 (Proteintech, Cat# 16296‐1‐AP; 1:2,000), VINCULIN (Cell signaling, Cat# 4650; 1:1,000), MCU (Sigma, Cat# HPA016480; 1:1,000), PHB1 (NeoMarkers, Cat# RB‐292; 1:500), AFG3L2 (Sigma, Cat# HPA004480; 1:5,000), ATPVa (Abcam, Cat# Ab14748; 1:5,000), TIMMDC1 (Abcam, Cat# Ab171978; 1:1,000), NDUFA9 (Invitrogen, Cat# 459100, 1:1,000), OMA1 (Santa Cruz, Cat# SC‐515788; 1:1,000), Total oxphos rodent (Abcam, Cat# Ab110413, 1:40,000), YME1L1 (Proteintech, Cat# 11510‐1‐AP; 1:2,000), Tubulin (Sigma, Cat# T6074; 1:5,000). The following antibodies were used for immunofluorescence: cytochrome c (ThermoFisher, 33‐8200; 1:500) and TOMM20 (Sigma, Cat# WH0009804M1; 1:500). Corresponding species‐specific HRP‐coupled antibodies were used for immunoblot (Biorad; Cat# 1706515 and Cat# 1706516) and fluorescently coupled secondary antibodies were used for immunofluorescence (Invitrogen Alexa Flour).

##### DNA plasmids

Complementary human cDNA encoding human TMBIM5 and AFG3L2‐FLAG was cloned into pcDNA5/FRT/TO (Invitrogen). AFG3L2 was mutated to AFG3L2(E408Q) and TMBIM5 to TMBIM5 D294L D325K via site‐directed mutagenesis.

##### Generation of stable expression cell lines

Flp‐In HEK293T‐REx cells were transfected with pcDNA5/FRT/TO AFG3L2^E408^FLAG. After transfection cells were selected with 100 μg/ml hygromycin (Roth, Cat# CP13.4) for 7 days. Single colonies were selected and expression of the gene of interest was induced with 1 μg/ml tetracycline (Roth, Cat# 0237.1) for 24 h before testing expression via immunoblotting.

##### CRISPR‐Cas9 gene editing

For the generation of *TMBIM5* ^−/−^ cell lines, HeLa cells were used. Cells were transfected with px335 (Addgene #42335) plasmids containing either (3′‐CCTCAGTATGTCAAGGATAGAAT‐5′) or (3′‐CCACCTATATGTACTTAGCAGGG‐5′) guide RNA for *TMBIM5*. After three transfections cells were seeded as single cells in 96‐well plates and checked for TMBIM5 protein expression after 3 weeks. Additionally, the *TMBIM5* mRNA level was tested via quantitative real‐time PCR and the genomic region of *TMBIM5* was sequenced for validation of the knockout. Gene editing results in three different mutations in exon V and the insertion of a premature STOP codon.

##### Plasmid and siRNA transfection

For transient expression of DNA, GeneJuice transfection reagent (Merck) was used. Experiments were conducted 24 h after transfection. The following siRNA targeting sequences against human proteins were purchased from (Merck): TMBIM5 (SASI_HS01_00196210), MCU (SASI_HS01_00234629), AFG3L2 (SASI_HS01_0023‐1520), NCLX (SASI_HS01_00236391). Negative control medium GC duplex was purchased from (Invitrogen; Cat# 12935300). RNA interference experiments with siRNA were conducted with Lipofectamine RNAiMAX (Thermofisher) transfection reagent. Efficient protein depletion was tested either by immunoblotting or by RT–PCR. Results related to depletion of TMBIM5 by siRNA represent the results obtained using one siRNA, which was selected among five different siRNA sequences, all of them showing similar effects. Cells were grown for either 48 or 72 h after transfection, as indicated in the Figure legends.

##### Measurement of cellular respiration

Oxygen consumption rate was measured in a Seahorse Extracellular Flux Analyzer XFe96 (Agilent) in cells grown in DMEM‐GlutaMAX containing 25 mM glucose. 4 × 10^4^ cells were plated in each well and incubated 1 h before measurement at 37°C. ATP production was assessed after the addition of oligomycin (2 μM); maximal respiration after FCCP (0.5 μM) and nonmitochondrial respiration after the addition of rotenone and antimycin A (0.5 μM).

##### Activities of respiratory chain complexes

HEK293T WT and *TMBIM5* ^−/−^ cells were cultured in DMEM (4.5 g/l glucose; Lonza) supplemented with 10% (v/v) fetal bovine serum, 1% (v/v) penicillin (10,000 units/ml)/streptomycin (10 mg/ml), 1 mM sodium pyruvate and 1% nonessential amino acids at 37°C, 5% CO_2_. Confluent cells were harvested and resuspended in ice‐cold isolation buffer (250 mM sucrose, 0.1% bovine serum albumin (BSA), 1 mM EDTA, 20 mM Tris–HCl, pH 7.4) and disrupted by 10 strokes using a Potter‐Elvehjem homogenizer with a Teflon pestle. After a clarifying spin at 1,000 *g* for 10 min at 4°C, crude mitochondria were harvested from the supernatant by sedimentation at 10,000 *g* for 10 min at 4°C. For further purification, the mitochondrial pellet was resuspended in an isolation buffer and loaded on a two‐layer (1 and 1.5 M) sucrose gradient buffered with 1 mM EDTA, 20 mM Tris–HCl, pH 7.4. After centrifugation for 20 min at 60,000 *g* (4°C), the mitochondrial layer localized at the top of the 1.5 M sucrose cushion was carefully collected and washed twice with a BSA‐free isolation buffer by centrifugation at 22,000 *g* (10 min; 4°C). Finally, mitochondria were resuspended in a small volume of BSA‐free isolation buffer. Protein concentration was determined by the Lowry method and mitochondria were shock‐frozen in liquid N_2_ and stored at −80°C until use.

For the kinetic measurements, mitochondria were diluted to 1 mg/ml in 20 mM Tris–HCl, pH 7.2 and subjected to three freeze–thaw cycles to break the inner membrane. Activities of the individual respiratory chain complexes and of citrate synthase as a reference were measured spectrophotometrically at 25°C in a SPECTRAmax PLUS^384^ microplate reader (Molecular Devices). Complex I (NADH:n‐decylubiquinone (DBQ) oxidoreductase) activity: 20 μg/ml mitochondria, 200 μM NADH, 70 μM DBQ, 3 mg/ml BSA, 2 μM antimycin A, 500 μM NaCN, 25 mM potassium phosphate, pH 7.5. NADH oxidation was followed at 340 nm (ε = 6.2 mM^−1^ cm^−1^) and corrected for the inhibitor‐insensitive rate determined in the presence of 10 μM 2‐decyl‐4‐quinazolinyl amine (DQA). Complex II (succinate:2,6‐dichlorophenol‐indophenol (DCPIP) oxidoreductase) activity: 20 μg/ml mitochondria, 10 mM Succinate, 70 μM DBQ, 80 μM DCPIP, 1 mg/ml BSA, 2 μM myxothiazol, 500 μM NaCN, 20 mM Tris–HCl pH 7.2. DCPIP reduction was followed at 600 nm (ε = 19.1 mM^−1^ cm^−1^) and corrected for the inhibitor‐insensitive rate determined in the presence of 10 mM malonate. Complex III (ubiquinol:cytochrome *c* oxidoreductase) activity: 20 μg/ml mitochondria, 75 μM ferricytochrome *c*, 100 μM *n*‐decylubiquinol (DBQH_2_), 100 μM EDTA, 500 μM NaCN, 25 mM potassium phosphate, pH 7.5. Cytochrome *c* reduction was followed at 550 nm (ε = 18.5 mM^−1^ cm^−1^) and corrected for the inhibitor‐insensitive rate determined in the presence of 2 μM myxothiazol. Complex IV (cytochrome *c* oxidase) activity: 20 μg/ml mitochondria, 50 μM ferrocytochrome *c*, 25 mM potassium phosphate, pH 7.0. Cytochrome *c* oxidation was followed at 550 nm (ε = 18.5 mM^−1^·cm^−1^) and corrected for the inhibitor‐insensitive rate determined in the presence of 500 μM NaCN. Citrate synthase activity: 10–20 μg/ml mitochondria, 1 mM oxaloacetate, 0.3 mM acetyl‐CoA, 0.1 mM 5′‐dithiobis 2‐nitrobenzoic acid (DTNB), 0.1% Triton X‐100, 100 mM Tris–HCl, pH 8.0. The reaction of DTNB with CoA formed upon citrate formation was followed at 412 nm (ε = 13.6 mM^−1^ cm^−1^).

##### Immunofluorescence

HeLa cells transfected with indicated constructs were plated on 13 mm glass coverslips in 24 well dishes. Cells were washed with room temperature PBS and fixed with 3.7% PFA in PBS at room temperature for 20 min. Cells were permeabilized in PBS containing 0.2% Triton X‐100. Samples were blocked in PBS containing 0.1% BSA for 30 min before incubating overnight with primary IF‐antibody reconstituted in blocking solution with dilutions indicated above. The secondary IF‐antibody (Invitrogen, Alexa Flour) was reconstituted in blocking buffer and incubated for 1 h, before washing the samples and mounting the coverslips onto microscopy slides with Prolong Gold Antifade Mountant (Invitrogen, Cat# P10144). Image acquisition was performed with a Leica SP8‐DLS laser‐scanning confocal microscope using a 100× oil objective (Leica HC PL APO 100×/1.4 OIL CS2; Cat# 11506372).

##### pH and Ca^2+^ measurements

Cells were plated on glass coverslips and transfected either with SypHer3s‐dmito (Addgene #108119) or with mtRED‐GECO (Addgene #46021). Imaging with a Leica SP8‐DLS laser‐scanning confocal microscope using a 40× oil objective (Leica HC PL APO 40×/1.3 OIL) was performed 24 h after transfection with the following protocol. 24 h after transfection coverslips were mounted into a custom‐made open‐topped chamber and medium exchanged with freshly prepared KRB buffer (135 mM NaCl, 5 mM KCl, 1 mM MgCl_2_, 20 mM HEPES, 1 mM MgSO_4_, 0.4 mM KH_2_PO_4_, 1 mM CaCl_2_, 5.5 mM glucose; pH 7.4 using NaOH) if not otherwise indicated at 37°C. Sypher was excited at a wavelength of 405 and 488 nm, mtRED‐GECO at a wavelength of 550 nm. Calibration of SypHer3s‐dmito was performed in calibration buffer (130 mM KCl, 10 mM NaCl, 2 mM K_2_HPO_4_, 1 mM MgCl_2_) supplemented with 20 mM MES buffer (adjusted to pH 5.5 and 6.5 with KOH) or 20 mM HEPES buffer (adjusted to pH 7.0 and 7.5 with KOH) or Tris buffer (adjusted to pH 8.0 and 9.0 with HCl) or acid boric (adjusted to pH 9.5 and 10 with KOH), pH was stepped between 5.5 and 10 by turnover of the bath solution. For each experiment monensin (5 μM) and nigericin (1 μM) were also added. The ratio of absorbance at 488 and 405 nm was calculated. Images were processed in FiJi. For each cell, a 8‐point calibration curve was fitted to a variable slope sigmoid equation.

Ca^2+^ experiments were also performed in KRB buffer. Medium was changed with KRB 10 min before the experiment. Images were acquired every 1.5 s. Thapsigargin (2 μM) was added after 200 s. Data are shown as fluorescence (F) F/F_0_ quantification, where F_0_ is the average of the first 10 s and F is the fluorescence peak measured after thapsigargin administration.

##### Mitochondrial membrane potential

A 5 × 10^5^ cells were incubated with TMRM (Thermofisher; 200 nM) containing CCCP (15 μM) or DMSO (0.01%) for 30 min at 37°C. Cells were washed with PBS, transferred to a black 96‐well plate and the fluorescence was analyzed with a plate reader (Perkin Elmer EnVision 2105).

##### Quantitative Real‐Time PCR

Isolation of mRNA from HeLa cells was performed with NucleoSpin RNA kit (Macherey‐Nagel; 740,955.250) before generating cDNA with Promega GoScript Reverse Transcriptase kit (Cat# A5003) and adjusting to 10 μg/ml. PCR was performed with SYBR Green PCR mix (applied biosystems; Cat# 4309155), 0.6 μM of each primer, and 30 ng cDNA for 40 rounds at 95°C melting and 60°C elongation steps. Actin and GADPH were used as housekeeping genes for normalization. Primer sequences are listed in the Reagent Tools table.

##### SDS–PAGE and immunoblotting

Cells were collected and washed with PBS, before lysing with lysis‐buffer (1 M (w/v) Tris–HCl pH 8.5, 0.5 M (w/v) EDTA, 10% (w/v) SDS, 3 M (w/v) NaCl) containing complete protease inhibitor cocktail (Roche; Cat# 4693132001). Protein concentration was determined via Bradford assay (BioRad). Protein solution was adjusted to equal concentration and incubated for 20 min at 40°C in an SDS‐sample buffer (2% SDS, 10% glycerol, 75 mM Tris–HCl pH 6.8, 0.02% bromphenol blue, 5% beta‐mercaptoethanol). SDS‐PAGE gels were prepared with acrylamide/bisacrylamide (ratio 97%/3%) concentrations of 4% for the stacking and 10% for the separating gel. Protein size was determined via Sea‐Blue Plus 2 prestained protein standards (Invitrogen; Cat# LC5925). Immunoblotting of PAGEs was conducted onto Amersham nitrocellulose or PVDF membranes (GE Healthcare) for 2 h at 1.6 mA/cm^2^ using blotting buffer (25 mM Tris, 185 mM glycine, 20% methanol). Membranes were blocked in 5% milk powder in TBS containing 0.05% Tween‐20.

##### Cell treatments

Protein stability was followed by treatment with cycloheximide (Sigma; Cat# C4859) or emetine (Sigma; Cat# E2375) at a concentration of 100 μg × ml^−1^ over the course of 8 h. At indicated time points, cells were collected and lysed according to the protocol above. Cell viability was assessed after treatment with actinomycin D (Sigma, Cat# A9415; 1 μM), venetoclax (Selleckchem; Cat# S8048; 1 μM), A‐1155463 (Selleckchem; Cat# S7800; 1 μM), staurosporine (Sigma, Cat# S6942; 1 μM) or thapsigargin (Sigma; Cat# T9033; 2 μM) for 16 h. ATP synthase was inhibited by oligomycin treatment (Sigma, Cat# O4876; 10 μM) for 18 h.

##### Mitochondrial isolation

For mitochondrial isolation, cell pellets were resuspended in isolation buffer (220 mM mannitol, 70 mM sucrose, 2 mM EGTA, 20 mM HEPES‐KOH pH 7.4, 1 mM PMSF (Roche, Cat# 10837091001), complete protease inhibitor cocktail (Roche, Cat# 52434800), 0.1% (BSA)) and incubated for 15 min. Cells were disrupted using 15 strokes in a glass mortar and PTFE pestle at 1,000 rpm with a rotating homogenizer (Schuett Biotec, Cat# 3201011). The mitochondrial‐containing fraction was separated at 1,000 *g* for 15 min and subsequently pelleted at 8,000 *g* for 15 min before washing in isolation buffer without BSA. Freshly isolated mitochondria were used for experiments relying on intact mitochondrial membrane potential.

##### Immunoprecipitation

Isolated mitochondria were incubated for 15 min in lysis buffer (20 mM HEPES‐NaOH pH7.5, 300 mM NaCl) containing 4 g digitonin per g mitochondria. Centrifuged lysate was incubated with washed EZview Red anti‐FLAG M2 affinity gel (Millipore, Cat# F2426) for 1 h. Beads were washed (10 mM HEPES‐NaOH pH7.5, 150 mM NaCl, 0.1% Triton X‐100) three times before analyzing via SDS–PAGE or mass spectrometry‐based proteomics.

##### Fluorescence‐activated cell sorting (FACS)

###### NucView 488 RedDot 2

HeLa cells were plated 20 × 10^4^ each 6 well. 24 h later they were treated with staurosporine (0.1 μM) for 16 h. Cells were detached using trypsin and resuspended at a density of 10^6^ cells/ml in a culture medium that contain NucView 488 substrate (2.5 μM) and RedDot 2 (0.25×). Cells were incubated at room temperature for 30 min protected from light, resuspended in PBS, and analyzed by flow cytometry. NucView fluorescence was measured in the green detection channel (ex488/em530 nm). RedDot 2 fluorescence in the far‐red channel (ex640/em780 nm).

###### MitoSOX

HeLa cells were plated 20 × 10^4^ each 6 well. 24 h later were detached using trypsin, resuspended at a density of 10^6^ cells/ml in PBS‐containing MitoSOX Red for 30 min, and then analyzed using flow cytometry. Rotenone (0.5 μM) was added during MitoSox Red staining.

##### EM

Cells were fixed in fixation buffer (2% glutaraldehyde, 2.5% sucrose, 3 mM CaCl_2_, 100 mM HEPES‐KOH pH 7.2) at room temperature for 30 min and 4°C for 30 min. Samples were washed three times in 1% osmium tetroxide, 1.25% sucrose, and 1% potassium ferrocyanide in 0.1 M sodium cacodylate buffer. After dehydration in alcohol gradient series and propylene oxide, the tissue samples were embedded. Ultrathin sections were cut on a diamond knife (Diatome, Biel, Switzerland) on a Leica ultramicrotome and placed on copper grids (Science Services, 100mesh). Sections were stained with uranyl acetate (Plano, 1.5%) and lead citrate (Sigma) and examined with an electron microscope (JEM 2100 Plus, JEOL) with a OneView 4 K camera (Gatan) with DigitalMicrograph software at 80 kV.

##### Calcium retention capacity

Isolated mitochondria from HEK293T cells were resuspended in assay buffer (100 mM sucrose, 4 mM MgCl_2_, 80 mM KCl, 5 mM Na‐succinate, 20 mM HEPES‐KOH pH 7.6, 0.4 mM ADP, 0.5 mM NADH, 0.01 mM EGTA, 0.5 μM Calcium‐Green 5N (Thermofisher), 2 mM KH_2_PO_4_) to a concentration of 1 μg × μl^−1^. Mitochondria were subjected to 10 μl calcium chloride injections until disruption. Fluorescence was measured in a Perkin Elmer EnVision 2105 plate reader at excitation of 485 nm and emission of 535 nm.

##### Protein expression and purification

Codon‐optimized DNA sequence for expression in *E. coli* of the human TMBIM5 protein was synthesized from PCR fragments by GeneArt (Life Technologies) and subsequently cloned into the bacterial overexpression vector pMALp2x (NEB). The recombinant TMBIM5 protein expression was performed in BL21(DE3) *E. coli*. The bacteria were propagated in a TB medium. Protein expression was induced with 0.5 mM IPTG and was carried out at 37°C for 4 h in an orbital shaker. Bacterial cells were harvested at 3,000 *g* for 20 min, washed in 200 mM NaCl, 5% glycerol, 1 mM EDTA, 20 mM HEPES‐KOH pH 7.4, and stored at −20°C. Bacterial pellets were resuspended in buffer (200 mM NaCl, 5% glycerol, 1 mM EDTA, 1 mM DTT, 20 mM HEPES‐KOH pH7.4), which was supplemented with protease inhibitor cocktail (Roche) and lysed in an Emulsiflex. The obtained cell lysate was supplemented with 0.05% DDM and spun down at 180,000 *g* at 4°C for 20 min. The supernatant was applied onto MBPTrap affinity column (Cytiva; Cat# 28918780) and the bound fraction was subsequently eluted with 10 mM maltose. Protein‐containing fractions were subjected to Factor Xa digest (NEB). Resulting mixture of untagged TMBIM5 and free MBP‐tag was separated on HiLoad Superdex 16/600 75 pg column equilibrated in 120 mM N‐methyl‐D‐glutamate (NMDG), 1 mM DTT, 0.05% DDM, 10 m MOPS‐KOH pH 7.4.

##### Preparation of TMBIM5‐proteoliposomes

All lipids used for the preparation of liposomes were purchased from Avanti Polar Lipids. The lipid mixture mimicking the composition of the mitochondrial inner membrane contained 45% L‐α‐phosphatidylcholine, 20% L‐α‐phosphatidylethanolamine, 15% L‐α‐phosphatidyl‐inositol, 15% cardiolipin and 5% L‐α‐phosphatidylserine. Lipid stocks prepared in methanol/ chloroform mixture were dried under constant nitrogen flow. Dried lipid films were re‐hydrated in 120 mM NMDG, 10 mM MOPS‐KOH pH 7.4 buffer supplemented with 100 μM of Fura‐2 (Invitrogen, Cat# F1200) for 10 min and subjected to the sequence of freeze‐and‐thaw cycles of 10 repetitions. The obtained lipid vesicles were extruded through a PVDF filter with a pore diameter of 400 nm and used for protein incorporation. Prior to protein incorporation, Fura‐2‐containing liposomes were partially solubilized with 0.1% DDM and incubated for 30 min at room temperature under gentle shaking. Purified TMBIM5 protein was added to the presolubilized liposomes in a 100 to 1 (w/w) ratio and incubated for 1 h to allow for spontaneous protein insertion. The incorporation process was promoted by removing the detergent from the mixture using adsorbent BioBeads SM‐2 (BioRad). Formed proteoliposomes were separated from the BioBeads and the proteoliposomes were harvested by centrifugation at 180,000 *g* for 20 min at 4°C, washed with 120 mM NMDG, 10 mM MOPS‐KOH pH8 and used for Ca^2+^ flux measurements.

Ca^2+^ flux was initiated by adding CaCl_2_ (100 μM). The fluorescence intensity was monitored every 1 s in a Perkin Elmer EnVision 2105 plate reader at excitation 340 and 380 nm, emission 510 nm. Ionomycin (10 mM) was added at the end of the experiment for calibration. GdCl3 (100 μM) was added immediately before the experiment was indicated.

##### Electrophysiological characterization of TMBIM5

Electrophysiological characterization of TMBIM5 was carried out using the planar lipid bilayer technique (Denkert *et al*, 2017; Vasic *et al*, 2020). Electrical current recordings were performed using Ag/AgCl electrodes in glass tubes, embedded in a 2 M KCl agar bridge. The electrode in the trans chamber was connected to the headstage (CV‐5‐1GU) of a Geneclamp 500B current amplifier (Molecular Devices, CA, USA) and acted as a reference electrode. Data were acquired using a Digidata 1440A A/D converter and recorded using the AxoScope 10.3 and Clampex 10.3 software (Molecular Devices). Data analysis was carried out using OriginPro 8.5G (OriginLab, MA, USA).

TMBIM5‐proteoliposomes were inserted into the planar lipid bilayer by osmotic driven fusion in asymmetric buffer condition with 250 mM KCl, 10 mM MOPS‐KOH, pH 7.0 in the cis chamber and 20 mM KCl, 10 mM MOPS‐KOH, pH 7.0 in the trans chamber. After incorporation of TMBIM5 into the planar lipid bilayer, both of the chambers were perfused with 250 mM CaCl_2_, 10 mM MOPS‐KOH, pH 7.0 to attain symmetrical buffer conditions and voltage‐clamp recordings, and voltage ramps were performed. To investigate the inhibitory effects of Gd^3+^ ions on TMBIM5, 9 mM GdCl_3_ was added to both cis and trans chambers and stirred for 2 min before the current recordings. Voltage ramps were recorded from −80 mV to +80 mV and the current–voltage relationship was plotted. Current traces were recorded at constant voltage for 1 min. The open probability (*P*_open_) was calculated from the maximum current recorded, divided by the mean current using the data from three experimental replicates.

#### Proteomics

##### Determination of the AFG3L2 interactome by data‐dependent acquisition

###### Protein digestion

Proteins bound to the EZview Red anti‐FLAG M2 affinity gel (Millipore; catalog number F2426) beads were lysed using 60 μl of 4% SDS in 100 mM HEPES‐KOH pH 8.5 at 70°C for 10 min on a ThermoMixer with shaking (550 rpm). Proteins were reduced (10 mM TCEP) and alkylated (20 mM CAA) in the dark for 45 min at 45°C and processed via the SP3 digestion protocol (Hughes *et al*, 2019). Washed SP3 beads (Sera‐Mag(TM) Magnetic Carboxylate Modified Particles (Hydrophobic, catalog number GE44152105050250), Sera‐Mag(TM) Magnetic Carboxylate Modified Particles (Hydrophilic, catalog number GE24152105050250) from Sigma Aldrich) were mixed equally, and 3 μl of bead slurry were added to each sample. Acetonitrile was added to a final concentration of 50% and washed twice using 70% ethanol (V = 200 μl) on an in‐house‐made magnet. After an additional acetonitrile wash (V = 200 μl), 5 μl 10 mM HEPES pH 8.5 containing 0.5 μg trypsin (Sigma, #T6567‐1MG) and 0.5 μg LysC (Wako, # 129‐02541) were added to each sample and incubated overnight at 37°C. Peptides were desalted on a magnet using 2 × 200 μl acetonitrile. Peptides were eluted in 10 μl 5% DMSO in LC–MS water (Sigma Aldrich) in an ultrasonic bath for 10 min. Samples were dried and stored at −20°C until subjected to LC–MS/MS analysis. Peptides were then reconstituted in 10 μl of 2.5% formic acid and 2% acetonitrile and 3 μl were used for an LC–MS/MS run.

###### Liquid chromatography and mass spectrometry

LC–MS/MS instrumentation consisted of an Easy‐LC 1200 (Thermo Fisher Scientific) coupled via a nano‐electrospray ionization source to a QExactive HF‐x mass spectrometer (Thermo Fisher Scientific). For peptide separation, an in‐house packed column (inner diameter: 75 μm, length: 40 cm) was used. A binary buffer system (A: 0.1% formic acid and B: 0.1% formic acid in 80% acetonitrile) was applied as follows: Linear increase in buffer B from 4% to 32% within 33 min, followed by a linear increase to 55% within 5 min. The buffer B content was further ramped to 95% within 2 min. 95% buffer B was kept for further 5 min to wash the column. Prior to each sample, the column was washed using 6 μl buffer A and the sample was loaded using 7 μl buffer A.

The mass spectrometer operated in a data‐dependent mode and acquired MS1 spectra at a resolution of 60,000 (at 200 *m*/*z*) using a maximum injection time of 20 ms and an AGC target of 3e6. The scan range was defined from 350–1,650 *m*/*z*, and the data type was set to profile. MS2 spectra were acquired at a 15,000 resolution (at 200 *m*/*z*) using an isolation window of 1.4 *m*/*z* and a normalized collision energy of 32. The Top22 peaks were targeted for MS2 spectra acquisition. The first mass was set to 110 *m*/*z*. Dynamic exclusion was enabled and set to 20 s.

###### Data analysis of data‐dependent acquisition proteomics

The Andromeda Search Engine implemented into the MaxQuant software (1.6.0.1) was used to analyze DDA files to investigate the AFG3L2 interactome. MS/MS spectra were correlated against the UniProt reference proteome (downloaded Nov. 2019, containing 20,362 protein amino acid sequences) using default settings. The match‐between runs algorithm was enabled and the label‐free quantification feature (min. Count = 1). The protein groups output file was then further processed in Perseus by log2 transforming the LFQ intensities. Protein groups were then filtered for being quantified in 5 out of 5 replicates followed by imputation of missing values from a down‐shifted (downshift: 1.8* standard dev., width: 0.4* standard dev.) gaussian distribution. To identify potential interactors, we used a two‐sided *t*‐test followed by permutation‐based FDR estimation to correct for multiple testing (FDR < 0.05). Gene Ontology annotations were added, and data were filtered solely for visualization based on the localization of the mitochondria using the GO cellular component information.

##### Whole cell proteomics using data‐independent acquisition

###### Protein digestion

A 40 μl of 4% SDS in 100 mM HEPES‐KOH pH 8.5 was preheated to 70°C and added to the cell pellet for further 10‐min incubation at 70°C on a ThermoMixer (shaking: 550 rpm). The protein concentration was determined using the 660 nm Protein Assay (ThermoFisher Scientific, catalog number 22660). 50 μg of protein was subjected to tryptic digestion. Proteins were reduced (10 mM TCEP) and alkylated (20 mM CAA) in the dark for 45 min at 45°C. Samples were subjected to SP3‐based digestion as described above (Hughes *et al*, 2019). Generated peptides were eluted from magnetic beads in 10 μl 5% DMSO in LC–MS water (Sigma Aldrich) in an ultrasonic bath for 10 min. Samples were then dried and stored at −20°C until subjected to LC–MS/MS analysis. Peptides were then reconstituted in 10 μl of 2.5% formic acid and 2% acetonitrile and 3 μl were used for an LC–MS/MS run.

###### Liquid chromatography and mass spectrometry

LC–MS/MS instrumentation consisted of an Easy‐LC 1200 (Thermo Fisher Scientific) coupled via a nano‐electrospray ionization source to an Exploris 480 or QExactive HF‐x mass spectrometer (Thermo Fisher Scientific). For peptide separation, an in‐house packed column (inner diameter: 75 μm, length: 40 cm) was used. A binary buffer system (A: 0.1% formic acid (Thermo Fisher Scientific, cat# LS118‐1) and B: 0.1% formic acid in 80% acetonitrile (Thermo Fisher Scientific, cat# LS122)) was applied as follows: Linear increase of buffer B from 4% to 27% within 69 min, followed by a linear increase to 45% within 5 min. The buffer B content was further ramped to 65% within 5 min and then to 95% within 6 min. 95% buffer B was kept for further 10 min to wash the column. Prior to each sample, the column was washed using 5 μl buffer A and the sample was loaded using 8 μl buffer A.

The RF Lens amplitude was set to 55%, the capillary temperature was 275°C and the polarity was set to positive. MS1 profile spectra were acquired using a resolution of 60,000 (at 200 *m*/*z*) at a mass range of 320–1,150 *m*/*z* and an AGC target of 1 × 106.

For data‐independent MS/MS spectra acquisition, 48 windows were acquired at an isolation *m*/*z* range of 15 Th, and the isolation windows overlapped by 1 Th. The fixed first mass was 200. The isolation center range covered a mass range of 350–1,065 *m*/*z*. Fragmentation spectra were acquired at a resolution of 15,000 at 200 *m*/*z* using a maximal injection time of 22 ms and stepped normalized collision energies (NCE) of 26, 28, and 30. The default charge state was set to 3. The AGC target was set to 900% (Exploris 480) or 1e6 (QExactive HF‐x). MS2 spectra were acquired as centroid spectra.

###### Data analysis

For the analysis of DIA (Data independent acquisition) experiments, except pulseSILAC (see below), we utilized DIA‐NN version 1.7.15 (Demichev *et al*, 2020). The library‐free approach was used based on the human Uniport reference proteome (downloaded Nov. 2019, containing 20,362 protein amino acid sequences), which predicts MS2 spectra using neuronal network. The deep‐learning option was enabled. Quantification strategy was set to “robust LC (high accuracy).” The precursor range was adjusted to 330–1,200 *m*/*z* matching the acquired mass range. The RT profiling option was enabled. Otherwise, default settings were used. To identify significantly different proteins, a two‐sided *t*‐test was applied using log2 transformed MaxLFQ intensities calculated by DIA‐NN (Cox *et al*, 2014). The FDR was controlled to 5% using a permutation‐based approach in the Perseus software (Tyanova *et al*, 2016). If applicable, a two/three ANOVA was calculated in the Perseus software. Raw output of DIA‐NN analysis and raw files were deposited to the PRIDE repository. Blinding of samples was not performed.

*Pulse SILAC data*: To analyze pulseSILAC DIA data, a library was first created using acquired DIA files Spectronaut's Pulsar search engine using exclusively y‐ions but otherwise default settings. Then, in a second analysis, the individual files were analyzed using the generated spectra library. Data were exported on the precursor level and were transformed as follows. First, the remaining heavy fraction was determined by calculating the natural logarithm of the labeled fraction H/(H + L) = H/L/(H/L + 1) representing the intensity of light (L) and heavy (H) labeled peptides. Second, the data were adjusted by calculating the median of the labeled fraction for each group (e.g., timepoint and genotype), and individual replicates (*n* = 6 biological replicates) were adjusted to the overall group median. Then, data were filtered by removing values that were found to be outliers (1.8× inter quantile range), which were replaced by the group mean. To identify the turnover rate, a linear model was fit to the data assuming a first‐order kinetic and the *r*‐value, *P*‐value, slope, and intercepts, and their standard errors were calculated for each individual replicates. Slope values with an *r*‐value above −0.75 were removed by replacement with NaN. The slope represents the turnover rate [1/h] constant and was used to identify differently turned‐over proteins using a two‐sided *t*‐test performed, and significance was considered for proteins with *P* < 0.01. Analysis was performed using the Instant Clue (v 0.11.1) software suite (www.instantclue.de; Nolte *et al*, 2018).

##### Blue‐native PAGE complexome profiling of mitochondria isolated from WT and *TMBIM5* ^−/−^ HEK293 cells

###### Sample preparation

Separation of native protein complexes from membrane‐enriched mitochondrial fractions was performed using Blue‐native PAGE (BN–PAGE) as previously described (Heide *et al*, 2012).

###### Protein digestion

Each lane of the BN–PAGE was cut into 72 slices of equal size. Each slice was digested in gel using Trypsin and subsequently analyzed by LC–MS/MS. Briefly, the gel pieces were destained in 50 mM ammonium bicarbonate and ethanol. Then, proteins were reduced using 10 mM dithiothreitol (DTT) at 56°C for 45 min, carbamidomethylated with 55 mM iodoacetamide in the dark for 30 min at room temperature. Proteins were digested using 12 ng/μl LysC (Wago) /Trypsin (Sigma Aldrich) (1:3 ratio) at 37°C overnight. Then, the digestion was stopped by acidification with trifluoracetic acid (TFA) to a final concentration of 0.5%, and peptides were extracted from the gel pieces with increasing concentrations of acetonitrile (ACN). The organic solvent was vacuum evaporated using a SpeedVac concentrator plus (Eppendorf, Hamburg, Germany), and peptides were desalted using self‐made C18‐based Stop and Go Extraction Tips.

###### Liquid chromatography and mass spectrometry

Data were acquired using a data‐dependent acquisition strategy on an nLC1000 (Thermo Fisher Scientific) coupled to a QExactive HF mass spectrometer. The total gradient length was 25 min separating peptides using a binary buffer system of (A) 0.1% formic acid in H_2_O and (B) 0.1% formic acid in 80% acetonitrile. The spray voltage was 2,000 V and the capillary temperature was set to 250°C. MS1 Spectra were acquired using a resolution of 120,000, an AGC target of 3e6, and a maximum injection time of 100 ms. For MS2 spectra, a maximum injection time of 120 ms and an AGC target of 1e6. The isolation width was set to 1.6 Th. The normalized HCD energy was 25.

##### Data analysis

The acquired data‐dependent acquisition mass spectra were correlated to the human Uniprot database containing only reviewed sequences (download 12.2021, 20,326 protein sequences) using the MaxQuant software (2.0.3.0) and the implemented Andromeda search engine. The match‐between runs algorithm has been enabled. The iBAQ intensity was calculated and used for further analysis. The MitoCarta3.0 pathway annotations were used to identify Complex I and III subunits. Assembly factors are not included. Profiles for Complex I subunits and Complex III subunits showing the median as a line and the inter quantile range (IQR) as a transparent area were plotted using the Instant Clue software.

#### Quantification and statistical analysis

Densitometry data for western blots were generated using Fiji. Representative images of at least three independent experiments are shown. Graphs were plotted using Prism (GraphPad). Volcano plots and heatmaps were generated with InstantClue. Error bars represent the standard deviation of the mean. Statistical significance of the data was mostly assessed by using the Student's *t*‐test, comparing control and test conditions, and it is described in the figure legends.

## Author Contributions

**Thomas Langer:** Conceptualization; supervision; writing – original draft; writing – review and editing. **Maria Patron:** Conceptualization; formal analysis; validation; investigation; visualization; methodology; writing – original draft. **Daryna Tarasenko:** Data curation; formal analysis; investigation; methodology. **Hendrik Nolte:** Data curation; formal analysis; validation; investigation; visualization; methodology; writing – review and editing. **Mausumi Ghosh:** Data curation; formal analysis; validation; investigation; visualization; methodology. **Yohsuke Ohba:** Investigation; methodology. **Yvonne Lasarzewski:** Investigation; methodology. **Zeinab Alsadat Ahmadi:** Investigation; methodology. **Alfredo Cabrera‐Orefice:** Data curation; formal analysis; investigation; methodology. **Akinori Eyiama:** Data curation; formal analysis; investigation; methodology. **Tim Kellermann:** Data curation; formal analysis; investigation; methodology. **Elena I Rugarli:** Conceptualization; investigation; methodology. **Ulrich Brandt:** Data curation; formal analysis; investigation; methodology. **Michael Meinecke:** Data curation; formal analysis; validation; investigation; visualization; methodology. **Lara Kroczek:** Validation; investigation; visualization; methodology.

In addition to the CRediT author contributions listed above, the contributions in detail are:

M.P. and T.L. wrote the manuscript; M.P. designed and performed most of the experiments and analyzed the data with contributions from L.K, Y.L., T.K., and A.E.; D.T. prepared liposomes containing TMBIM5; M.G. and M.M. performed electrophysiology and analyzed the data; H.N. and M.P. designed, performed, and analyzed the proteomic experiments; Y.O. generated TMBIM5 HeLa KO cells; Y.L. performed AFG3L2 interactome; Z.A.A., A.C‐O, and U.B. performed the analysis of respiratory chain complex activities. All authors commented on and edited the manuscript.

## Disclosure and Competing Interest Statement

The authors declare that they have no conflict of interest.

## Supporting information




Appendix S1

Expanded View Figures PDF

Dataset EV1

Dataset EV2

Dataset EV3

Dataset EV4

Dataset EV5

Dataset EV6

Dataset EV7

Source Data for Expanded View

Source Data for Figure 1

Source Data for Figure 2

Source Data for Figure 5

Source Data for Figure 6

## Data Availability

The proteomics data were submitted to the PRIDE repository. For proteomic data expect the BN–PAGE base complex profiling: PXD030095 (https://www.ebi.ac.uk/pride/archive/projects/PXD030095). The BN–PAGE complexome profiling data are available under the accession: PXD033084 (https://www.ebi.ac.uk/pride/archive/projects/PXD033084).
