# Supplementary material for: Regulation of mitochondrial proteostasis by the proton gradient
Source: EMBO J. 2022 Aug 1;41(16):e110476. doi: 10.15252/embj.2021110476 (PMC9379554; doi:10.15252/embj.2021110476)
Supplement: Supplementary file 1 — Appendix S1 [file EMBJ-41-e110476-s003.pdf]

Appendix

Table of content

(A) Transcript levels of complex I subunits monitored by qPCR.

(B) Workflow of SILAC chase experiments in WT and *TMBIM5*$^{-/-}$ HeLa cells.

(C) Cell growth of WT and *TMBIM5*$^{-/-}$ HeLa cells determined with an Incucyte live-cell analysis system.

(D) Scatter plot of significantly (p-value < 0.01, two-sided t-test) different turnover rate constants between WT and *TMBIM5*$^{-/-}$ cells. MitoCarta 3.0 positive proteins as well as complex I subunits are highlighted by color. The blue line indicates an identity function f(x) = y.

(E) Boxplot showing the distribution and individual data points of the natural logarithm of the labeled fraction (H/L/(H/L+1)) of WT and *TMBIM5*$^{-/-}$ for NDUFA9 and NDUFB9.

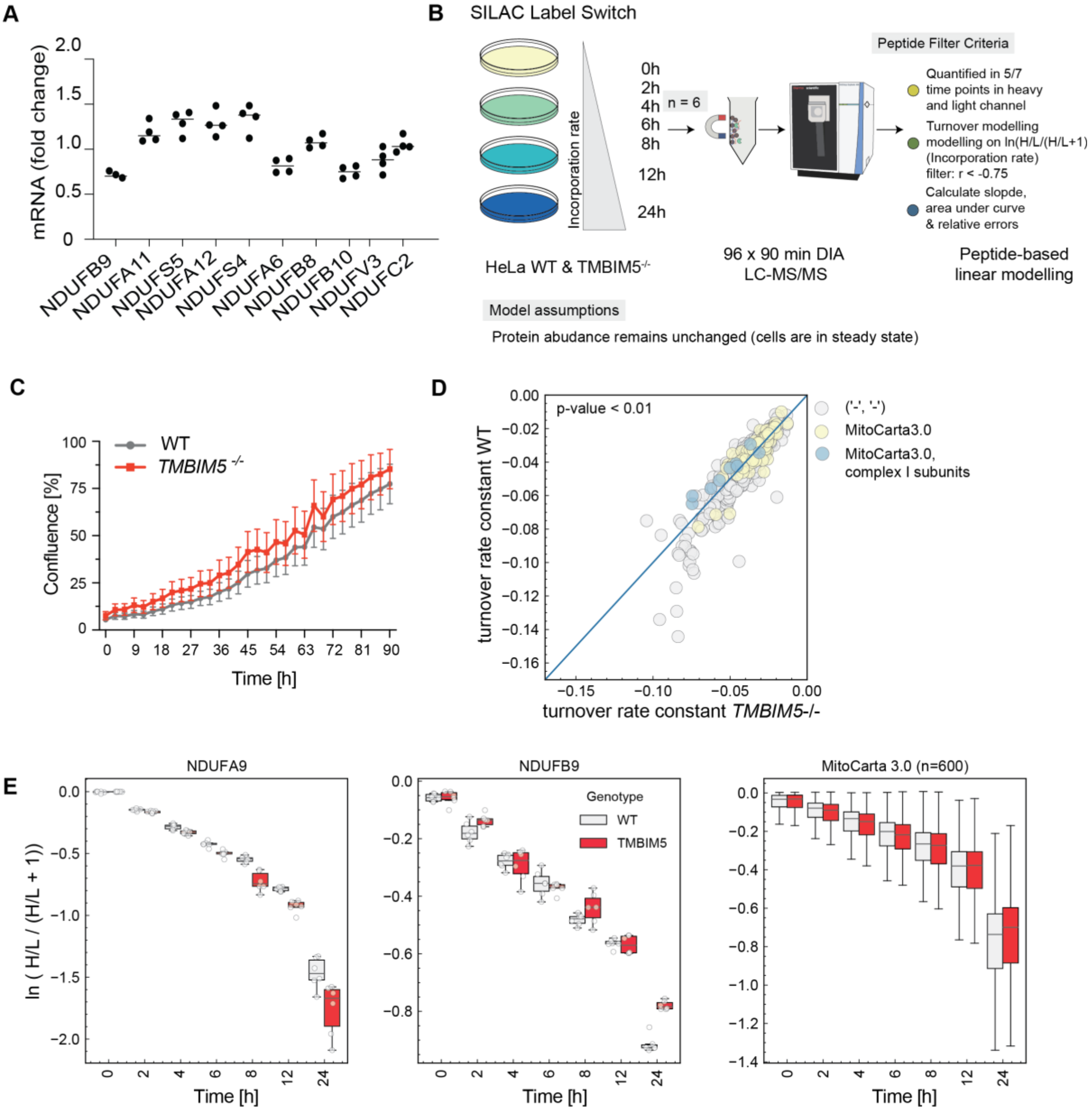

Appendix Figure S1
