# Supplementary material for: Regulation of mitochondrial proteostasis by the proton gradient
Source: EMBO J. 2022 Aug 1;41(16):e110476. doi: 10.15252/embj.2021110476 (PMC9379554; doi:10.15252/embj.2021110476)
Supplement: Supplementary file 2 — Expanded View Figures PDF [file EMBJ-41-e110476-s008.pdf]

Expanded View Figures

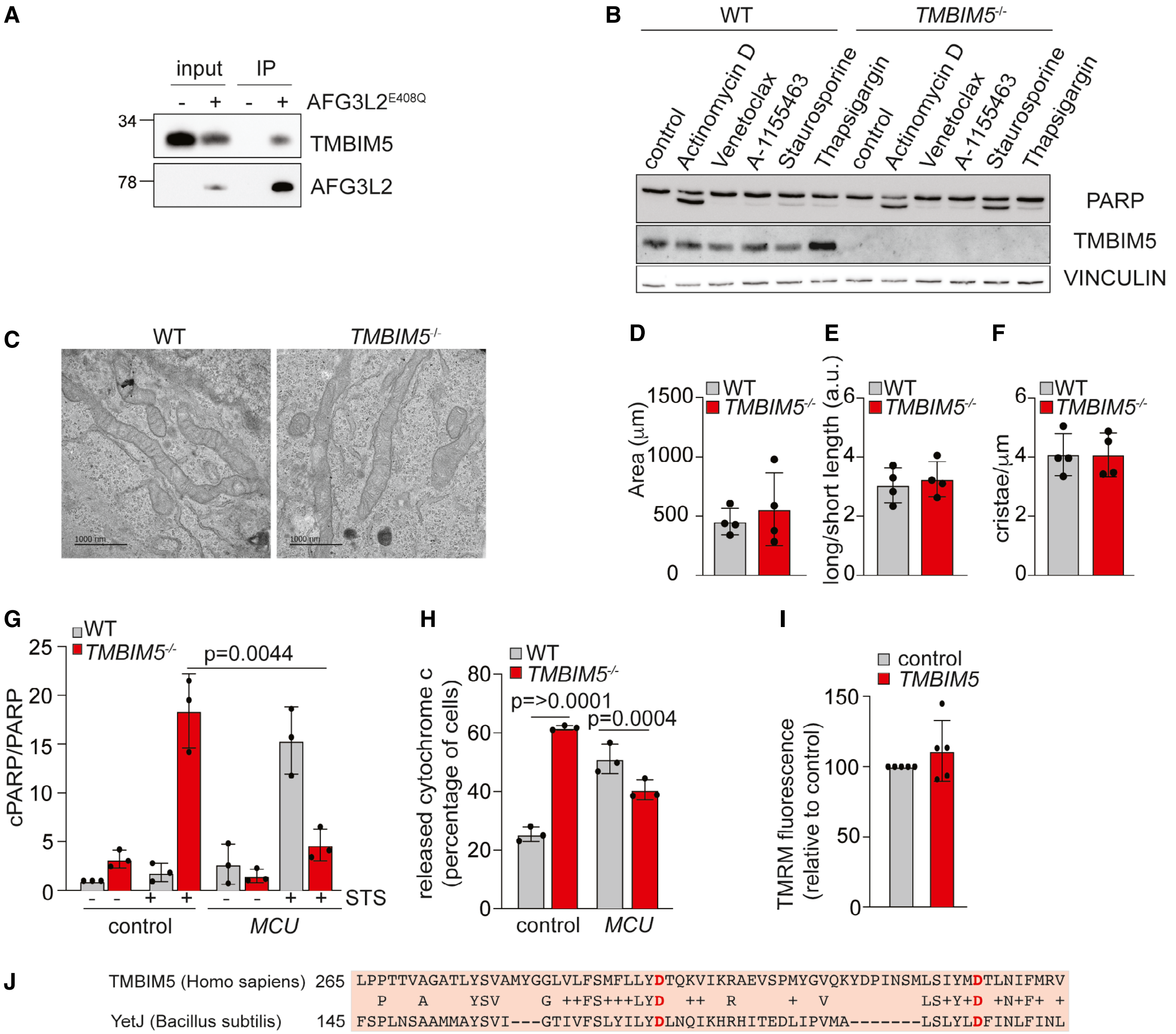

© 2022 The Authors

Figure EV1. Mitochondrial Ca^{2+} overload triggers apoptotic death of *TMBIM5*$^{-/-}$ cells (Related to Fig 1).

A Representative immunoblot of immunoprecipitates using anti-FLAG M2 beads of mitochondrial lysates, which were isolated from Flp-In HEK293T-REx cells and from Flp-In HEK293T-REx cells expressing AFG3L2$^{E408Q\ FLAG}$.

B Representative immunoblot of wildtype (WT) and *TMBIM5*$^{-/-}$ HeLa cells treated with the indicated drugs for 16 h. Control (0.1% DMSO), actinomycin D (1 μM), venetoclax (1 μM), A-1155463 (1 μM), staurosporine (1 μM), thapsigargin (2 μM). $n = 3$ independent experiments.

C Representative transmission electron microscopy images of WT and *TMBIM5*$^{-/-}$ HeLa cells. Mitochondrial morphology is shown. Scale bar 1 μm. ($n = 4$ independent biological replicates, 300 mitochondria of at least six cells, mean ± SD).

D Quantification of the electron microscopy images of WT and *TMBIM5*$^{-/-}$ HeLa cells shown in Fig EV1C. Mitochondrial parameters (area, length, and cristae) were calculated with FiJi. Values are mean ± SD.

E Quantification of the electron microscopy images of WT and *TMBIM5*$^{-/-}$ HeLa cells shown in Fig EV1C. The ratio between the long length and the short length of mitochondria is shown. Values are mean ± SD.

F Quantification of electron microscopy images of WT and *TMBIM5*$^{-/-}$ HeLa cells shown in Fig EV1C. Number of cristae per mitochondrial length (μm) is shown. Values are mean ± SD.

G Quantification of Fig 1C. The ratio between cleaved PARP (cPARP; 89 kDa) and PARP (116 kDa). WT and *TMBIM5*$^{-/-}$ HeLa cells transfected with scrambled siRNA (control) or siRNA targeting *MCU* for 72 h. When indicated, samples were treated with staurosporine (STS; 1 μM) for 16 h. $n = 3$ independent experiments. Data are shown as mean ± SD.

H Quantification of WT and *TMBIM5*$^{-/-}$ HeLa cells with cytoplasmic cytochrome *c*. After transfection with scrambled siRNA (control) or siRNA targeting *MCU* for 72 h cells were incubated for 16 h with staurosporine (0.1 μM) in the presence of Z-VAD-FMK (50 μM) and epoxomicin (1 μM) to prevent apoptosis. Cells were analyzed by immunofluorescence microscopy using antibodies directed against cytochrome *c* (green) and TOMM20 (magenta). $n = 3$ independent experiments; number of cells > 150 in each experiment. Mean ± SD.

I Mitochondrial membrane potential was monitored by TMRM staining in WT HeLa cells transfected with scrambled siRNA (control) or siRNA targeting *TMBIM5* for 72 h. Fluorescent intensity was calculated relative to the value upon CCCP addition (15 μM). Values are expressed as mean relative to control. Measurements of five different preparations were performed in triplicate. Mean ± SD.

J Sequence alignment of TMBIM5 (Q9H3K2, *Homo sapiens*) and YetJ (O31539, *Bacillus subtilis*). Conserved amino acids between the two species are written in the middle line. Amino acids forming the di-aspartyl dyad are indicated.

Source data are available online for this figure.

 © 2022 The Authors

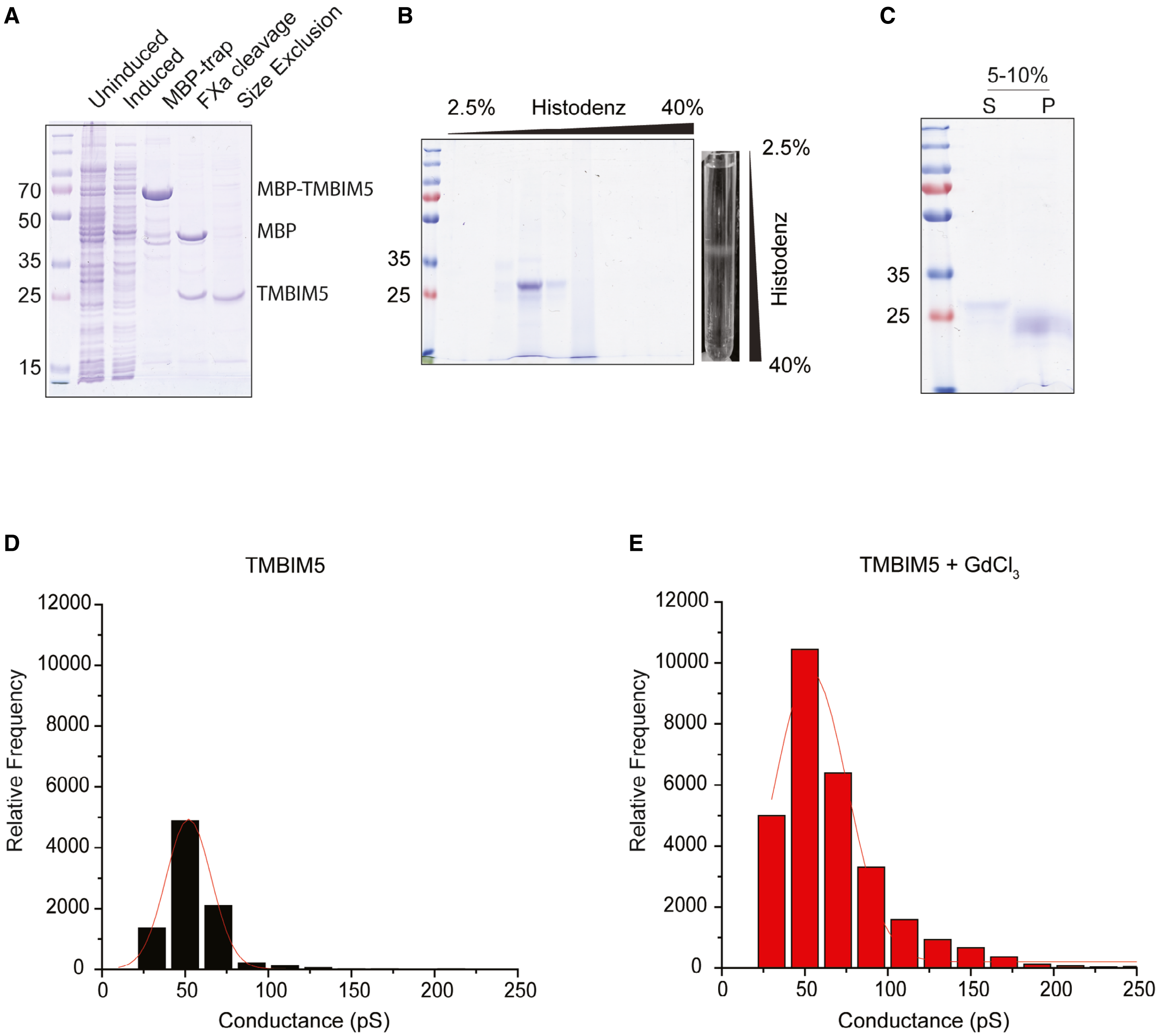

Figure EV2. Reconstitution of TMBIM5 into liposomes (Related to Figs 2 and 3).

A Representative Coomassie-stained SDS-polyacrylamide gel (12.5%) demonstrating enrichment of recombinant TMBIM5 protein upon purification.

B Flotation assay demonstrating co-migration of recombinant TMBIM5 protein with the liposomes after ultracentrifugation in a discontinuous histodenz gradient. A representative Coomassie-stained SDS-polyacrylamide gel (12.5%) is shown. The ultracentrifugation tube containing TMBIM5-liposomes in discontinuous histodenz gradient after ultracentrifugation but before fractionation is shown on the right.

C Sodium bicarbonate extraction. Supernatant (S) and pellet (P) fractions of TMBIM5-proteoliposomes after treatment with 0.1 M sodium bicarbonate (pH 11.5) analyzed by SDS-polyacrylamide gel (12.5%) electrophoresis. Pellet (P), and supernatant (S) are indicated in the figure. The presence of TMBIM5 in the pellet fraction indicates its integration into the lipid bilayer.

D TMBIM5 conductance state histogram in $CaCl_2$ buffer. n = 3 independent experiments.

E TMBIM5 conductance state histogram in $CaCl_2$ buffer containing $GdCl_3$. n = 3 independent experiments.

© 2022 The Authors

Figure EV3. TMBIM5 controls oxidative phosphorylation (Related to Fig 4).

A Oxygen consumption rate (OCR) of wildtype (WT) and *TMBIM5*$^{-/-}$ HEK293T cells in glucose media. Traces are mean ± SD of six independent experiments, each one run at least in triplicate. Labeled lines denotes injections of oligomycin (Oligo, 2 μM), FCCP (0.5 μM), rotenone and antimycin A (Rot + AA; both 0.5 μM).

B Basal respiration calculated from OCR experiment of WT and *TMBIM5*$^{-/-}$ HEK293T cells in (A). Mean ± SD; two-tailed *t*-test. A *P*-value of < 0.05 was considered statistically significant.

C Maximal respiration calculated from OCR experiment of WT and *TMBIM5*$^{-/-}$ HEK293T cells in (A). Mean ± SD; two-tailed *t*-test. A *P*-value of < 0.05 was considered statistically significant.

D ATP production calculated from OCR experiments of WT and *TMBIM5*$^{-/-}$ HEK293T cells in (A). Mean ± SD; two-tailed *t*-test. A *P*-value of < 0.05 was considered statistically significant.

E Volcano plot of mitochondrial protein changes in *TMBIM5*$^{-/-}$ HeLa cells when compared to WT HeLa cells. Significantly enriched mitochondrial complex I proteins at an FDR cutoff of 0.05 are colored in red. $n = 6$ independent experiments. *P*-values were calculated from a two-sided *t*-test followed by a permutation-based FDR controlling to 0.05. See also Dataset EV2.

F Volcano plot of mitochondrial protein changes in *TMBIM5*$^{-/-}$ HEK293T cells when compared to WT HEK293T cells. Significantly enriched mitochondrial complex I proteins at an FDR cutoff of 0.05 are colored in red. $n = 5$ independent experiments. *P*-values were calculated from a two-sided *t*-test followed by a permutation-based FDR controlling to 0.05. See also Dataset EV3.

G Scatter plot comparing the STRING enrichment score versus the −log10 transformed false discovery rate (color encoded). The number of genes mapped to the enriched GOBP term is encoded by size.

H mtDNA levels from HeLa cells assessed by qPCR amplification of mitochondrial CYTB ($n = 4$ independent experiments, mean ± SD).

© 2022 The Authors

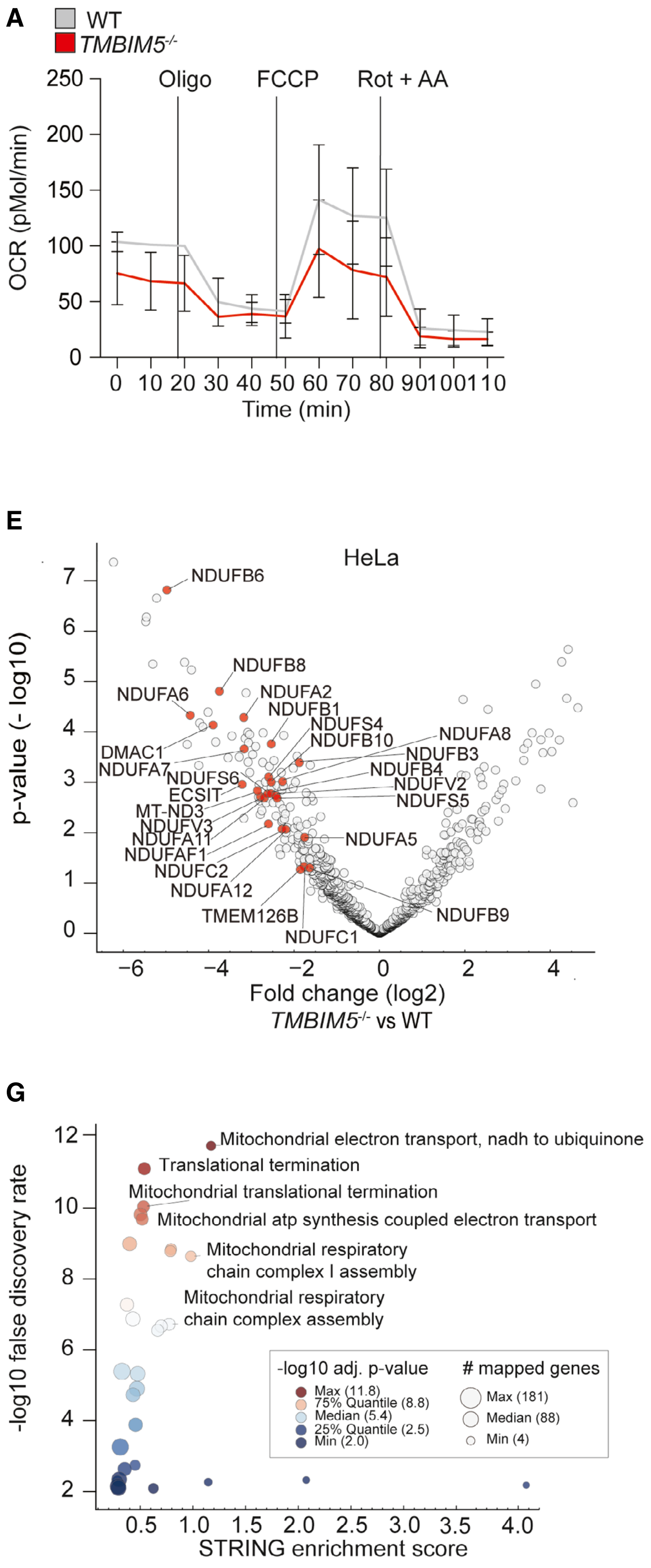
A
WT
TMBIM5-/-
Oligo
FCCP
Rot + AA
OCR (pMol/min)
Time (min)
B
Basal respiration
p=0.02
C
Maximal respiration
D
ATP production
p=0.004
E
HeLa
NDUFB6
NDUFB8
NDUFA6
NDUFA2
NDUFB1
NDUFS4
NDUFB10
NDUFA8
DMAC1
NDUFA7
NDUFB3
NDUFS6
NDUFB4
ECSIT
NDUFV2
NDUFS5
MT-ND3
NDUFV3
NDUFA11
NDUFA5
NDUFAF1
NDUFC2
NDUFA12
TMEM126B
NDUFB9
NDUFC1
p-value (- log10)
Fold change (log2)
TMBIM5-/- vs WT
F
HEK293T
NDUFA4
NDUFA6
NDUFS6
NDUFA7
NDUFA1
G
Mitochondrial electron transport, nadh to ubiquinone
Translational termination
Mitochondrial translational termination
Mitochondrial atp synthesis coupled electron transport
Mitochondrial respiratory chain complex I assembly
Mitochondrial respiratory chain complex assembly
-log10 adj. p-value
Max (11.8)
75% Quantile (8.8)
Median (5.4)
25% Quantile (2.5)
Min (2.0)
mapped genes
Max (181)
Median (88)
Min (4)
-log10 false discovery rate
STRING enrichment score
H
mtDNA

Figure EV3.

Figure EV4. Loss of TMBIM5 promotes proteolysis by AFG3L2 (Related to Fig 5).

A Workflow of the complexome profiling experiment. Mitochondria were isolated from wildtype (WT) and *TMBIM5*$^{-/-}$ HEK293T cells and protein complexes were fractionated using Blue-native PAGE (BN–PAGE). Each lane was cut into 72 slices of equal size and digested using trypsin followed by LC–MS/MS analysis.

B Profile plot showing the iBAQ intensity of MitoCarta 3.0 complex I and complex III subunits across the measured gel slices (fractions) of a BN–PAGE upon fractionation of a mitochondrial fraction of HEK293T cells. Line represents the median of all individual proteins. The area indicates the interquantile range.

C Similar to (B), but for mitochondria from HEK293T *TMBIM5*$^{-/-}$ cells.

D Monitoring mitochondrial matrix pH using the synthetic pH sensor mtSypHer expressed in WT and *TMBIM5*$^{-/-}$ cells depleted of AFG3L2 when indicated.Four independent experiments; mean ± SD.

E BN–PAGE analysis of mitochondria isolated from WT and *TMBIM5*$^{-/-}$ HEK293T cells. Left panel, in-gel activity assay for respiratory complex I after fractionation by BN–PAGE of WT and *TMBIM5*$^{-/-}$ mitochondria depleted of AFG3L2 as indicated. Middle and right panel, immunoblot analysis of the BN–PAGE using antibodies directed against NDUFV1 and SDHA.

F SDS–PAGE analysis of the mitochondrial fraction analyzed in D.

Source data are available online for this figure.

 © 2022 The Authors

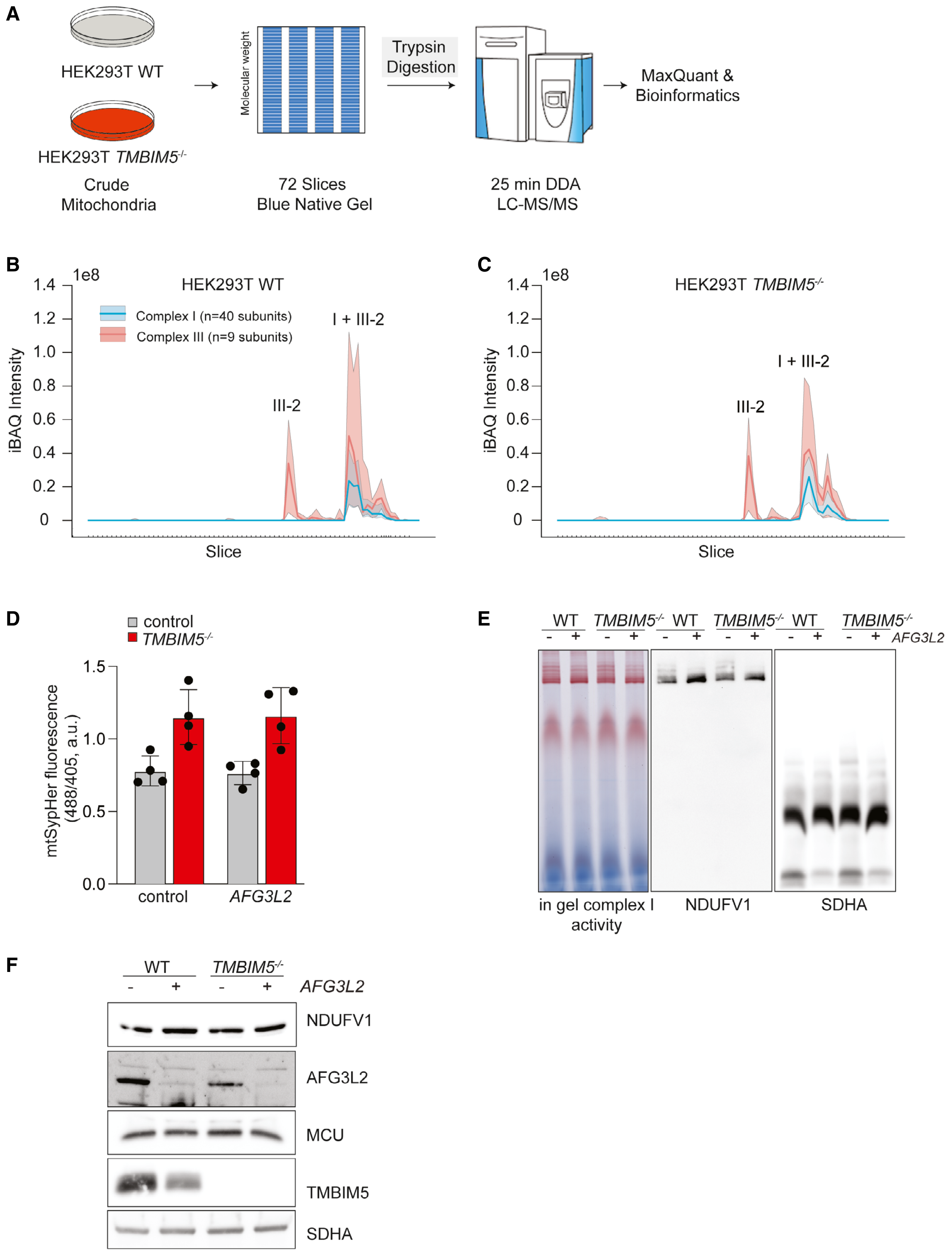
A
HEK293T WT
HEK293T TMBIM5-/-
Crude Mitochondria
Molecular weight
72 Slices
Blue Native Gel
Trypsin Digestion
25 min DDA
LC-MS/MS
MaxQuant & Bioinformatics
B
HEK293T WT
1e8
Complex I (n=40 subunits)
Complex III (n=9 subunits)
III-2
I + III-2
iBAQ Intensity
Slice
C
HEK293T TMBIM5-/-
D
control
TMBIM5-/-
mtSypHer fluorescence (488/405, a.u.)
control
AFG3L2
E
WT
TMBIM5-/-
AFG3L2
in gel complex I activity
NDUFV1
SDHA
F
WT
TMBIM5-/-
AFG3L2
NDUFV1
AFG3L2
MCU
TMBIM5
SDHA

Figure EV4.

Figure EV5. Hyperpolarization induces degradation of TMBIM5 allowing AFG3L2-mediated remodeling of the mitochondrial proteome (Related to Fig 6).

A Representative mtSypHer traces of HeLa cells treated with oligomycin (10 μM) or FCCP (10 μM) at the indicated timepoints. Ratios of the absorbance at 488 nm and 405 nm are shown in arbitrary units (a.u.).

B Quantification of *TMBIM5* mRNA monitored by RT–qPCR of HeLa cells treated as in Fig 6C. $n = 3$ independent experiments; mean ± SD.

C Quantification of Fig 6C. WT HeLa cells treated with the indicated drugs for 16 h. antimycin A (A; 10 μM), piericidin (P; 10 μM), and oligomycin (O; 10 μM). $n = 7$ independent experiments; mean ± SD.

D Bar graph displaying log2 fold changes between transcript (mRNA) (Fig EV5C) and protein (Fig EV5B) changes for TMBIM5 in cells treated as indicated.

E Representative immunoblot of WT HeLa cells transfected of wildtype (WT) and *AFG3L2*$^{-/-}$ cells. Samples were treated with the protein synthesis inhibitor emetine (10 μg/ml) and oligomycin (10 μM) and analyzed at the indicated time points ($n = 3$ independent experiments).

F Quantification of experiments shown in (E). Data are shown as mean ± SD.

G Mitochondrial membrane potential was monitored with TMRM in WT HeLa cells transiently transfected with scrambled siRNA (control) and siRNA targeting *AFG3L2* for 48 h. Fluorescent intensity was calculated relative to the value in the presence of CCCP (15 μM). Values are expressed as mean ± SD relative to control. Each measurement was performed in triplicate from four different preparations.

Source data are available online for this figure.

 © 2022 The Authors

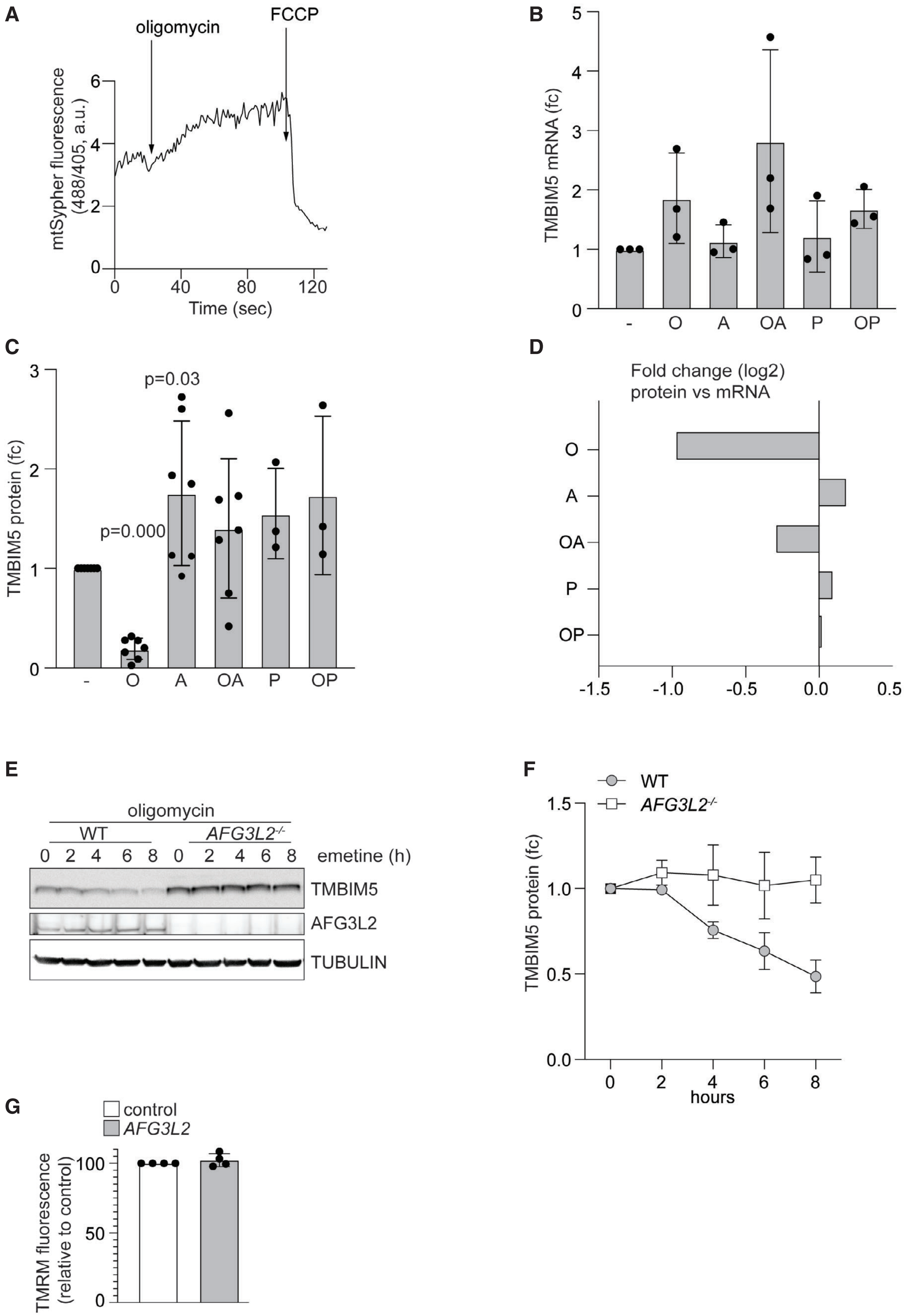
A
oligomycin
FCCP
mtSypher fluorescence (488/405, a.u.)
Time (sec)
B
TMBIM5 mRNA (fc)
- O A OA P OP
C
TMBIM5 protein (fc)
p=0.03
p=0.000
- O A OA P OP
D
Fold change (log2) protein vs mRNA
O
A
OA
P
OP
E
oligomycin
WT
AFG3L2-/-
0 2 4 6 8 0 2 4 6 8 emetine (h)
TMBIM5
AFG3L2
TUBULIN
F
WT
AFG3L2-/-
TMBIM5 protein (fc)
hours
G
control
AFG3L2
TMRM fluorescence (relative to control)

Figure EV5.
