# Supplementary figures and images for: Regulation of mitochondrial proteostasis by the proton gradient
Source: EMBO J. 2022 Aug 1;41(16):e110476. doi: 10.15252/embj.2021110476 (PMC9379554; doi:10.15252/embj.2021110476)

Fig. EV4 E

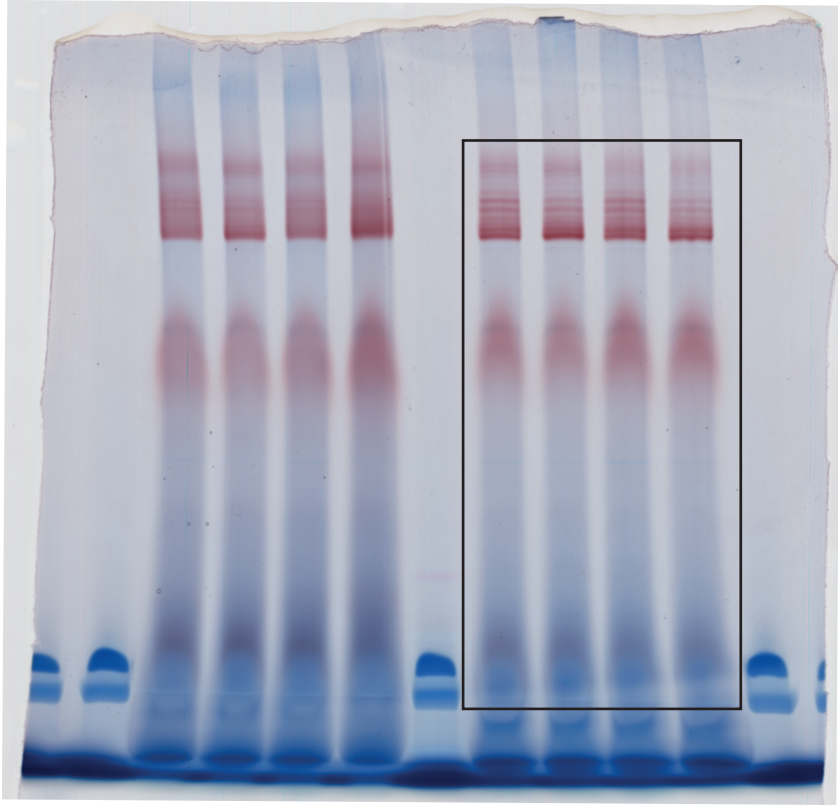

enzyme activity assay Complex I

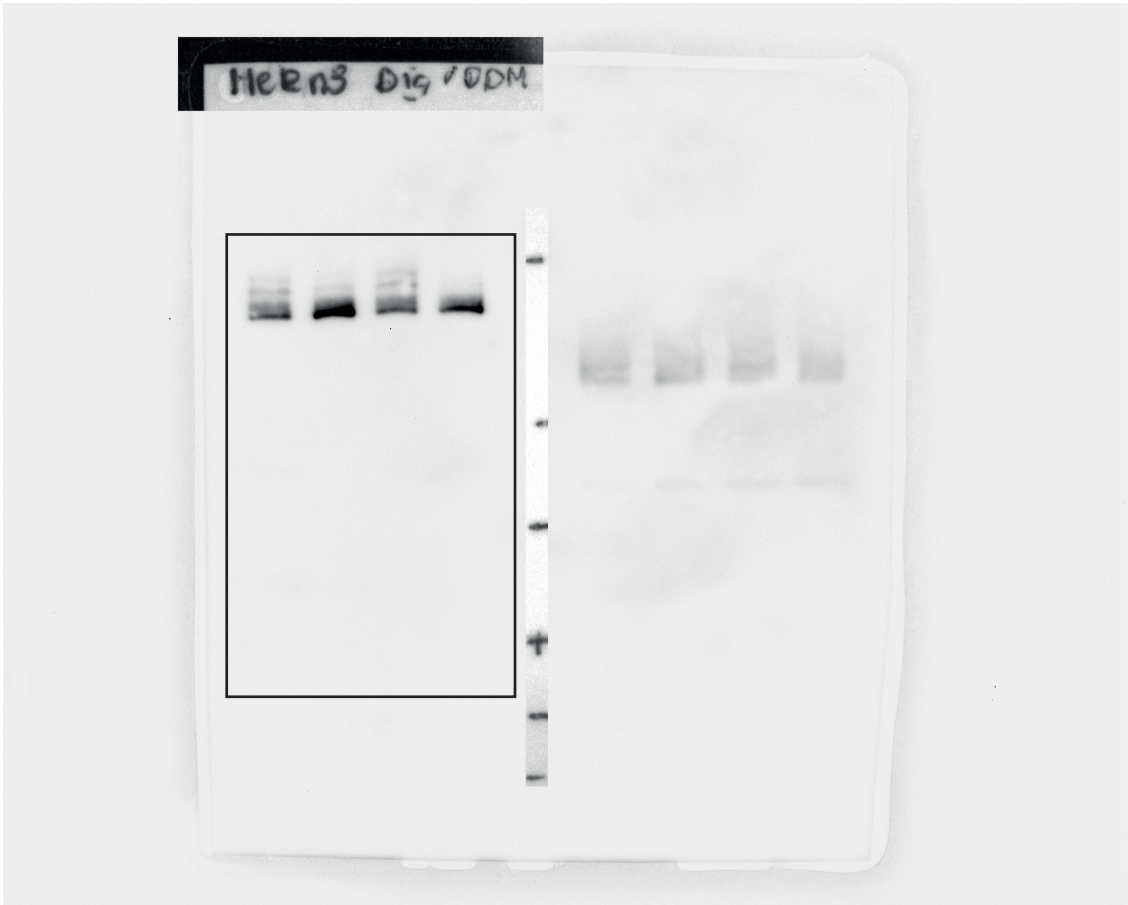

NDUFV1

HeRn3 Dig / ODM

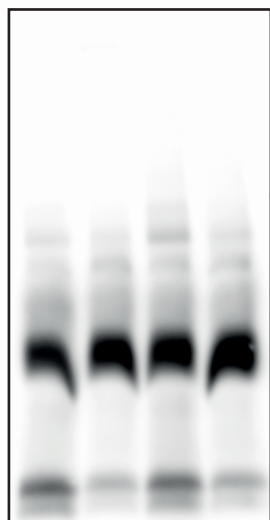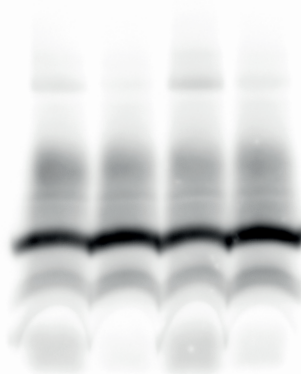

SDHA

Fig. EV4 F

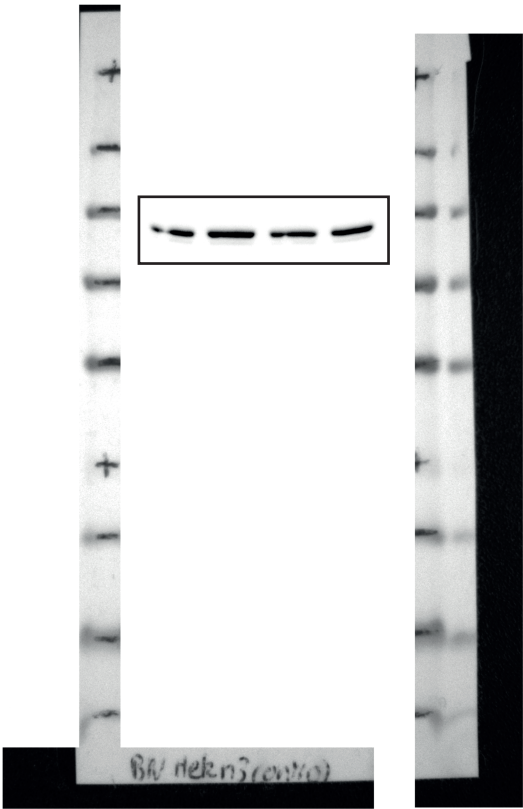

NDUFV1

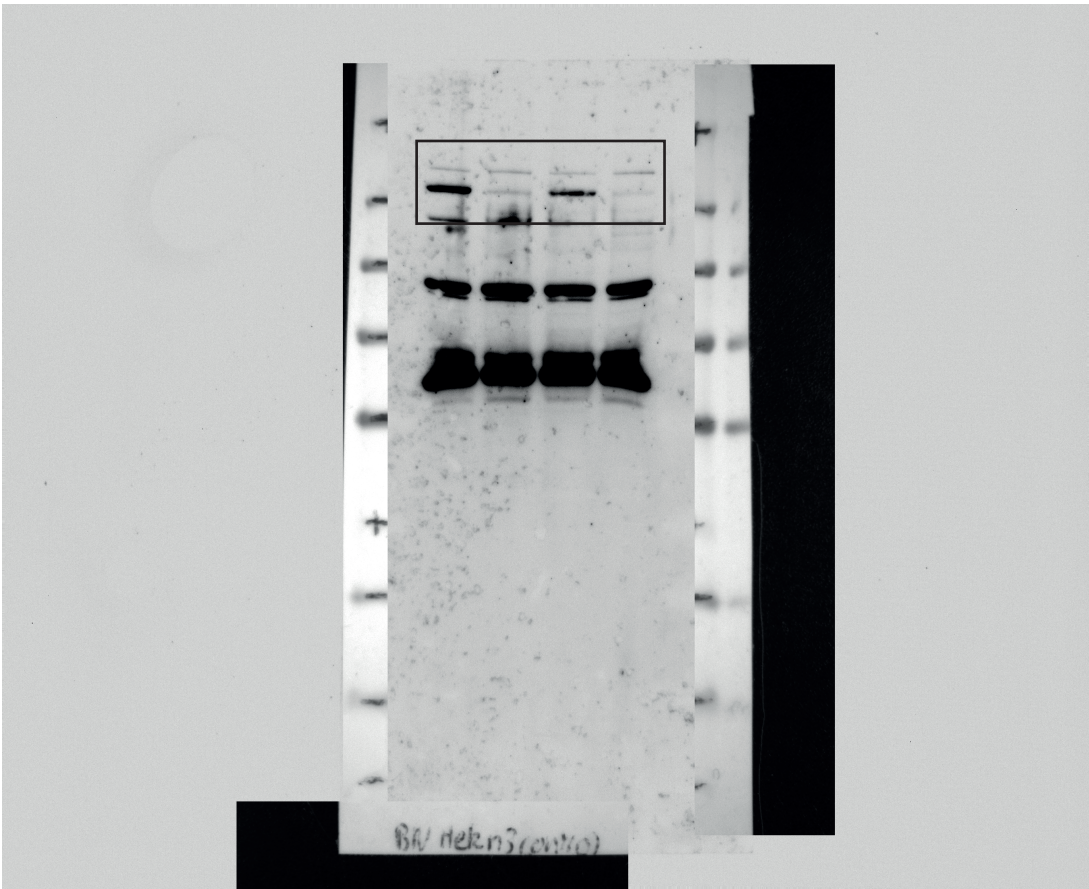

AFG3L2

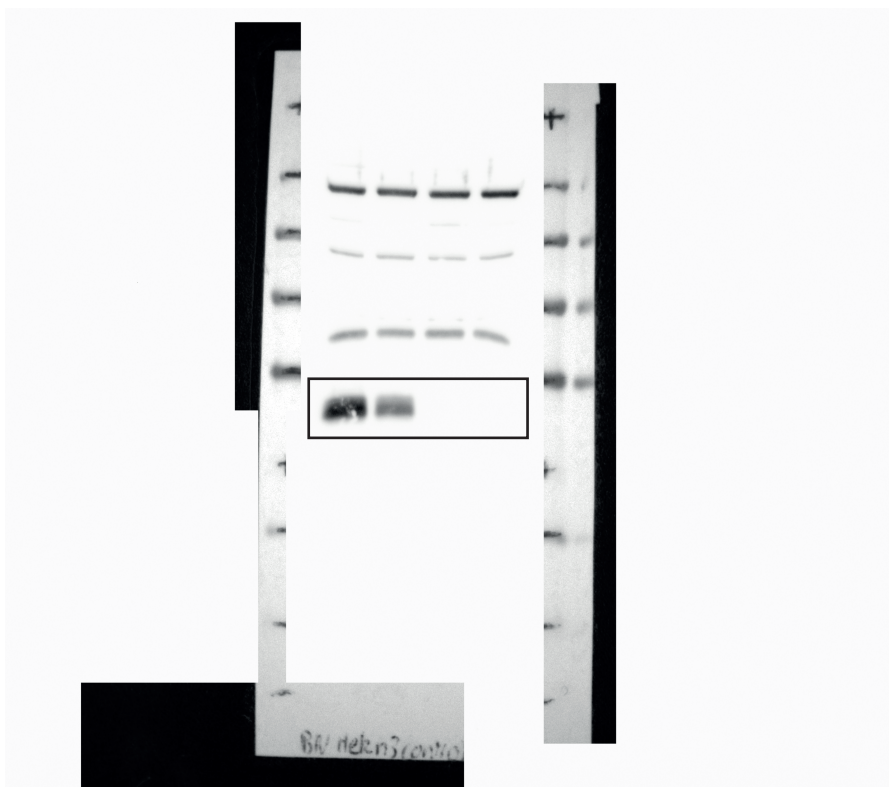

TMBIM5

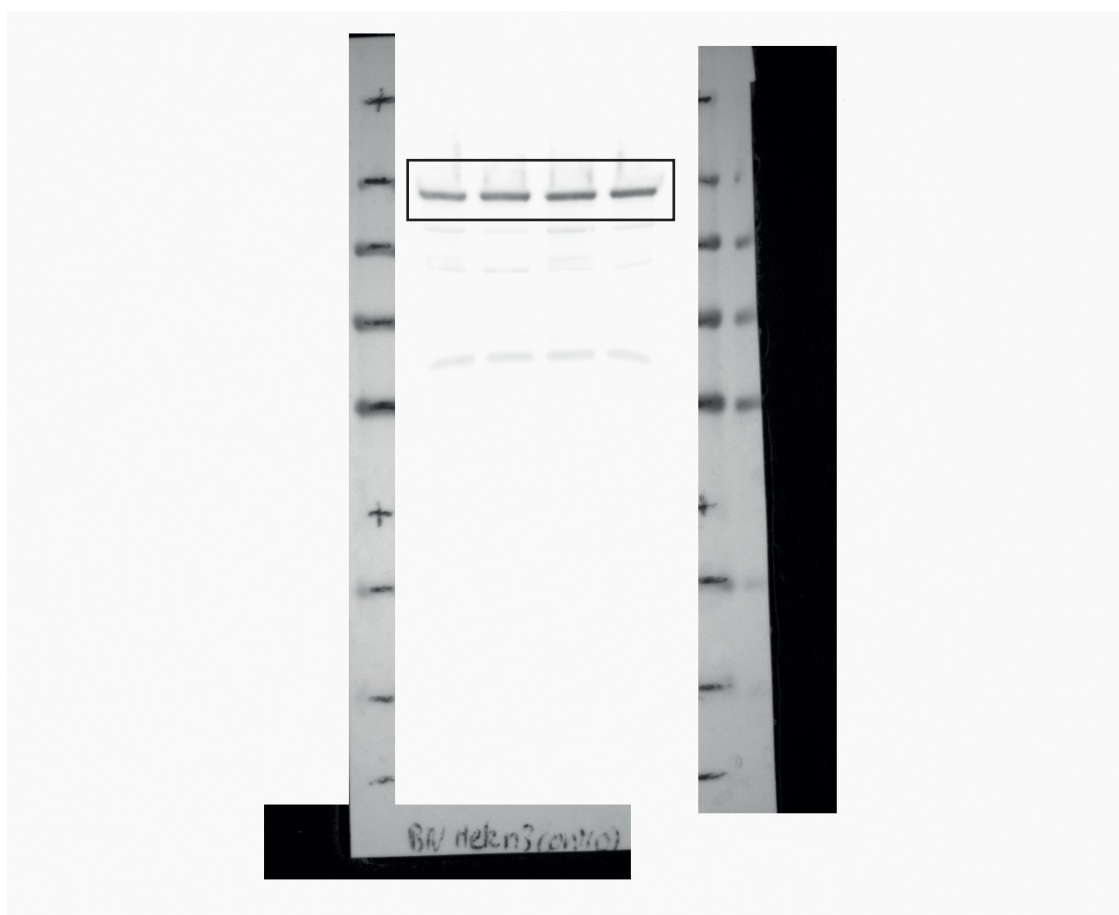

SDHA

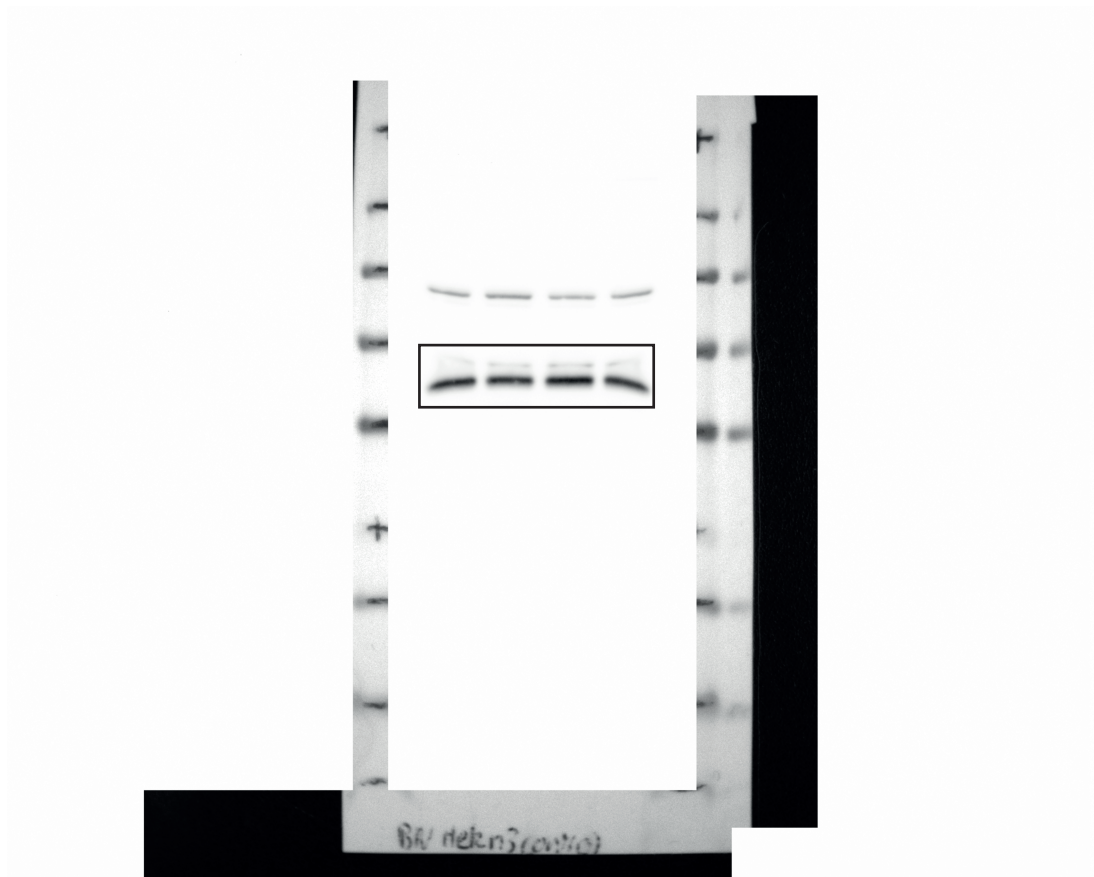

MCU

Supplement: Supplementary file 10 — Source Data for Expanded View [file EMBJ-41-e110476-s010.zip › Source_data_uncropped_images_FigureEV4.pdf]

Fig. EV5 E

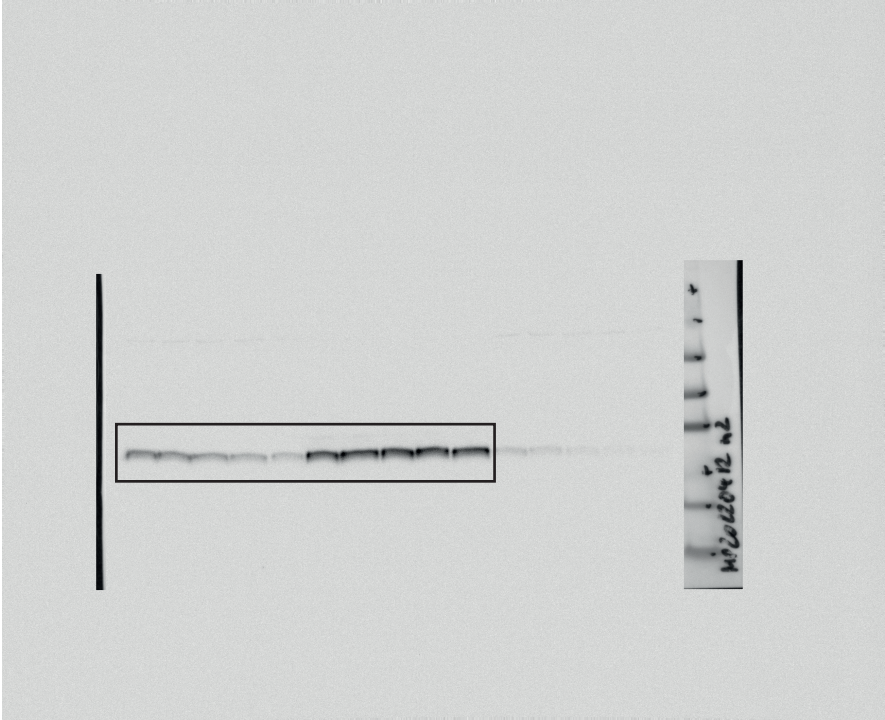

TMBIM5

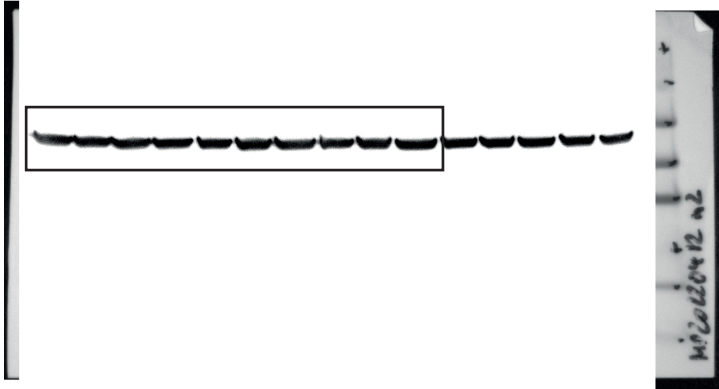

Tubulin

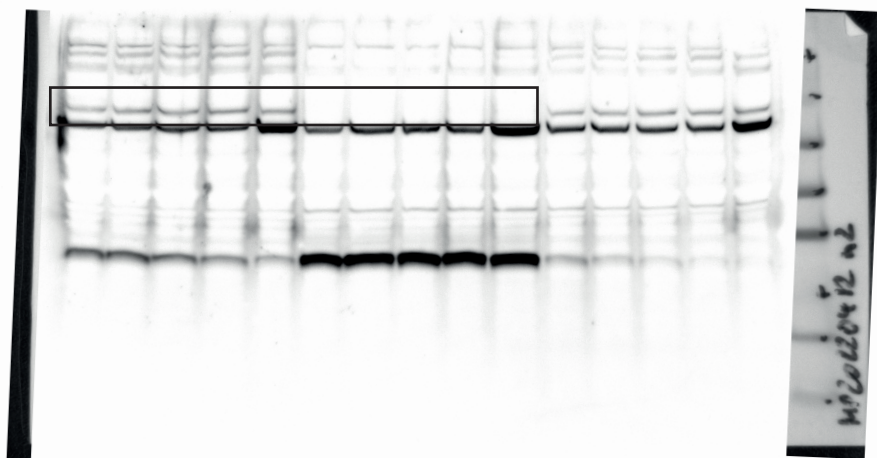

AFG3L2

Supplement: Supplementary file 10 — Source Data for Expanded View [file EMBJ-41-e110476-s010.zip › Source_data_uncropped_images_FigureEV5.pdf]

Figure EV1A

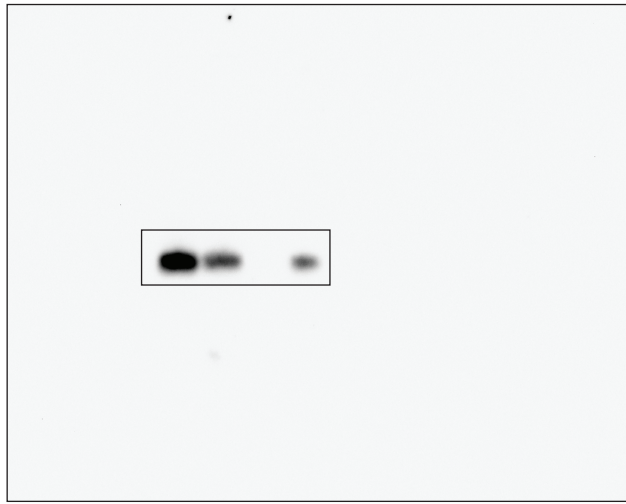

TMBIM5

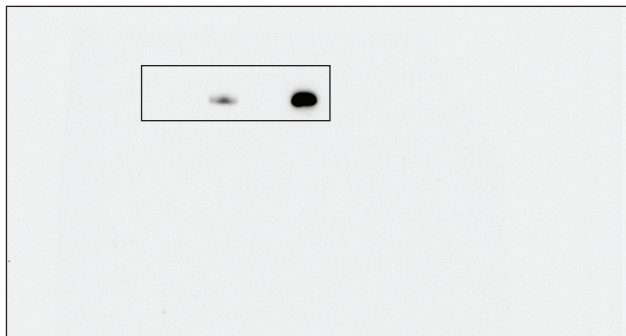

AFG3L2

Figure EV1B

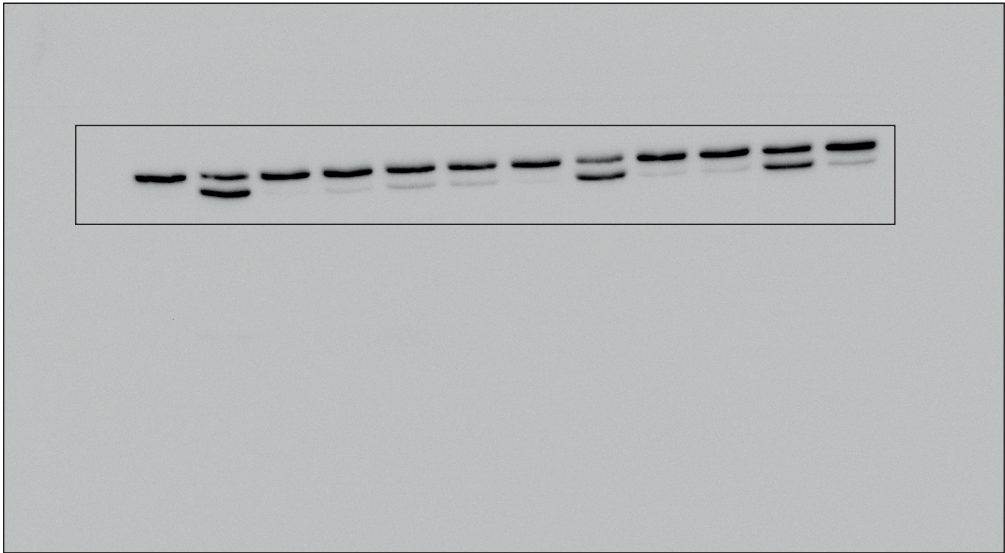

PARP

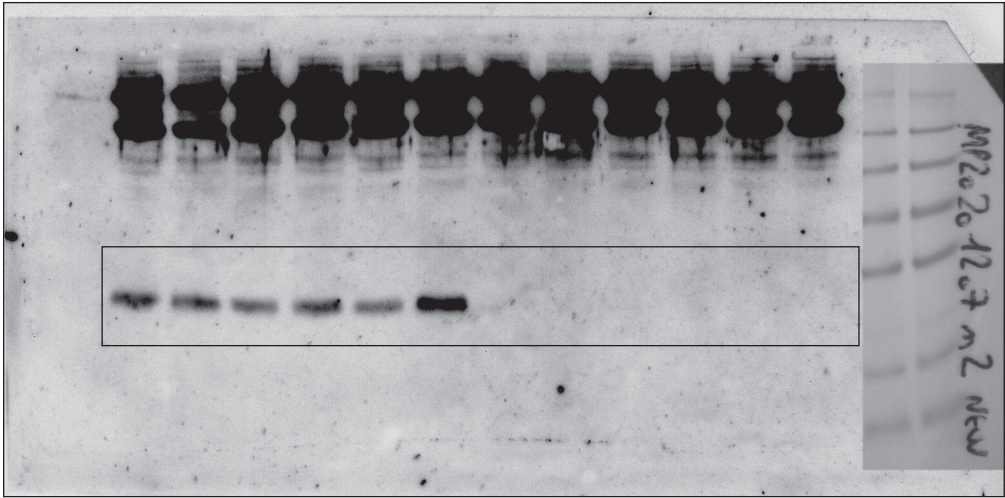

TMBIM5

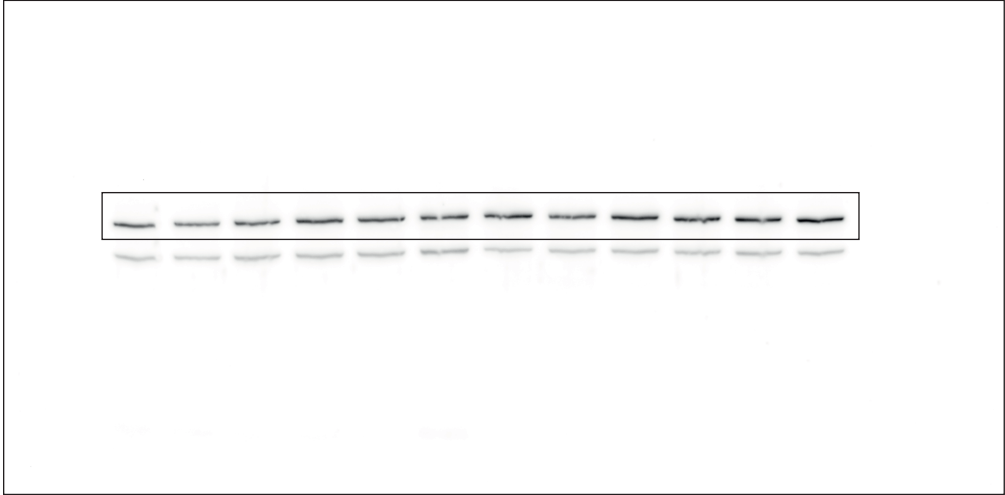

VINCULIN

Supplement: Supplementary file 10 — Source Data for Expanded View [file EMBJ-41-e110476-s010.zip › Source_data_uncropped_images_FigureEV1.pdf]

Figure 1B

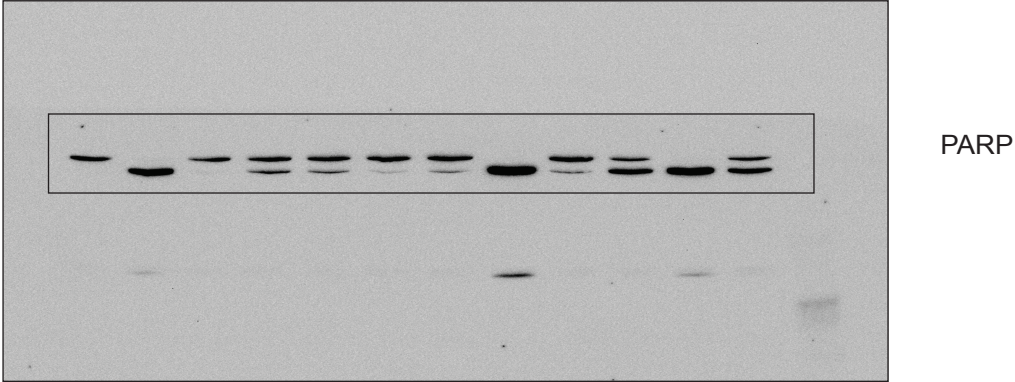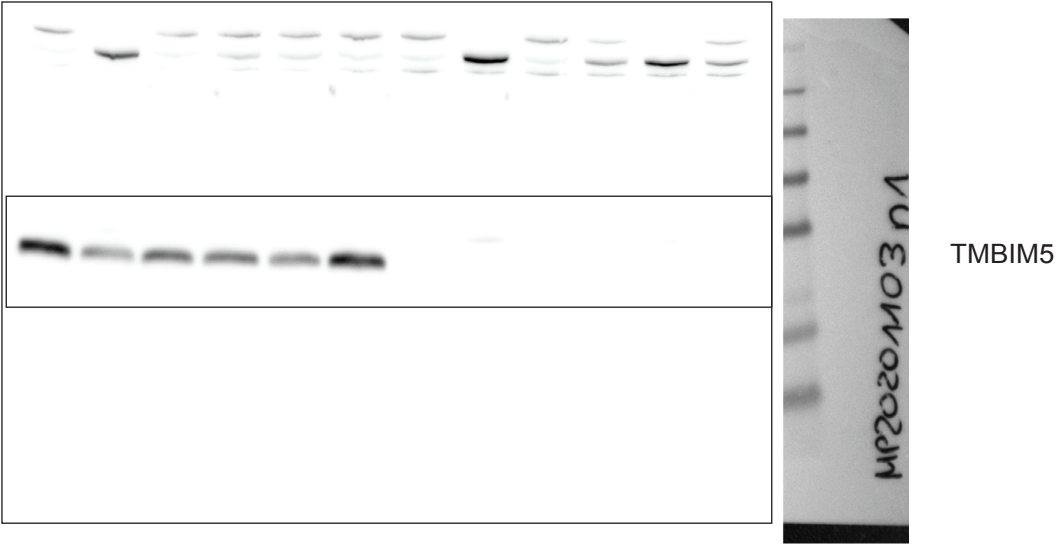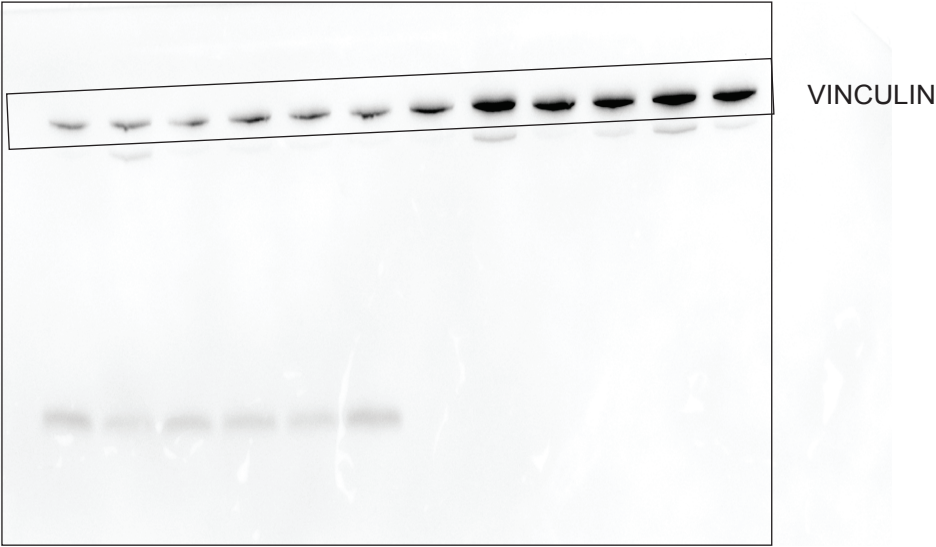

Figure 1C

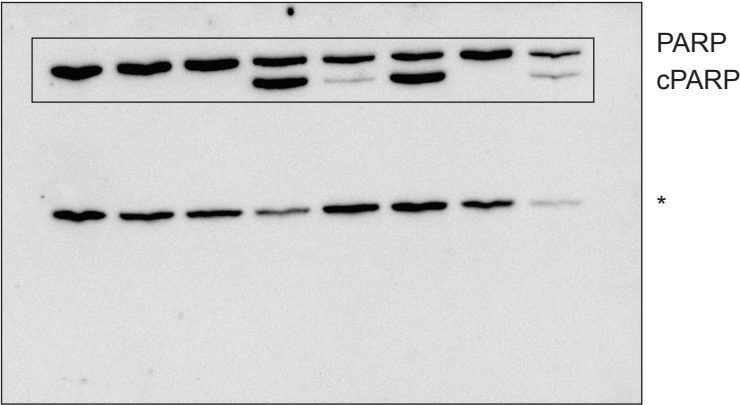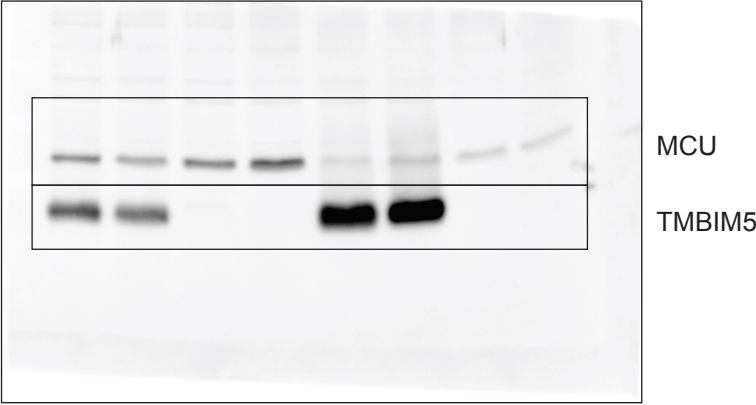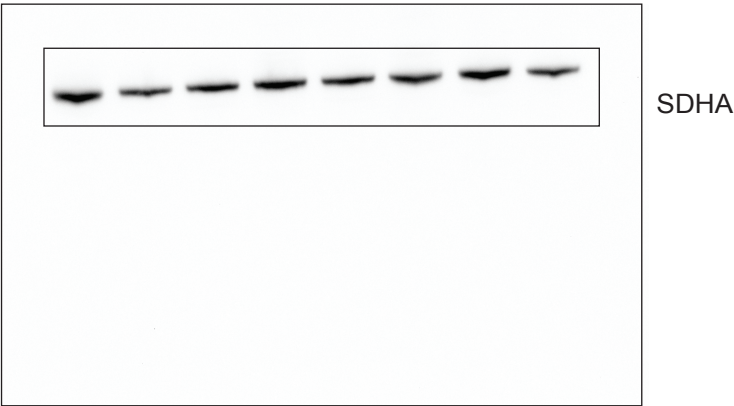

Supplement: Supplementary file 11 — Source Data for Figure 1 [file EMBJ-41-e110476-s004.pdf]

Figure 2E

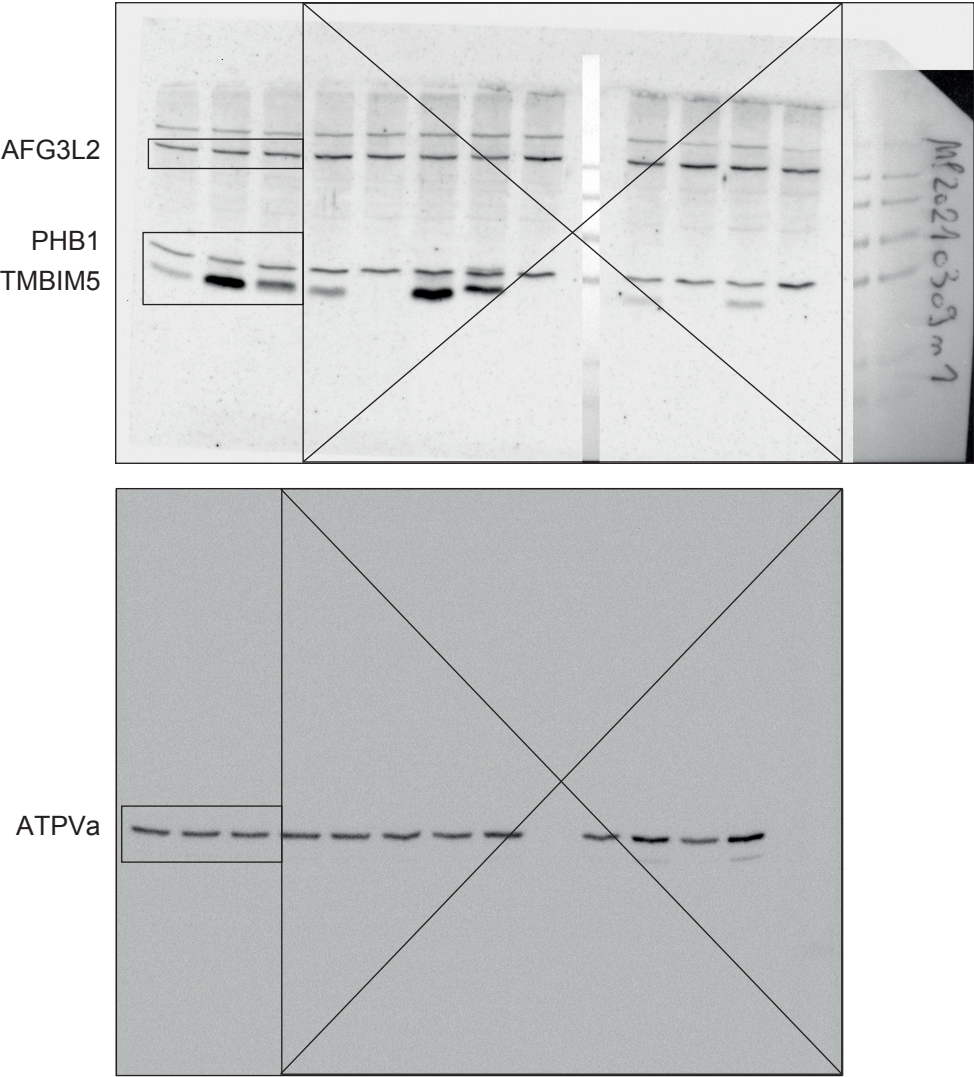

Supplement: Supplementary file 12 — Source Data for Figure 2 [file EMBJ-41-e110476-s009.pdf]

Figure 5C

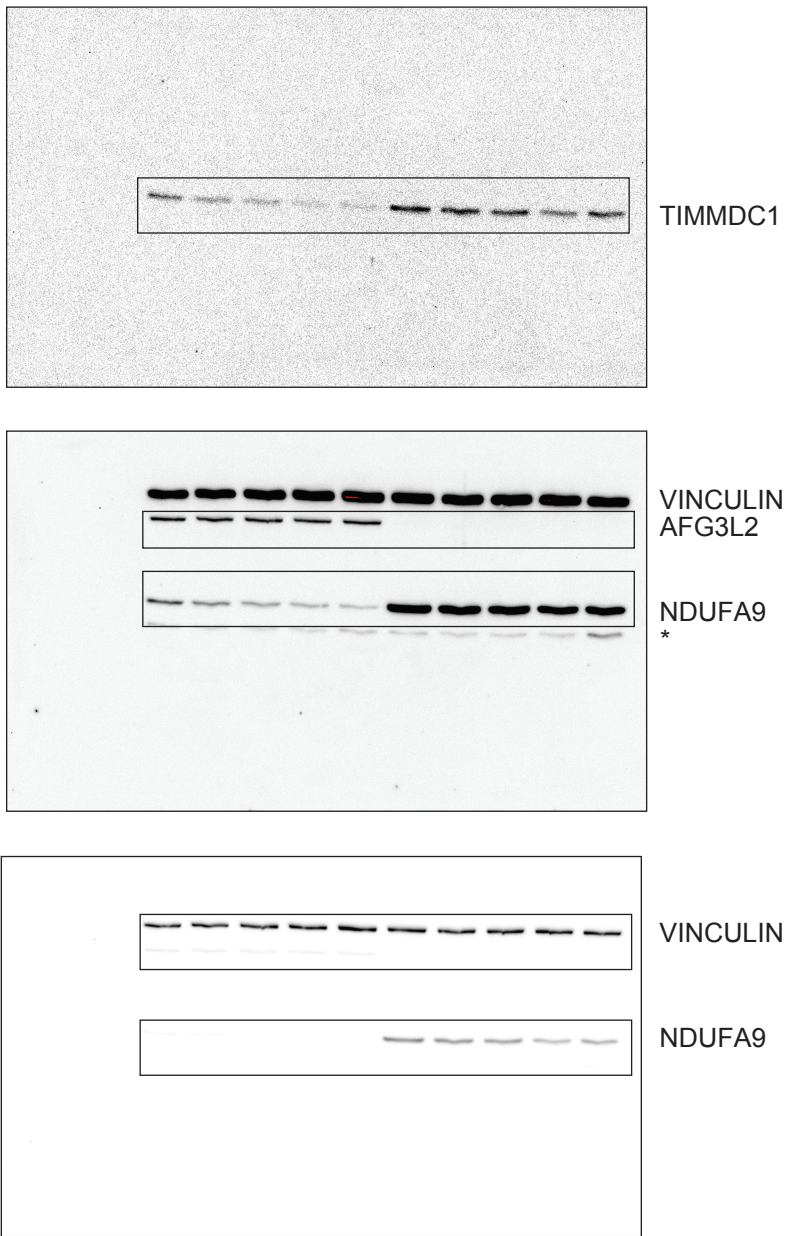

Supplement: Supplementary file 13 — Source Data for Figure 5 [file EMBJ-41-e110476-s006.pdf]

Figure 6C

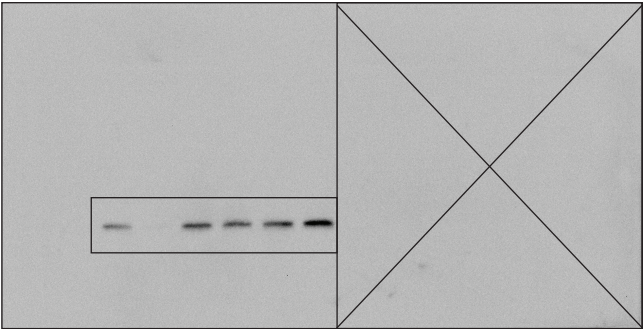

TIMBIM5

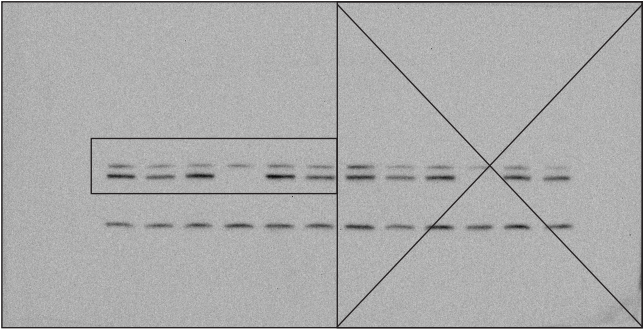

OMA1

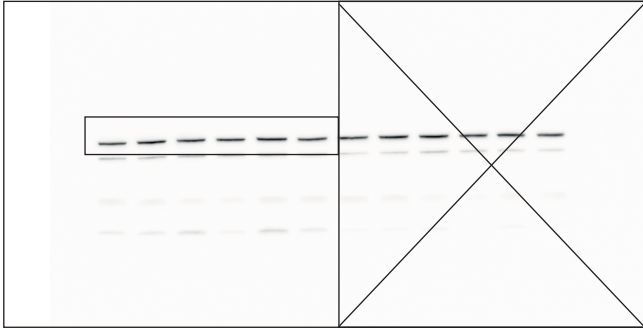

CV-ATP5A

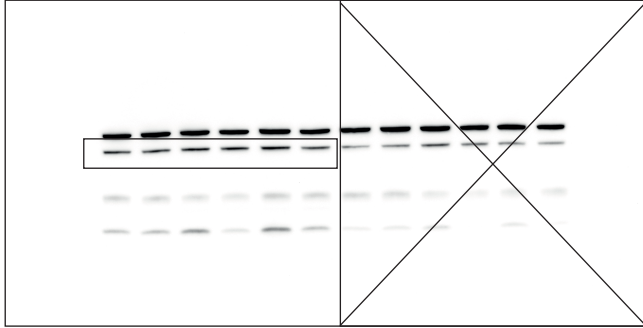

CIII-UQCRC2

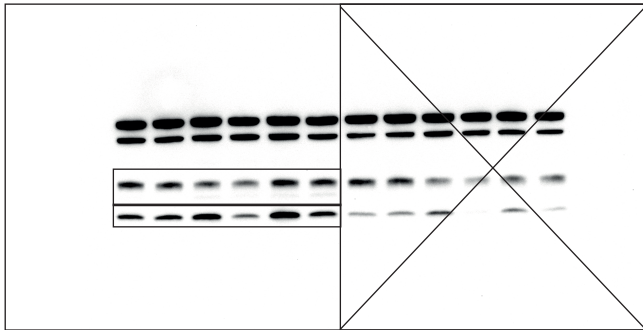

CII-SDHB  
CI-NDUFAB8

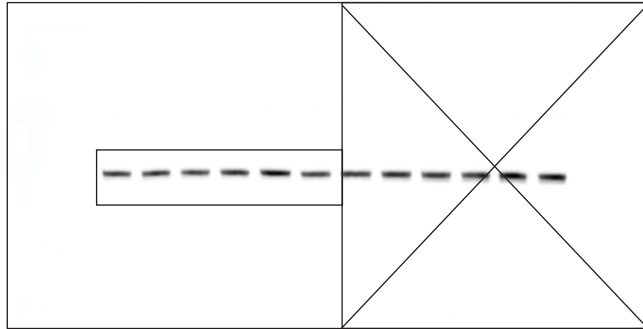

TUBULIN

Figure 6 D

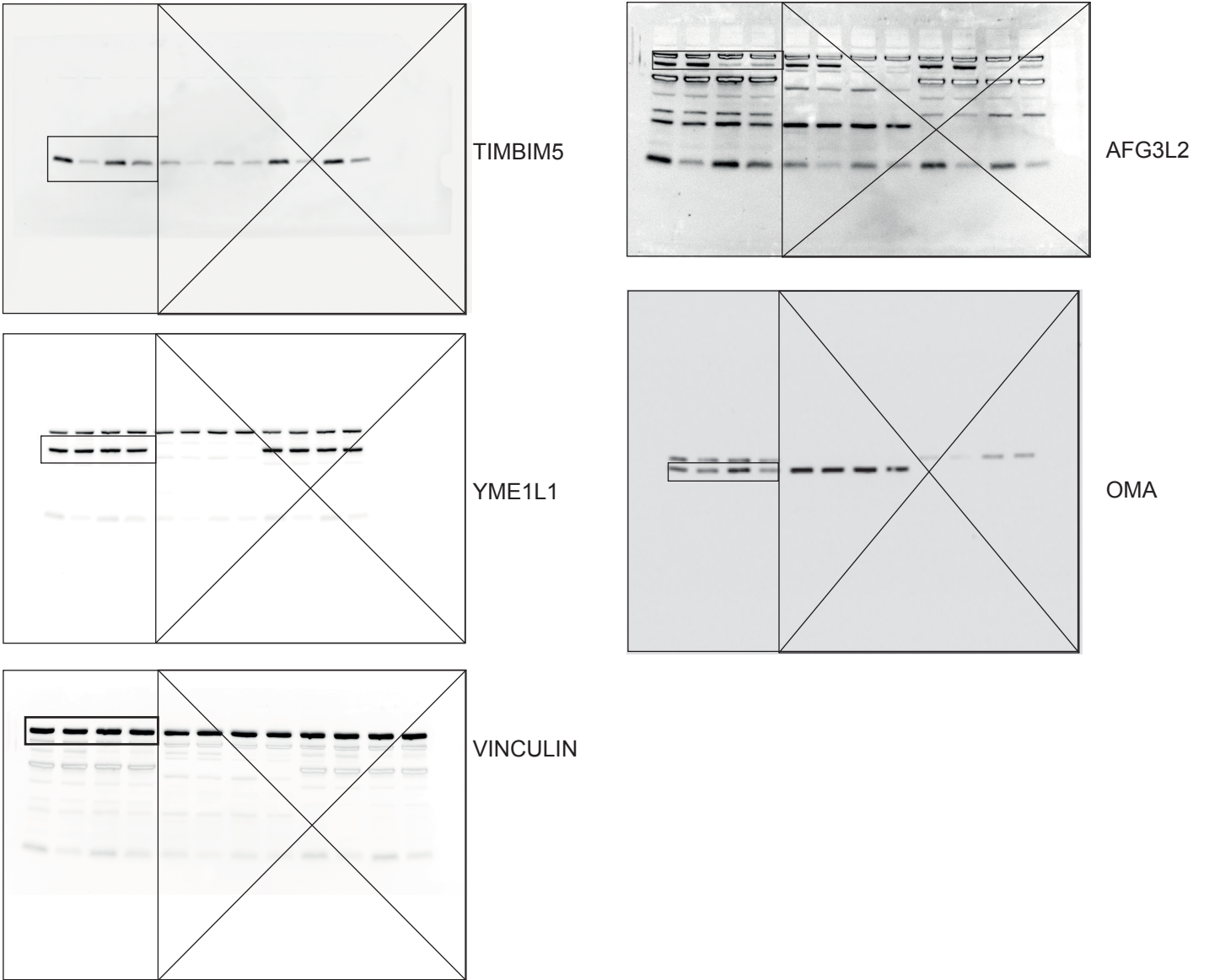

Supplement: Supplementary file 14 — Source Data for Figure 6 [file EMBJ-41-e110476-s011.pdf]
