# Supplementary material for: Regulation of mitochondrial proteostasis by the proton gradient
Source: EMBO J. 2022 Aug 1;41(16):e110476. doi: 10.15252/embj.2021110476 (PMC9379554; doi:10.15252/embj.2021110476)
Supplement: Supplementary file 15 [file EMBJ-41-e110476-s002.pdf]

THE EMBO JOURNAL

Regulation of mitochondrial proteostasis by the proton gradient

Maria Patron, Daryna Tarasenko, Hendrik Nolte, Mausumi Gosh, Yohsuke Ohba, Yvonne Lasarzewski, Zeinab Ahmadi, Alfredo Cabrera-Orefice, Akinori Eyiama, Tim Kellermann, Elena Rugarli, Ulrich Brandt, Michael Meinecke, Lara Kroczek and Thomas Langer
DOI: 10.15252/embj.2021110476

Corresponding author: Thomas Langer (Langer@age.mpg.de)

Review Timeline:

Submission Date:	17th Dec 21
Editorial Decision:	21st Jan 22
Revision Received:	3rd May 22
Editorial Decision:	18th May 22
Revision Received:	29th Jun 22
Accepted:	1st Jul 22

Editor: Daniel Klimmeck

Transaction Report:

(Note: With the exception of the correction of typographical or spelling errors that could be a source of ambiguity, letters and reports are not edited. Depending on transfer agreements, referee reports obtained elsewhere may or may not be included in this compilation. Referee reports are anonymous unless the Referee chooses to sign their reports.)

1st Editorial Decision **21st Jan 2022**

Dear Thomas,

Thank you again for the submission of your manuscript (EMBOJ-2021-110476) to The EMBO Journal. Please accept my apologies for the delay with the peer-review of your work due to protracted referee input and detailed discussions in the team. Your study has been sent to three referees, and we have received reports from all of them, which I enclose below.

As you will see, the referees acknowledge the potential interest and value of your results, although they also express a number of important concerns, which need to be addressed before they can be supportive of publication at the EMBO Journal. In more detail, referee #2 finds that the claim on direct and causal interaction of TMBIB5 and i-AAA is not convincingly supported by the current data and asks you amend the study in that direction (ref#2, pt. 1). In line, reviewer #1 requests complementary experiments to strengthen a causal role of TMBIB5 and disambiguate control vs assembly factor roles (ref#1, pts.1,2). Referee #3, a proteomics expert, points out that the methods workflow and data processing details are not conclusively documented and the full datasets should be made available (ref#3, pts, 2,3; see also ref#2, pt.2). Finally, all three referees list a number of additional minor issues related to data presentation and illustration of the model as well as typos that would have to be addressed to achieve the level of robustness needed for The EMBO Journal.

Given the referees' overall positive recommendations and detailed comments, I would like to invite you to submit a revised version of the manuscript, addressing the issues raised. I should add that it is EMBO Journal policy to allow only a single round of revision, and acceptance of your manuscript will therefore depend on the completeness of your responses in this revised version.

We generally allow three months as standard revision time. As a matter of policy, competing manuscripts published during this period will not negatively impact on our assessment of the conceptual advance presented by your study. However, we request that you contact the editor as soon as possible upon publication of any related work, to discuss how to proceed. Should you foresee a problem in meeting this three-month deadline, please let us know in advance and we may be able to grant an extension.

When submitting your revised manuscript, please carefully review the instructions below.

Thank you for the opportunity to consider your work for publication.
I look forward to your revision.

Best regards,

Daniel

Daniel Klimmeck, PhD
Senior Editor
The EMBO Journal

Instruction for the preparation of your revised manuscript:

1) a .docx formatted version of the manuscript text (including legends for main figures, EV figures and tables). Please make sure that the changes are highlighted to be clearly visible.

2) individual production quality figure files as .eps, .tif, .jpg (one file per figure).

3) a .docx formatted letter INCLUDING the reviewers' reports and your detailed point-by-point response to their comments. As part of the EMBO Press transparent editorial process, the point-by-point response is part of the Review Process File (RPF), which will be published alongside your paper.

4) a complete author checklist, which you can download from our author guidelines (https://wol-prod-cdn.literatumonline.com/pb-assets/embo-site/Author Checklist%20-%20EMBO%20J-1561436015657.xlsx). Please insert information in the checklist that is also reflected in the manuscript. The completed author checklist will also be part of the RPF.

5) Please note that all corresponding authors are required to supply an ORCID ID for their name upon submission of a revised manuscript.

6) It is mandatory to include a 'Data Availability' section after the Materials and Methods. Before submitting your revision, primary datasets produced in this study need to be deposited in an appropriate public database, and the accession numbers and

database listed under 'Data Availability'. Please remember to provide a reviewer password if the datasets are not yet public (see https://www.embopress.org/page/journal/14602075/authorguide#datadeposition).
In case you have no data that requires deposition in a public database, please state so in this section. Note that the Data Availability Section is restricted to new primary data that are part of this study.
*** Note - All links should resolve to a page where the data can be accessed. ***

7) Our journal encourages inclusion of *data citations in the reference list* to directly cite datasets that were re-used and obtained from public databases. Data citations in the article text are distinct from normal bibliographical citations and should directly link to the database records from which the data can be accessed. In the main text, data citations are formatted as follows: "Data ref: Smith et al, 2001" or "Data ref: NCBI Sequence Read Archive PRJNA342805, 2017". In the Reference list, data citations must be labeled with "[DATASET]". A data reference must provide the database name, accession number/identifiers and a resolvable link to the landing page from which the data can be accessed at the end of the reference. Further instructions are available at .

8) We would also encourage you to include the source data for figure panels that show essential data. Numerical data can be provided as individual .xls or .csv files (including a tab describing the data). For 'blots' or microscopy, uncropped images should be submitted (using a zip archive or a single pdf per main figure if multiple images need to be supplied for one panel). Additional information on source data and instruction on how to label the files are available at .

9) We replaced Supplementary Information with Expanded View (EV) Figures and Tables that are collapsible/expandable online (see examples in https://www.embopress.org/doi/10.15252/embj.201695874). A maximum of 5 EV Figures can be typeset. EV Figures should be cited as 'Figure EV1, Figure EV2" etc. in the text and their respective legends should be included in the main text after the legends of regular figures.

- For the figures that you do NOT wish to display as Expanded View figures, they should be bundled together with their legends in a single PDF file called *Appendix*, which should start with a short Table of Content. Appendix figures should be referred to in the main text as: "Appendix Figure S1, Appendix Figure S2" etc. See detailed instructions regarding expanded view here: .

- Additional Tables/Datasets should be labelled and referred to as Table EV1, Dataset EV1, etc. Legends have to be provided in a separate tab in case of .xls files. Alternatively, the legend can be supplied as a separate text file (README) and zipped together with the Table/Dataset file.

10) When assembling figures, please refer to our figure preparation guideline in order to ensure proper formatting and readability in print as well as on screen:
http://bit.ly/EMBOPressFigurePreparationGuideline

Please remember: Digital image enhancement is acceptable practice, as long as it accurately represents the original data and conforms to community standards. If a figure has been subjected to significant electronic manipulation, this must be noted in the figure legend or in the 'Materials and Methods' section. The editors reserve the right to request original versions of figures and the original images that were used to assemble the figure.

11) For data quantification: please specify the name of the statistical test used to generate error bars and P values, the number (n) of independent experiments (specify technical or biological replicates) underlying each data point and the test used to calculate p-values in each figure legend. The figure legends should contain a basic description of n, P and the test applied. Graphs must include a description of the bars and the error bars (s.d., s.e.m.).

Further information is available in our Guide For Authors: https://www.embopress.org/page/journal/14602075/authorguide

We realize that it is difficult to revise to a specific deadline. In the interest of protecting the conceptual advance provided by the work, we recommend a revision within 3 months (21st Apr 2022). Please discuss the revision progress ahead of this time with the editor if you require more time to complete the revisions. Use the link below to submit your revision:

Link Not Available

Referee #1:

The cellular proteome is shaped by the interplay of protein synthesis and protein degradation. In this study, the authors identified the mitochondrial inner membrane protein TMBIM5 as a regulating interaction factor of the m-AAA protease, the major protease

of mitochondrial membrane proteins. TMBIM5 exhibits two activities: First, it serves as a Ca2+/H+ exchanger that couples the export of Ca2+ from mitochondria to the import of protons into mitochondria. The authors convincingly show that the loss of TMBIM5 leads to matrix alkalinization, mitochondrial hyperpolarization and the accumulation of Ca2+ within mitochondria, rendering cells sensitive to mitochondrial Ca2+ overload and cell death. In addition, TMBIM5 serves as binding factor of the m-AAA protease which chokes its proteolytic activity to prevent the m-AAA-mediated degradation of complex I proteins. Loss of TMBIM5 unleashes the m-AAA protease and promotes complex I turnover. Based on their data, the authors present an interesting model according to which TMBIM5 serves as an emergency valve which removes complex I once the levels of the membrane potential raise above a physiological range, thereby preventing hyperpolarization of the mitochondrial inner membrane.
This is an exciting study. It contains a wealth of interesting information, including a large number of proteomic analyses, all of which are well accessible from the excel data in the supplements. The study consists of three convincing parts describing the association of TIMBIM5 with AFG3L2, its function as a Ca2+/H+ exchanger and its relevance for complex I stability. Even though the model is compatible with the data shown, it also remains possible that TIMBIM5 plays some role in the biogenesis of complex I. According to this alternative 'assembly factor' model, its absence would also lead to an increased turnover of complex I subunits which would be prevented m-AAA protease activity is reduced. Such a function as a factor relevant for the assembly of membrane proteins was for example suggested for prohibitins, which - like TIMBIM5 - are AFG3L2 interactors. Even if some of the details of how TIMBIM5 influences complex I breakdown by the m-AAA protease remain unclear, this is an exciting study which will be of interest for a broad readership.

Main points
1. The authors should strengthen their evidence for a 'control factor function' rather than an 'assembly factor function' of TIMBIM5. For example, they show that upon knock down of AFG3L2, the levels of complex I subunits are partially restored. However, it remains unclear whether complex I is assembled and functional under these conditions. It should be straight forward to test this.
2. The authors introduce and analyze the TMBIM5(D294K D325K) mutant as a variant that lacks the pH-driven calcium pumping activity. I wonder why this interesting variant is not employed to test whether the transporter activity can be uncoupled from the role as inhibitor of the m-AAA activity. One would expect that expression of this mutant would constantly inhibit AFG3L2, thereby being insensitive to oligomycin. If possible, data on the analysis of this mutant should be added to Fig. 6.

Minor points
3. Page 6, middle: '(Figure 1D, Figure S1G and' should be '(Figure 1D, Figure S1G) and'
4. Fig. 1. The sizes of the different panels are not perfect. Fig. 1E is far too small and therefore the cytosolic cytochrome c is hardly visible (without strong blow-up of the figure). Other panels (C, D, F) could be shrunk if space is limiting.
5. Fig. 2A. A small sketch would help to explain what this figure shows. As many readers might not be familiar with mtRED-GECO, a graphical description would help here.
6. Fig. 2B. Is it possible that the change in pH is caused by the 'unleashed' m-AAA activity in the TMBIM5 mutants rather than by loss of TMBIM5-mediated proton pumping? Does knock down of AFG3L2 in TMBIM5 restore the pH of the matrix? According to the explanation of the authors, this would not be expected.

Referee #2:

This is an interesting paper, in which the authors identify TMBIM5 as a novel Ca2+/H+ exchanger of the inner mitochondrial membrane. They further demonstrate that TMBIM5 interacts with the m-AAA protease, that hyperpolarization triggers the loss of TMBIM5, and that the loss of TMBIM5 changes the composition of the mitochondrial proteome. They propose a model, in which TMBIM5 binds to m-AAA and is degraded by m-AAA upon hyperpolarization. Once TMBIM5 is degraded, m-AAA is released and then hydrolyzes multiple proteins in the inner membrane/matrix compartment. Thus, TMBIM5 integrates mitochondrial quality control with the bioenergetics status. The paper is novel, interesting, and significant. But there are two major issues and a number of minor issues that need to be addressed in a revision.

MAJOR ISSUES
1. The direct interaction between TMBIM5 and m-AAA is not sufficiently supported by the data. This should be acknowledged in the text of Results and Discussion. Specifically, the immunoprecipitation experiment cannot distinguish between a direct interaction between the two proteins and an indirect association mediated by other proteins. Also, the immunoprecipitation experiment should be described more extensively. It is not clear why digitonin was used for the solubilization of mitochondria because among commonly used detergents digitonin promotes indirect associations the most. Finally, in Fig 6D, the down-regulation of AFG3L2 did not stabilize TMBIM5 very much but AFG3L2-dependent degradation of TMBIM5 is crucial for the proposed model. In short, the weaknesses of those data should be acknowledged and the interpretation of those data adjusted accordingly.
2. The analysis of the proteomics data is confusing and inconsistent within the paper. Comparing acute changes induced by a drug (oligomycin) to chronic changes induced by protein deletion (TMBIM5-/-) does not make a lot of sense. Figures 6A and 6E are difficult to understand. Do the authors wish to conclude that oligomycin has an effect on wild-type cells but not on TMBIM5-/- cells ? If so, there should be an easier way to present that. Furthermore, the authors stress the point that complex I subunits are

specifically affected but at the same time write "...that the loss of TMBIM5 causes broad reshaping of the mitochondrial proteome...". These two statements are mutually exclusive. Finally, it is not clear to me how the data in Fig 5C were calculated. In 5C, the axes should be re-labeled to indicate what the numeric data represent. Overall, the proteomics section should be simplified and scrutinized for internal inconsistencies.

MINOR ISSUES
3. In Fig 2E, it should read "empty"
4. On p. 33, I mg/ml tetracycline seems very high
5. On p. 40, µm*ml-1 is not a unit of concentration
6. On p. 40, it is unclear what is meant by the "15 min swelling step" and what it is for
7. On p. 43, the abbreviation NMDG has not been defined
8. Does the topology of TMBIM5 (inside out vs right side out) matter in the in vitro experiments? Please explain.

Referee #3:

In this manuscript, the authors investigate the function and regulation of the m-AAA protease in mitochondria. They identify TMBIM5 as a Ca2+/H+ exchanger and a critical interactor of the m-AAA protease, regulating respiration and mitochondrial hyperpolarization. Functional interaction between m-AAA protease and m-AAA was shown during sustained hyperpolarization, resulting in degradation of TMBIM5 and subsequent remodeling of the mitochondrial proteome.

This is an intriguing and well-written paper, convincingly demonstrating how TMBIM5 functionally integrates mitochondrial Ca2+ signaling and energy status with remodeling of the mitochondrial proteome, to adjust cellular metabolism. Experiments were performed at a high technical level, including sophisticated proteomic experiments to investigate changes in protein turnover. I only have a few questions:

1. Fig 1a: What is the premise that candidate-binders should interact more prominently with catalytically inactive AFGL2? Or what was the negative control here: non-flagged mutated, or non-mutated (suggested by 'wt' as indicated in Fig 1)?

2. Fig 4i: The authors perform protein turnover experiments, claiming that 140 proteins turned over 'significantly' faster in the absence of TMBIM5 (p. 11). Since the effect-size seems to be marginal for most proteins (slightly off the diagonal in Fig 4i), based on 2 (?) replicates, I wonder how significance was determined. There are two major points here: i) such experiments are typically prone to experimental variability resulting in deviations in half-life estimates, and ii) if the authors used conventional models for curve fitting, assuming exponential decay (no details are provided), this requires cells to be in steady state - which they are not. In addition, the authors introduce perturbations (KO of TMBIM5) that impact cellular metabolism/proliferation, formally making comparisons in half-life between conditions highly problematic. Therefore, more details should be provided how the data were treated/interpreted to arrive at the claims that are made.

3. Related to the point above: the authors state that they determined turnover rates for 348 mitochondrial proteins (p.11), however their data must include many more (extra-mitochondrial) proteins since they did purify these organelles. It will be highly relevant to report on this remaining part of the data set i) to indicate the specificity of the TMBIM5-imposed effect in the mitochondrial proteome (i.e. no effects observed in the extra-mitochondrial proteome), and ii) as a technical validation that small differences in protein turnover (as in Fig 4i) can be reliably determined (i.e. all proteins, or a random selection of 348 proteins to make a fair comparison, all lign up on the diagonal).

Response to referee #1:

The cellular proteome is shaped by the interplay of protein synthesis and protein degradation. In this study, the authors identified the mitochondrial inner membrane protein TMBIM5 as a regulating interaction factor of the m-AAA protease, the major protease of mitochondrial membrane proteins. TMBIM5 exhibits two activities: First, it serves as a Ca2+/H+ exchanger that couples the export of Ca2+ from mitochondria to the import of protons into mitochondria. The authors convincingly show that the loss of TMBIM5 leads to matrix alkalinization, mitochondrial hyperpolarization and the accumulation of Ca2+ within mitochondria, rendering cells sensitive to mitochondrial Ca2+ overload and cell death. In addition, TMBIM5 serves as binding factor of the m-AAA protease which chokes its proteolytic activity to prevent the m-AAA-mediated degradation of complex I proteins. Loss of TMBIM5 unleashes the m-AAA protease and promotes complex I turnover. Based on their data, the authors present an interesting model according to which TMBIM5 serves as an emergency valve which removes complex I once the levels of the membrane potential raise above a physiological range, thereby preventing hyperpolarization of the mitochondrial inner membrane.
This is an exciting study. It contains a wealth of interesting information, including a large number of proteomic analyses, all of which are well accessible from the excel data in the supplements. The study consists of three convincing parts describing the association of TIMBIM5 with AFG3L2, its function as a Ca2+/H+ exchanger and its relevance for complex I stability. Even though the model is compatible with the data shown, it also remains possible that TIMBIM5 plays some role in the biogenesis of complex I. According to this alternative 'assembly factor' model, its absence would also lead to an increased turnover of complex I subunits which would be prevented m-AAA protease activity is reduced. Such a function as a factor relevant for the assembly of membrane proteins was for example suggested for prohibitins, which - like TIMBIM5 - are AFG3L2 interactors. Even if some of the details of how TIMBIM5 influences complex I breakdown by the m-AAA protease remain unclear, this is an exciting study which will be of interest for a broad readership.

We thank the reviewer for their positive evaluation of our work and have performed a number of experiments to address their remaining concerns.

Main points
1. The authors should strengthen their evidence for a 'control factor function' rather than an 'assembly factor function' of TIMBIM5. For example, they show that upon knock down of AFG3L2, the levels of complex I subunits are partially restored. However, it remains unclear whether complex I is assembled and functional under these conditions. It should be straight forward to test this.

Following the suggestion of the reviewer, we have assessed the assembly of complex I by BN-PAGE combined with activity staining in WT and *TMBIM5*$^{-/-}$ cells upon siRNA-mediated depletion of AFG3L2. These experiments unambiguously demonstrate the

formation of enzymatically active complex I assembly in the absence of TMBIM5 (shown in new Fig. EV4E, F). We also observed a moderate increase in complex I activity upon knockdown of AFG3L2 in *TMBIM5*$^{-/-}$ cells, which however did not reach WT levels. This is consistent with the partial restoration of steady state levels of complex I subunits and likely explained by the multiple functions of AFG3L2, which regulates the assembly of mitochondrial ribosomes (via MRPL32) and other steps in mitochondrial gene expression.

Although these experiments already indicate that TMBIM5 is not required for complex I assembly, we substantiated this conclusion by BN-PAGE-based complexome profiling of WT and *TMBIM5*$^{-/-}$ HEK293 cells. Each BN-PAGE lane was cut into 72 slices of equal size followed by LC-MS/MS analysis. We calculated the iBAQ intensity and plotted the profile (protein intensity versus gel slice) and found that, independent of TMBIM5, the identified 40 OXPHOS complex I subunits assembled quantitatively into respiratory supercomplexes containing complexes I and III (new Fig. EV4A-C; new Table S4).

These results demonstrate that TMBIM5 is not essential for complex I assembly and further suggests that the observed reduced protein levels of complex I subunits in *TMBIM5*$^{-/-}$ cells results from unleashed degradation by AFG3L2.

2. The authors introduce and analyze the TMBIM5(D294K D325K) mutant as a variant that lacks the pH-driven calcium pumping activity. I wonder why this interesting variant is not employed to test whether the transporter activity can be uncoupled from the role as inhibitor of the m-AAA activity. One would expect that expression of this mutant would constantly inhibit AFG3L2, thereby being insensitive to oligomycin. If possible, data on the analysis of this mutant should be added to Fig. 6.

The analysis of cells expressing TMBIM5(D294K D325K) is hampered by the strong phenotype associated with this mutation, which may reflect constant inhibition of AFG3L2 (although other effects cannot be excluded). In an attempt to analyze this mutant further, we have transiently expressed TMBIM5 or TMBIM5(D294K D325K) in WT and *TMBIM5*$^{-/-}$ cells treated with oligomycin and assessed the stability of TMBIM5. However, transient transfection of cells resulted in strong overexpression of TMBIM and its mutant variant, which precluded to monitor protein turnover within 24 h (see reviewer figure 1). Therefore, it remains speculative whether the pH-insensitive variant of TMBIM5 inhibits AFG3L2 permanently, independent of the matrix pH.

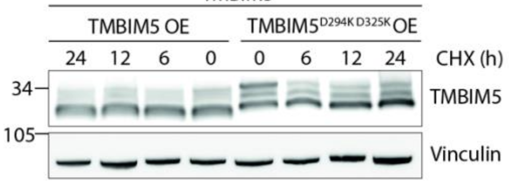

Minor points
3. Page 6, middle: '(Figure 1D, Figure S1G and' should be '(Figure 1D, Figure S1G) and'

We have corrected this typo.

4. Fig. 1. The sizes of the different panels are not perfect. Fig. 1E is far too small and therefore the cytosolic cytochrome c is hardly visible (without strong blow-up of the figure). Other panels (C, D, F) could be shrunk if space is limiting.

We have enlarged Fig. 1E and adjusted the size of the other panels in Fig. 1.

5. Fig. 2A. A small sketch would help to explain what this figure shows. As many readers might not be familiar with mtRED-GECO, a graphical description would help here.

We have added a sketch describing the experiments with mtRed-GECO as new Fig. 2B.

6. Fig. 2B. Is it possible that the change in pH is caused by the 'unleashed' m-AAA activity in the TMBIM5 mutants rather than by loss of TMBIM5-mediated proton pumping? Does knock down of AFG3L2 in TMBIM5 restore the pH of the matrix? According to the explanation of the authors, this would not be expected.

As suggested by the reviewer, we have monitored the pH of the matrix after depletion of AFG3L2. These experiments revealed that depletion of AFG3L2 did not restore the matrix pH in *TMBIM5*$^{-/-}$ cells, excluding the possibility that unleashed AFG3L2 activity causes the observed increase in matrix pH. These experiments are now shown in new Fig. EV4D.

Response to Referee #2:

This is an interesting paper, in which the authors identify TMBIM5 as a novel Ca2+/H+ exchanger of the inner mitochondrial membrane. They further demonstrate that TMBIM5 interacts with the m-AAA protease, that hyperpolarization triggers the loss of TMBIM5, and that the loss of TMBIM5 changes the composition of the mitochondrial proteome. They propose a model, in which TMBIM5 binds to m-AAA and is degraded by m-AAA upon hyperpolarization. Once TMBIM5 is degraded, m-AAA is released and then hydrolyzes multiple proteins in the inner membrane/matrix compartment. Thus, TMBIM5 integrates mitochondrial quality control with the bioenergetics status. The paper is novel, interesting, and significant. But there are two major issues and a number of minor issues that need to be addressed in a revision.

We thank the reviewer for their positive evaluation of our work and have performed a number of experiments to address their remaining concerns.

MAJOR ISSUES

1. The direct interaction between TMBIM5 and m-AAA is not sufficiently supported by the data. This should be acknowledged in the text of Results and Discussion. Specifically, the immunoprecipitation experiment cannot distinguish between a direct interaction between the two proteins and an indirect association mediated by other proteins. Also, the immunoprecipitation experiment should be described more extensively. It is not clear why digitonin was used for the solubilization of mitochondria because among commonly used detergents digitonin promotes indirect associations the most. Finally, in Fig 6D, the down-regulation of AFG3L2 did not stabilize TMBIM5 very much but AFG3L2-dependent degradation of TMBIM5 is crucial for the proposed model. In short, the weaknesses of those data should be acknowledged and the interpretation of those data adjusted accordingly.

We agree with the general notion of the reviewer that coimmunoprecipitation of two proteins does not allow to conclude unambiguously that they physically interact. This would require the determination of binding affinities between purified proteins *in vitro*, a challenging task for two multispanning membrane protein complexes. To account for this experimental limitation, we use now more careful wording throughout the manuscript and refer to TMBIM5 as a constituent of m-AAA protease containing complexes rather than a binding partner of AFG3L2. We also improved the description of the experimental procedure on p. 5. Digitonin is routinely used to solubilize mitochondrial membranes as it preserves also weak interactions between membrane proteins. As pointed out by the reviewer, however, it requires careful experimentation and control precipitations excluding unspecific interactions (that we have performed using cells expressing untagged AFG3L2, as now pointed out on p. 5).

We indeed observed only partial stabilization of TMBIM5 upon knock down of AFG3L2 (Fig. 6D) and explained this by the incomplete depletion of the enzyme. In agreement with AFG3L2 affecting the stability of TMBIM5, we show now that the abundance of TMBIM5 is strongly increased in *AFG3L2*$^{-/-}$ cells and that TMBIM5 is efficiently stabilized in these cells in emetine chase experiments (new Fig. EV5E, F). Moreover, we highlight now in the new Fig. 6E our quantitative mass spectrometric data showing that the steady state level of TMBIM5 decreases in hyperpolarized mitochondria, while it is stabilized upon depletion of AFG3L2. Together, these experiments provide further support that AFG3L2 degrades TMBIM5, although the participation of other proteases cannot be excluded. We state this now on p. 14/15 of the manuscript.

2. The analysis of the proteomics data is confusing and inconsistent within the paper. Comparing acute changes induced by a drug (oligomycin) to chronic changes induced by

protein deletion (TMBIM5-/-) does not make a lot of sense. Figures 6A and 6E are difficult to understand. Do the authors wish to conclude that oligomycin has an effect on wild-type cells but not on TMBIM5-/- cells ? If so, there should be an easier way to present that. Furthermore, the authors stress the point that complex I subunits are specifically affected but at the same time write "...that the loss of TMBIM5 causes broad reshaping of the mitochondrial proteome...". These two statements are mutually exclusive. Finally, it is not clear to me how the data in Fig 5C were calculated. In 5C, the axes should be re-labeled to indicate what the numeric data represent. Overall, the proteomics section should be simplified and scrutinized for internal inconsistencies.

We have carefully revised and simplified the description of our proteomic data to clarify the rationale and avoid misunderstandings. We agree with the reviewer that oligomycin induces hyperpolarization acutely, whereas it persists in *TMBIM5*$^{-/-}$ cells. However, we treated cells with oligomycin for an extended time period (16 h) and performed these experiments mainly to examine whether hyperpolarization is sufficient to explain the broad proteomic changes observed in *TMBIM5*$^{-/-}$ cells. This is clearly not the case. In contrast to the loss of TMBIM5, long-term oligomycin treatment induced only limited changes in the mitochondrial proteome, as we show in the Venn diagramme in Fig. 6A comparing proteomic changes induced by oligomycin-treatment of WT cells with those in untreated *TMBIM5*$^{-/-}$ cells (relative to untreated WT cells). We have improved the labelling of this diagramme for clarification.

This observation prompted us to analyze additional functions of TMBIM5 in further experiments. These experiments revealed that TMBIM5 inhibits AFG3L2 upon acute hyperpolarization of mitochondria (likely acting as competitive inhibitor). However, persistent hyperpolarization destabilizes TMBIM5 and unleashes AFG3L2 activity leading to a broad rewiring of the mitochondrial proteome.

We realize that the analysis of some of our proteomic experiments and their analysis has been complex and confusing. We therefore have now revised the description of these experiments and corrected confusing statements. As pointed out in the manuscript, depletion of AFG3L2 is known to interfere with the assembly of mitochondrial ribosomes and the synthesis of mitochondrially encoded OXPHOS subunits, which strongly inhibits cell growth, hampering the proteomic analysis of the effect of AFG3L2 depletion in *TMBIM5*$^{-/-}$ cells. To reduce the complexity of our proteomic dataset and clarify the main conclusion of these experiments, we´ve decided to omit the description of CHX chase experiments in these cells (previous Fig. 5C and related scheme in Fig. 5E). We now first identify proteins that are more rapidly degraded in *TMBIM5*$^{-/-}$ cells using SILAC (Fig. 4I) and then describe how depletion of AFG3L2 affects the steady state level of these proteins (p. 13). These experiments highlight that AFG3L2 broadly rewires the mitochondrial proteome, rather than specifically degrading complex I subunits. We also omitted the Venn diagramme previously shown in Fig. 6E to avoid confusion.

MINOR ISSUES
3. In Fig 2E, it should read "empty"
4. On p. 33, I mg/ml tetracycline seems very high
*5. On p. 40, µm*ml-1 is not a unit of concentration*

We have corrected these typos.

6. On p. 40, it is unclear what is meant by the "15 min swelling step" and what it is for

We have revised the description of this step in the Material and Method section.

7. On p. 43, the abbreviation NMDG has not been defined

The definition of the abbreviation for N-methyl-D-glutamate has been added.

8. Does the topology of TMBIM5 (inside out vs right side out) matter in the in vitro experiments? Please explain.

It depends on the characteristics of the channel, whether the insertion direction of reconstituted TMBIM5 matters for the outcome of the *in vitro* experiments. When determining the electrophysiological characteristics of TMBIM5, the current-voltage relationship shown in Fig. 3A displayed no signs of rectification, i.e. the ion-flux through the channel does not depend on the sign of the applied voltage. Hence, the channel appears to be conducting symmetrical and therefore the *in vitro* experiments do not depend on the insertion direction.

Response to Referee #3:

In this manuscript, the authors investigate the function and regulation of the m-AAA protease in mitochondria. They identify TMBIM5 as a Ca2+/H+ exchanger and a critical interactor of the m-AAA protease, regulating respiration and mitochondrial hyperpolarization. Functional interaction between m-AAA protease and m-AAA was shown during sustained hyperpolarization, resulting in degradation of TMBIM5 and subsequent remodeling of the mitochondrial proteome.

This is an intriguing and well-written paper, convincingly demonstrating how TMBIM5 functionally integrates mitochondrial Ca2+ signaling and energy status with remodeling of the mitochondrial proteome, to adjust cellular metabolism. Experiments were performed at a high technical level, including sophisticated proteomic experiments to investigate changes in protein turnover. I only have a few questions:

We thank the reviewer for their positive evaluation of our work and have performed a number of experiments to address their remaining concerns.

1. Fig 1a: What is the premise that candidate-binders should interact more prominently with catalytically inactive AFGL2? Or what was the negative control here: non-flagged mutated, or non-mutated (suggested by 'wt' as indicated in Fig 1)?

We apologize for being unclear here. We have used WT cells (lacking FLAG-tagged protein) as a negative control in these experiments. The rationale for using a proteolytic inactive variant of AFG3L2 as bait has been to ensure that both binding proteins and substrates can be identified. We have revised the description of this experiment for clarification.

2. Fig 4i: The authors perform protein turnover experiments, claiming that 140 proteins turned over 'significantly' faster in the absence of TMBIM5 (p. 11). Since the effect-size seems to be marginal for most proteins (slightly off the diagonal in Fig 4i), based on 2 (?) replicates, I wonder how significance was determined. There are two major points here: i) such experiments are typically prone to experimental variability resulting in deviations in half-life estimates, and ii) if the authors used conventional models for curve fitting, assuming exponential decay (no details are provided), this requires cells to be in steady state - which they are not. In addition, the authors introduce perturbations (KO of TMBIM5) that impact cellular metabolism/proliferation, formally making comparisons in half-life between conditions highly problematic. Therefore, more details should be provided how the data were treated/interpreted to arrive at the claims that are made.

We added more information about the mathematical modelling to the method and result part. Since we cultivated WT and *TMBIM5*$^{-/-}$ cells for at least 5 passages in SILAC medium, we indeed assume that the cells are in steady state. We also did not observe any difference in the growth of these cells (now shown in Appendix Panel C), indicating that proliferation does not cause a systematic increase in SILAC incorporation between WT and *TMBIM5*$^{-/-}$ cells. We would like to highlight that the <u>SILAC ratio was monitored over 7 timepoints (0, 2, 4, 6, 8, and 24 h) in 6 biological replicates</u>, which allowed us to identify an increased turnover of 140 proteins. The workflow is summarized in Appendix Panel B (formerly Fig. EV4J). We added this information to the text in the revised manuscript. To illustrate the difference in turnover, we show now in Appendix Panel E incorporation rates over time for one affected protein (NDUFA9), for one unaffected protein (NDUFB9) and for all identified Mitocarta 3.0 proteins (overall turnover rate unaffected). The statistical significance was assessed by a two-sided t-test using the estimated rate constant k for each individual replicate and a p-value cutoff of 0.01. Additionally, we plotted the distribution of r values obtained from the linear fit, which indicates the suitability of the peptide-based linear model and similar fits for WT and *TMBIM5*$^{-/-}$ cells (Reviewer Figure 2).

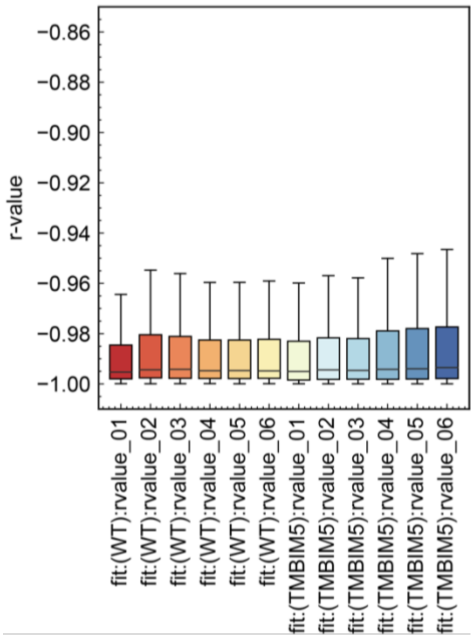

We revised the manuscript to ensure that the information about the general experimental design and the statistical analysis is precisely presented.

3. Related to the point above: the authors state that they determined turnover rates for 348 mitochondrial proteins (p.11), however their data must include many more (extra-mitochondrial) proteins since they did purify these organelles. It will be highly relevant to report on this remaining part of the data set i) to indicate the specificity of the TMBIM5-imposed effect in the mitochondrial proteome (i.e. no effects observed in the extra-mitochondrial proteome), and ii) as a technical validation that small differences in protein turnover (as in Fig 4i) can be reliably determined (i.e. all proteins, or a random selection of 348 proteins to make a fair comparison, all lign up on the diagonal).

Although we have analyzed lysates of WT and *TMBIM5*$^{-/-}$ cells and therefore obtained a complete view of turnover rates, we only described and discussed alterations in the mitochondrial proteome in the original manuscript. We included now additional information concerning non-mitochondrial proteins in the manuscript (p. 11 of the manuscript and Appendix Panel D).

We determined protein turnover rates of 987 proteins in total, including 348 MitoCarta 3.0 proteins. Proteome alterations in the absence of TMBIM5 are therefore not restricted to mitochondria. This is expected considering that the loss of TMBIM5 impairs mitochondrial OXPHOS activity and broadly affects the mitochondrial proteome (decreased steady state level of about 250 mitochondrial proteins), and considering the numerous functional interactions of mitochondria with their cellular environment. To comply with the request of the reviewer, we added a scatter plot of the turnover rate constants of significantly (p-value < 0.01) regulated mitochondrial and non-mitochondrial proteins in WT and *TMBIM5*$^{-/-}$ cells (now shown as Appendix Panel D).

1st Revision - Editorial Decision 18th May 2022

Dear Dr. Thomas Langer,

Thank you for submitting your revised manuscript (EMBOJ-2021-110476R) to The EMBO Journal. Your amended study was sent back to the three referees for their re-evaluation, and we have received comments from all of them, which I enclose below. As you will see, the reviewers stated that their issues have been comprehensively resolved and they are now broadly in favour of publication.

Thus, we are pleased to inform you that your manuscript has been accepted in principle for publication in The EMBO Journal.

We need you to take care of a number of minor issues related to formatting and data presentation as detailed below, which should be addressed at re-submission.

Please contact me at any time if you have additional questions related to below points.

As you might have noted on our web page, every paper at the EMBO Journal now includes a 'Synopsis', displayed on the html and freely accessible to all readers. The synopsis includes a 'model' figure as well as 2-5 one-short-sentence bullet points that summarize the article. I would appreciate if you could provide this figure and the bullet points.

Thank you for giving us the chance to consider your manuscript for The EMBO Journal. I look forward to your final revision.

Again, please contact me at any time if you need any help or have further questions.

with
Best regards,

Daniel Klimmeck

Daniel Klimmeck PhD
Senior Editor
The EMBO Journal

Formatting changes required for the revised version of the manuscript:

>>Please adjust the title of the 'Conflict of Interests' section to 'Disclosure and Competing Interests Statement'.

>> Remove the referee tokens from the data accessibility section and release privacy.

>> Funding annotation: LTFCOFUND2013; and GA-2013-609409, he Osamu Hayaishi Memorial Scholarship for Study Abroad and grants from the Uehara Memorial Foundation, DIP, RA1028/10-2 and CRC1218 - Project number 269925409' are missing in our online system. Please amend for consistency.

>> Author Contributions: E.R. is currently missing in our online system, no tick boxes selected in the Credit list. Please complete.

>> Dataset EV legends: Tables EV1-7 should be datasets and need their titles & legends added to the files.

>> Appendix file: list the one appendix figure in the table of contents and correct the nomenclature to 'Appendix Figure S1'; adjust callouts in the main text accordingly.

>> Figure callouts: There is a callout for Tables S6 and S8 but there are no such tables.

>> As to our journal policies, we kindly ask you to provide uncropped source data for the Western blots shown in Figures 1B,C; 2E; 5C; 6C,D; EV4 and EV5.

>> Please consider additional changes and comments from our production team as indicated by the .doc file enclosed and leave changes in track mode.

Further information is available in our Guide For Authors: https://www.embopress.org/page/journal/14602075/authorguide

We realize that it is difficult to revise to a specific deadline. In the interest of protecting the conceptual advance provided by the work, we recommend a revision within 3 months (16th Aug 2022). Please discuss the revision progress ahead of this time with the editor if you require more time to complete the revisions. Use the link below to submit your revision:

Link Not Available

Referee #1:

The authors satisfactorily addressed all points raised on the previous submission. I therefore recommend publication of this exciting study in EMBO Journal.

Referee #2:

In their revision, the authors addressed all my concerns satisfactorily. I am in full support of publication.

Referee #3:

The authors have appropriately addressed my concerns, and I recommend this exciting work to be accepted for publication.

The authors performed the requested editorial changes.

2nd Revision - Editorial Decision 1st Jul 2022

Dear Thomas,

Thank you for submitting the revised version of your manuscript. I have now evaluated your amended manuscript and concluded that the remaining minor concerns have been sufficiently addressed.

Thus, I am pleased to inform you that your manuscript has been accepted for publication in the EMBO Journal.

Please note that it is EMBO Journal policy for the transcript of the editorial process (containing referee reports and your response letter) to be published as an online supplement to each paper. I would thus like to ask for your consent on keeping the additional referee figure included in this file.

Also, in case you might NOT want the transparent process file published at all, you will also need to inform us via email immediately. More information is available here:
https://www.embopress.org/page/journal/14602075/authorguide#transparentprocess

----

Please note that in order to be able to start the production process, our publisher will need and contact you shortly regarding the page charge authorisation and licence to publish forms.

Authors of accepted peer-reviewed original research articles may choose to pay a fee in order for their published article to be made freely accessible to all online immediately upon publication. The EMBO Open fee is fixed at €5,500 EUR (+ VAT where applicable).

We offer two licenses for Open Access papers, CC-BY and CC-BY-NC-ND.
For more information on these licenses, please visit:
http://creativecommons.org/licenses/by-nc-nd/3.0/deed.en_US

Notably, please be reminded that under the DEAL agreement of European scientific institutions with our publisher Wiley, you could be eligible for free publication of your article in the open access format. Please contact either the administration at your institution or Wiley (embojournal@wiley.com) to clarify further questions.

Should you be planning a Press Release on your article, please get in contact with embojournal@wiley.com as early as possible, in order to coordinate publication and release dates.

On a different note, I would like to alert you that EMBO Press is currently developing a new format for a video-synopsis of work published with us, which essentially is a short, author-generated film explaining the core findings in hand drawings, and, as we believe, can be very useful to increase visibility of the work. This has proven to offer a nice opportunity for exposure i.p. for the first author(s) of the study. Please see the following link for representative examples and their integration into the article web page:
https://www.embopress.org/video_synopses
https://www.embopress.org/doi/full/10.15252/embj.2019103932

Please let me know, should you be interested to engage in commissioning a similar video synopsis for your work. According operation instructions are available and intuitive.

Finally, we have noted that the submitted version of your article is also posted on the preprint platform bioRxiv. We would appreciate if you could alert bioRxiv on the acceptance of this manuscript at The EMBO Journal in order to allow for an update of the entry status. Thank you in advance!

If you have any questions, please do not hesitate to call or email the Editorial Office.

Thank you for this contribution to The EMBO Journal and congratulations on a successful publication!

Please consider us again in the future for your most exciting work.

Kind regards,

Daniel

Daniel Klimmeck, PhD
Senior Editor
The EMBO Journal
EMBO
Postfach 1022-40
Meyerhofstrasse 1
D-69117 Heidelberg
contact@embojournal.org
Submit at: http://emboj.msubmit.net

EMBO Press Author Checklist

Corresponding Author Name: Stefan Feske & Carl Weidinger	
Journal Submitted to: Embo Mol Med	
Manuscript Number: EMM-2022-15687	

USEFUL LINKS FOR COMPLETING THIS FORM
The EMBO Journal - Author Guidelines
EMBO Reports - Author Guidelines
Molecular Systems Biology - Author Guidelines
EMBO Molecular Medicine - Author Guidelines

Reporting Checklist for Life Science Articles (updated January 2022)
This checklist is adapted from Materials Design Analysis Reporting (MDAR) Checklist for Authors. MDAR establishes a minimum set of requirements in transparent reporting in the life sciences (see Statement of Task: 10.31222/osf.io/9sm4x). Please follow the journal's guidelines in preparing your manuscript.
Please note that a copy of this checklist will be published alongside your article.

Abridged guidelines for figures
1. Data
The data shown in figures should satisfy the following conditions:
- the data were obtained and processed according to the field's best practice and are presented to reflect the results of the experiments in an accurate and unbiased manner.
- ideally, figure panels should include only measurements that are directly comparable to each other and obtained with the same assay.
- plots include clearly labeled error bars for independent experiments and sample sizes. Unless justified, error bars should not be shown for technical replicates.
- if n<5, the individual data points from each experiment should be plotted. Any statistical test employed should be justified.
- Source Data should be included to report the data underlying figures according to the guidelines set out in the authorship guidelines on Data Presentation.

2. Captions
Each figure caption should contain the following information, for each panel where they are relevant:
- a specification of the experimental system investigated (eg cell line, species name).
- the assay(s) and method(s) used to carry out the reported observations and measurements.
- an explicit mention of the biological and chemical entity(ies) that are being measured.
- an explicit mention of the biological and chemical entity(ies) that are altered/varied/perturbed in a controlled manner.
- the exact sample size (n) for each experimental group/condition, given as a number, not a range;
- a description of the sample collection allowing the reader to understand whether the samples represent technical or biological replicates (including how many animals, litters, cultures, etc.).
- a statement of how many times the experiment shown was independently replicated in the laboratory.
- definitions of statistical methods and measures:
  - common tests, such as t-test (please specify whether paired vs. unpaired), simple χ2 tests, Wilcoxon and Mann-Whitney tests, can be unambiguously identified by name only, but more complex techniques should be described in the methods section;
  - are tests one-sided or two-sided?
  - are there adjustments for multiple comparisons?
  - exact statistical test results, e.g., P values = x but not P values < x;
  - definition of 'center values' as median or average;
  - definition of error bars as s.d. or s.e.m.

Please complete ALL of the questions below.
Select "Not Applicable" only when the requested information is not relevant for your study.

Materials

Newly Created Materials	Information included in the manuscript?	In which section is the information available? (Reagents and Tools Table, Materials and Methods, Figures, Data Availability Section)
New materials and reagents need to be available; do any restrictions apply?	Not Applicable	No newly created materials were used within this manuscript

Antibodies	Information included in the manuscript?	In which section is the information available? (Reagents and Tools Table, Materials and Methods, Figures, Data Availability Section)
For **antibodies** provide the following information:<br>- Commercial antibodies: RRID (if possible) or supplier name, catalogue number and or/clone number<br>- Non-commercial: RRID or citation	Yes	All informations including supplier names, clone numbers and fluochromes are provided in the Appendix

DNA and RNA sequences	Information included in the manuscript?	In which section is the information available? (Reagents and Tools Table, Materials and Methods, Figures, Data Availability Section)
**Short novel DNA or RNA including primers, probes**: provide the sequences.	Yes	All sequences of RNA primers are provided in the Materials and Methods section

Cell materials	Information included in the manuscript?	In which section is the information available? (Reagents and Tools Table, Materials and Methods, Figures, Data Availability Section)
**Cell lines:** Provide species information, strain. Provide accession number in repository **OR** supplier name, catalog number, clone number, and/**OR** RRID.	Yes	The information on human HEK cells is provided in the Materials and Methods section
**Primary cultures:** Provide species, strain, sex of origin, genetic modification status.	Yes	The information is porvided in the Materials and Methods section
Report if the cell lines were recently **authenticated** (e.g., by STR profiling) and tested for mycoplasma contamination.	Not Applicable	-

Experimental animals	Information included in the manuscript?	In which section is the information available? (Reagents and Tools Table, Materials and Methods, Figures, Data Availability Section)
**Laboratory animals or Model organisms:** Provide species, strain, sex, age, genetic modification status. Provide accession number in repository **OR** supplier name, catalog number, clone number, **OR** RRID.	Yes	All relevant information is provoded in the Materials and Methods section
**Animal observed in or captured from the field:** Provide species, sex, and age where possible.	Not Applicable	
Please detail **housing and husbandry conditions**.	Yes	Animal conditions are specified in the Materials and Methods section

Plants and microbes	Information included in the manuscript?	In which section is the information available? (Reagents and Tools Table, Materials and Methods, Figures, Data Availability Section)
**Plants:** provide species and strain, ecotype and cultivar where relevant, unique accession number if available, and source (including location for collected wild specimens).	Not Applicable	No plants were used in this study
**Microbes:** provide species and strain, unique accession number if available, and source.	Not Applicable	no microbes were used in this study

Human research participants	Information included in the manuscript?	In which section is the information available? (Reagents and Tools Table, Materials and Methods, Figures, Data Availability Section)
If collected and within the bounds of privacy constraints report on age, sex and gender or ethnicity for all study participants.	Yes	Patients characteristics such as Age, Sex and Disease entity are detailed in the Appendix (Supplementary Table 1)

Core facilities	Information included in the manuscript?	In which section is the information available? (Reagents and Tools Table, Materials and Methods, Figures, Data Availability Section)
If your work benefited from core facilities, was their service mentioned in the acknowledgments section?	Yes	Core Facilties are mentioned in the acknowledgements section

Design

Study protocol	Information included in the manuscript?	In which section is the information available? (Reagents and Tools Table, Materials and Methods, Figures, Data Availability Section)
If study protocol has been **pre-registered, provide DOI in the manuscript**. For clinical trials, provide the trial registration number **OR** cite DOI.	Not Applicable	the study was not pre-registerd as it is not a clinical study
Report the **clinical trial registration number** (at ClinicalTrials.gov or equivalent), where applicable.	Not Applicable	The the study is a basic research study and not a clinical study

Laboratory protocol	Information included in the manuscript?	In which section is the information available? (Reagents and Tools Table, Materials and Methods, Figures, Data Availability Section)
Provide DOI OR other citation details if **external detailed step-by-step protocols** are available.	Not Applicable	

Experimental study design and statistics	Information included in the manuscript?	In which section is the information available? (Reagents and Tools Table, Materials and Methods, Figures, Data Availability Section)
Include a statement about **sample size** estimate even if no statistical methods were used.	Yes	A statement about the sample size estimate for CyTOF analyses has been included in the methods section
Were any steps taken to minimize the effects of subjective bias when allocating animals/samples to treatment (e.g. **randomization procedure**)? If yes, have they been described?	Not Applicable	No randomization steps were performed
Include a statement about **blinding** even if no blinding was done.	Yes	All histologic analyses were performed in a blinded manner. This step has been described in the Materials and Methods section.
Describe **inclusion/exclusion criteria** if samples or animals were excluded from the analysis. Were the criteria pre-established?<br><br>If sample or data points were omitted from analysis, report if this was due to attrition or intentional exclusion and provide justification.	Not Applicable	No data points were omitted from analyses
For every figure, are **statistical tests** justified as appropriate? Do the data meet the assumptions of the tests (e.g., normal distribution)? Describe any methods used to assess it. Is there an estimate of variation within each group of data? Is the variance similar between the groups that are being statistically compared?	Yes	All statitsic tests are described in the Materials and Methods sections and the Figure legends

Sample definition and in-laboratory replication	Information included in the manuscript?	In which section is the information available? (Reagents and Tools Table, Materials and Methods, Figures, Data Availability Section)
In the figure legends: state number of times the experiment was **replicated** in laboratory.	Yes	Informations are provided in the figure legends
In the figure legends: define whether data describe **technical or biological replicates**.	Yes	Imformations are provided in the figure legends

Ethics

Ethics	Information included in the manuscript?	In which section is the information available? (Reagents and Tools Table, Materials and Methods, Figures, Data Availability Section)
Studies involving **human participants**: State details of **authority granting ethics approval** (IRB or equivalent committee(s), provide reference number for approval.	Yes	The reference number and the authoriy granting the ethics approval has been stated in the Materials and Methods section
Studies involving **human participants**: Include a statement confirming that **informed consent** was obtained from all subjects and that the experiments conformed to the principles set out in the WMA Declaration of Helsinki and the Department of Health and Human Services Belmont Report.	Yes	A statement has been included in to the Materials and Methods section
Studies involving **human participants:** For publication of **patient photos**, include a statement confirming that consent to publish was obtained.	Not Applicable	No photos of patients are included
Studies involving experimental **animals**: State details of **authority granting ethics approval** (IRB or equivalent committee(s), provide reference number for approval. Include a statement of compliance with ethical regulations.	Yes	Details are provided in the Materials and Methods section
Studies involving **specimen and field samples:** State if relevant **permits** obtained, provide details of authority approving study; if none were required, explain why.	Not Applicable	

Dual Use Research of Concern (DURC)	Information included in the manuscript?	In which section is the information available? (Reagents and Tools Table, Materials and Methods, Figures, Data Availability Section)
Could your study fall under dual use research restrictions? Please check biosecurity documents and list of **select agents and toxins** (CDC): https://www.selectagents.gov/sat/list.htm	Not Applicable	
If you used a select agent, is the security level of the lab appropriate and reported in the manuscript?	Not Applicable	
If a study is subject to dual use research of concern regulations, is the name of the **authority granting approval and reference number** for the regulatory approval provided in the manuscript?	Not Applicable	

Reporting

The MDAR framework recommends adoption of discipline-specific guidelines, established and endorsed through community initiatives. Journals have their own policy about requiring specific guidelines and recommendations to complement MDAR.

Adherence to community standards	Information included in the manuscript?	In which section is the information available? (Reagents and Tools Table, Materials and Methods, Figures, Data Availability Section)
State if relevant guidelines or checklists (e.g., **ICMJE, MIBBI, ARRIVE, PRISMA**) have been followed or provided.	Not Applicable	
For **tumor marker prognostic studies,** we recommend that you follow the **REMARK** reporting guidelines (see link list at top right). See author guidelines, under 'Reporting Guidelines'. Please confirm you have followed these guidelines.	Not Applicable	
For **phase II and III randomized controlled trials**, please refer to the **CONSORT** flow diagram (see link list at top right) and submit the CONSORT checklist (see link list at top right) with your submission. See author guidelines, under 'Reporting Guidelines'. Please confirm you have submitted this list.	Not Applicable	

Data Availability

Data availability	Information included in the manuscript?	In which section is the information available? (Reagents and Tools Table, Materials and Methods, Figures, Data Availability Section)
Have **primary datasets** been deposited according to the journal's guidelines (see 'Data Deposition' section) and the respective accession numbers provided in the Data Availability Section?	Yes	This study does not contain data that requires deposition in a public database. A statement has been provided in the Data Availability Section
Were **human clinical and genomic datasets** deposited in a public access-controlled repository in accordance to ethical obligations to the patients and to the applicable consent agreement?	Not Applicable	No human clinical or genomic data sets were deposited
Are **computational models** that are central and integral to a study available without restrictions in a machine-readable form? Were the relevant accession numbers or links provided?	Not Applicable	
If publicly available data were reused, provide the respective **data citations in the reference list**.	Yes	Citations for publicly available data sets are provided in the reference list
